# Genetic characteristics of complete mtDNA genome sequence of Indonesian local rabbit (*Oryctolagus cuniculus*)

**DOI:** 10.1186/s43141-023-00546-1

**Published:** 2023-10-09

**Authors:** Asep Setiaji, Dela Ayu Lestari, Nuruliarizki Shinta Pandupuspitasari, Ikania Agusetyaningsih, Faheem Ahmed Khan

**Affiliations:** 1 Department of Animal Science, Faculty of Animal and Agricultural Sciences, Universitas Diponegoro, Semarang, 50275 Indonesia; 2 Research Center for Animal Husbandry, National Research and Innovation Agency (BRIN), South Tangerang, 15314 Indonesia; 3 Faculty of Science and Technology, University of Central Punjab, Lahore, 54000 Pakistan

**Keywords:** mtDNA, *Oryctolagus cuniculus*, WGS

## Abstract

**Background:**

Indonesian local rabbit (*Oryctolagus cuniculus*) is a local breed in Indonesia. We reveal the mitochondrial genome sequence of the Indonesian local Rabbit for the first time. A better understanding of the mechanisms underlying these beneficial aspects of local breeds over imported ones requires detailed genetic investigations, of which mtDNA genome sequencing is of particular importance. Such an investigation will solve the major issues of misidentification with Javanese hares (Lepus nigricollis) and maternal lineage. In addition, this information will guide better statistics on the Indonesian local rabbit breed population and strategies for its conservation and breeding plans. This study aimed to identify and explore the characteristics of the mtDNA genomes of Indonesian local rabbits.

**Result:**

This study observed that the length of the mtDNA genome is 17,469 bp, consisting of two ribosomal RNA (12S rRNA, 16S rRNA), 22 transfer RNA genes (trnR, trnG, trnK, trnD, trnS, trnY, trnC, trnN, trnA, trnW, trnM, trnQ, trnl, trnL, trnV, trnF, trnP, trnT, trnE, trnL, trnS, trnH), 13 protein-coding genes (PCGs) (ND4l, ND3, COX3, ATP6, ATP8, COX2, COX1, ND2, ND1, CYTB, ND6, ND5, ND4), a replication origin, and a noncoding control region (D-loop).

**Conclusions:**

mtDNA genome of Indonesian local rabbit was the longest and had the most extended D-loop sequence among the other references of *Oryctolagus cuniculus*. Other specific differences were also found in the percentage of nucleotides and variation in most of the PCGs when they were aligned with *Oryctolagus cuniculus* references from GenBank. Indonesian local Rabbits strongly suspected brought from Europe during the colonial period in Indonesia.

**Supplementary Information:**

The online version contains supplementary material available at 10.1186/s43141-023-00546-1.

## Background

Rabbit (*Oryctolagus cuniculus*) has high economic value and a significant contribution to food and livelihood security, with the potential to be raised at the commercial level. Rabbit breeds in Indonesia are dominated by imported rabbit breeds and their crossbreeds, such as New Zealand White, Rex, Flemish Giant, Satin, and Angora [1]. Indonesian local rabbit breeds have been observed to have higher adaptability to tropical environmental conditions, with high-quality meat production, disease resistance, and better breeding ability [2]. Indonesian Local rabbits are included in medium-sized rabbits. They have coarse textured fur. Their adult body weight reaches only 2.5 kg. Their colors covers from white, brown, black and combinations. Local rabbits are thought to have come from Dutch rabbits brought from the Netherlands around 100 years ago. The male of Indonesian local rabbit breed was observed had a high genetic potential in the crossing with imported ones [3].

A better understanding of the mechanisms underlying these beneficial aspects of local breeds over imported ones requires detailed genetic investigations, of which mtDNA genome sequencing is of particular importance. Mitochondria is essential for metabolism, apoptosis [4], disease [5] and aging [6]. They carry out oxidative phosphorylation, which is necessary for the synthesis of ATP as well as a number of other metabolic processes. These subcellular organelles include a genome known as mitochondrial DNA (mtDNA), which is distinct from the nuclear chromatin and is frequently employed in studies of molecular phylogenetics [7]. Animal mtDNA typically has a short (15–20 kb) genome with 37 genes. Even while significantly bigger mitochondrial genomes have occasionally been identified, they are the result of mtDNA duplications rather than changes in gene composition [8, 9]. Thirteen protein subunits of the oxidative phosphorylation enzymes are encoded by a typical gene complement. Twenty-two tRNAs and ttwo rRNAs of the mitochondrial ribosome required for the translation of the proteins encoded by the mtDNA [10].

The mtDNA sequencing is of paramount importance in determining the maternal lineage and tracing the origin of locally available local rabbit breeds. In addition, it can determine the genetic distance and genetic relationship between Indonesian local rabbits and other rabbit breeds. White et al. [11] demonstrated that the genetic relationships of an organism can be investigated by genes that are maternally inherited by the offspring, as such genes do not experience recombination. It is to be kept in mind that till date only mtDNA sequences can be used to trace the origin of an organism, estimate the population diversity, determine conservation status, calculate genetic distance, and determine genetic relationships between subpopulations. This study aimed to identify and explore the characteristics of the mtDNA genomes of Indonesian local rabbits. Such an investigation will solve the major issues of misidentification with Javanese hares (*Lepus nigricollis*) and maternal lineage. In addition, this information will guide better statistics on the local rabbit breed population and strategies for its conservation and breeding plans. The results of this study are expected to provide information for additional examination of mtDNA sequences in favor of rabbit genetic research. Additionally, mtDNA genome sequencing analysis offers a fresh perspective on the genetic makeup of rabbits.

## Methods

### Ethical approval

The experimental procedures were approved by the Animal Research Ethics Committee of the Faculty of Animal and Agricultural Sciences, Universitas Diponegoro (No. 59–01/A-01/KEP-FPP).

### Tissue collection and DNA extraction

This study used gDNA from an Indonesian local rabbit (*Oryctolagus cuniculus*) isolated from the liver tissue. The rabbit was slaughtered and dissected to collect the liver tissue. Tissue samples (10 g) were stored in Falcon tubes containing ethanol. Tissue samples were used to obtain gDNA. gDNA was extracted according to the manufacturer’s standard protocol using the Quick-DNA Magbead Plus Kit (Zymo Research, USA). The collected gDNA was then selected based on quality and quantity, and genomic mtDNA enrichment was conducted using the REPLI-g Mitochondrial DNA Kit (Qiagen, Germany). The library preparation process used the enhanced mtDNA.

### WGS mtDNA sequencing and bioinformatic analysis

**Fig. 1:**
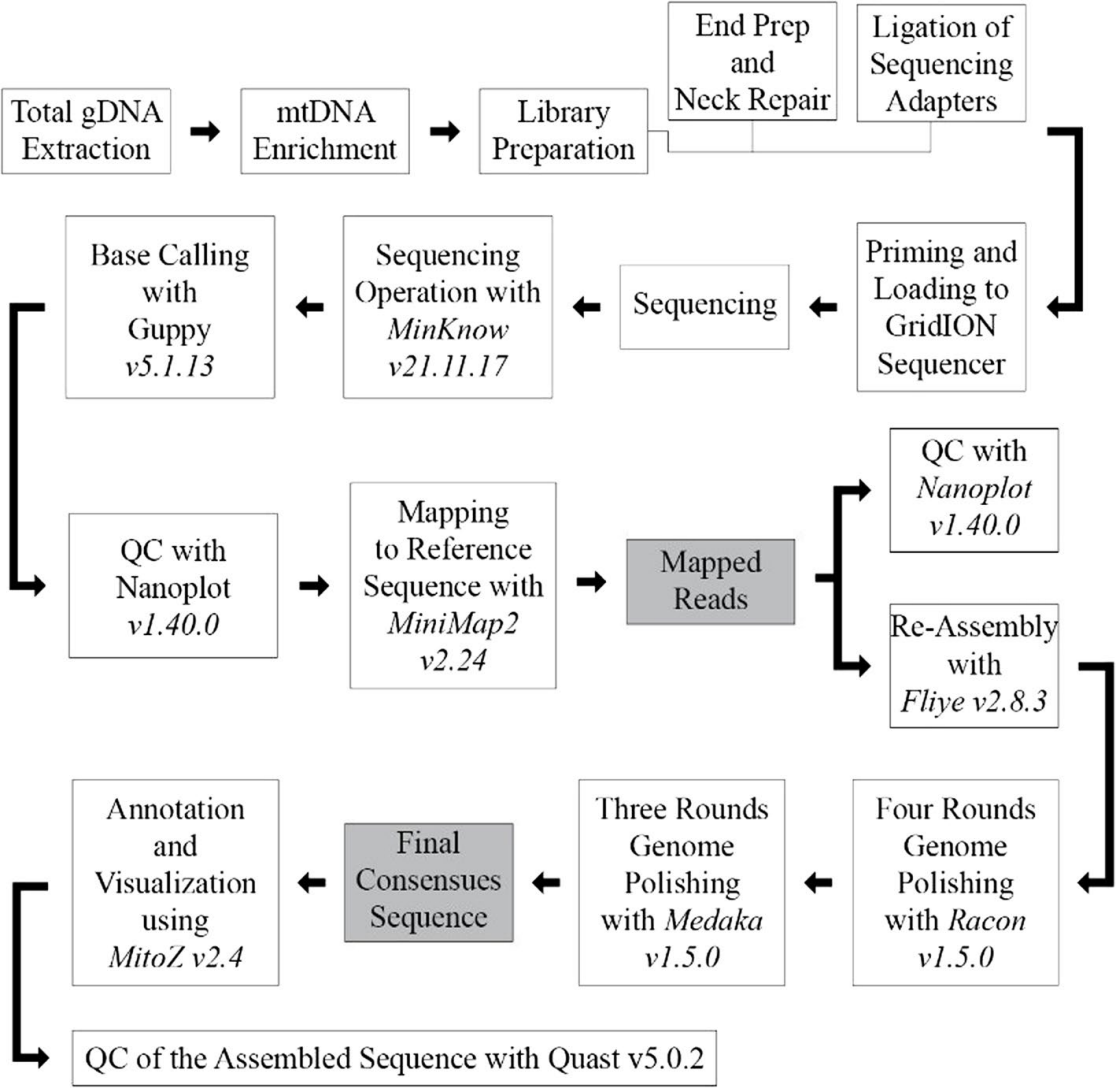
Workflow procedure for genome mtDNA and bioinformatics analysis

mtDNA sequencing was performed using WGS mtDNA analysis with nanopore technology [12, 13] by Oxford Nanopore Technologies GidION by PT. Genetika Science. Bioinformatics analysis was then performed. The workflow procedure for WGS, mtDNA, and bioinformatic analysis is shown in Fig. 1. The MinKNOW (v21.11.17) program was used to run the sequencing output from the Oxford Nanopore Technologies GridION sequencing. Guppy (v5.1.13) performed base calling in high-accuracy mode [14]. NanoPlot (v1.40.0) was used to visualize read quality [15]. Using minimap2 (v2.24), all readings were mapped to the mitochondrial reference sequence of *Oryctolagus cuniculus* from GenBank with accession number AJ001588 (New Zealand White / NZW1 rabbit by Gissi et al. [16]); MN953621 (Chuanbai Rex/CR rabbit) by Wang et al. [17]); MH985853 (New Zealand White / NZW2 rabbit by Hu et al. [18]) and MN296708 (Yimeng Wool / YW rabbit by Yao et al. [19]). Flye (v2.8.3) was used to perform the assembly using the filtered mapped reads [20]. A Nanoplot (v1.40.0) was used to assess the quality of mapped and filtered reads. Racon (v1.5.0) was used to polish the constructed sequence four times and Medaka (v1.5.0) was used three times ([21]; https://github.com/nanoporetech/medaka). MitoZ (v2.4) was used to annotate and visualize the final sequences [22]. Quast (v5.0.2) assessed the quality of constructed sequences [23].

### Data analysis

Data analysis was conducted using the MEGA11 software [24]. Complete mtDNA sequences were aligned to identify the genetic characteristics of mtDNA in Indonesian rabbits, including size, nucleotide composition diversity and mutation. The sequence alignment was also used to visualize the phylogenetic tree. The phylogenetic tree was constructed using the Maximum Likelihood method [25] based on Indonesian local rabbit mtDNA complete sequences from this study and 19 sequences from GenBank consisting of Oryctolagus, Lepus, Cavia, Ochotona, and Brachylagus as a comparator (Table 1).

**Table 1:** References obtained from genbank as comparator

No	Organism Name	Accession Number
1	*Oryctolagus cuniculus*	AJ001588
2	*Oryctolagus cuniculus* (Chuanbai Rex rabbit)	MN953621
3	*Oryctolagus cuniculus* (New Zealand White rabbit)	MH985853
4	*Oryctolagus cuniculus* (Yimeng Wool rabbit)	MN296708
5	*Lepus capensis*	GU937113.1
6	*Lepus coreanus*	KF040450.1
7	*Lepus europaeus*	AJ421471
8	*Lepus europaeus*	KY211025.1
9	*Lepus granatensis*	KJ397610.1
10	*Lepus hainanus*	JQ219662.1
11	*Lepus sinensis*	KM362831.1
12	*Lepus timidus*	KR030072.1
13	*Lepus tolai*	KM609214
14	*Lepus tolai*	MN539744.1
15	*Lepus yarkadensis*	MN539747
16	*Lepus yarkadensis*	MN450151.1
17	*Cavia porcellus*	MT017565
18	*Ochotona princeps*	NC_005358
19	*Brachylagus idahoensis*	OL436257

## Result

### mtDNA genome features of the Indonesian local rabbit

**Fig. 2:**
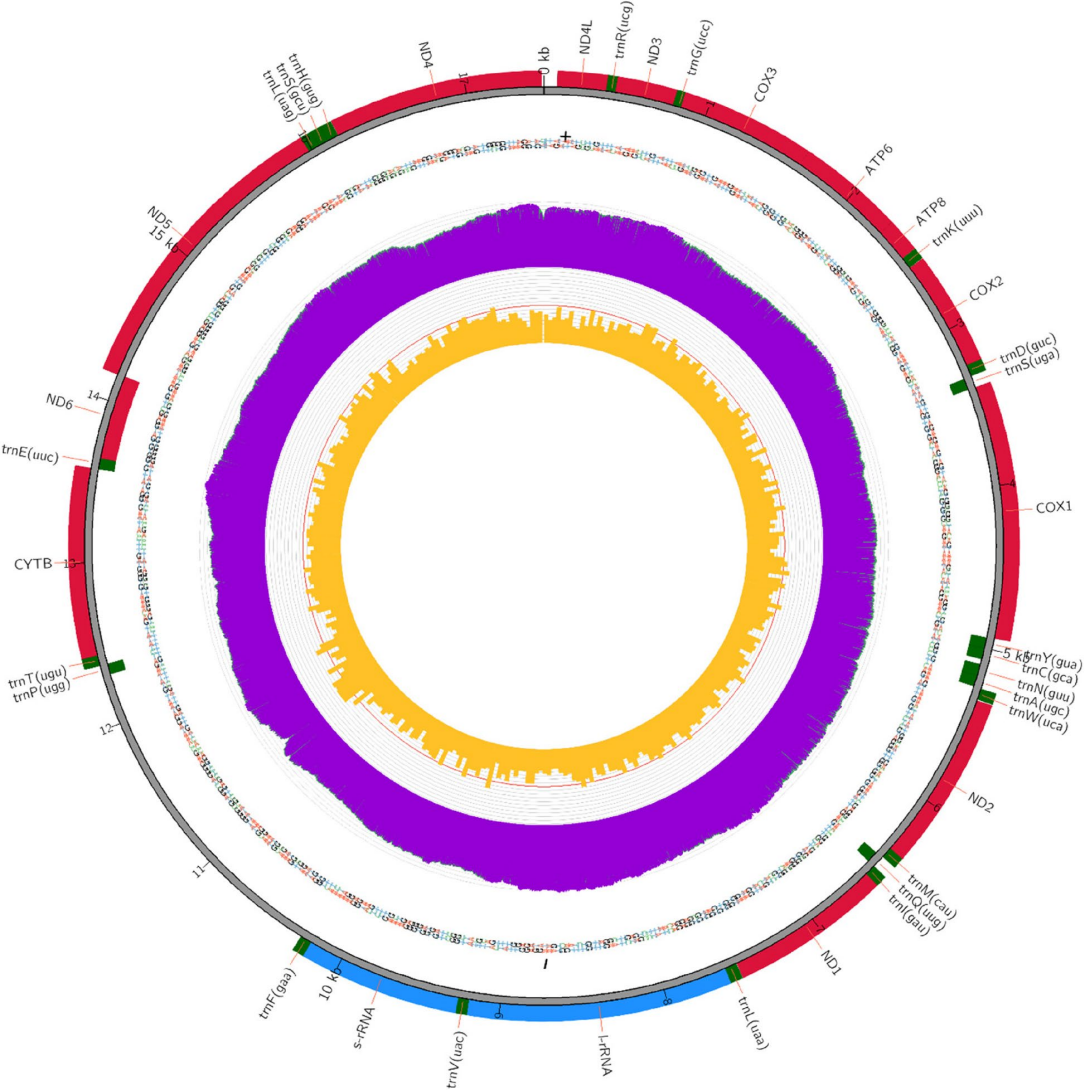
Complete map sequence of genome mtDNA of Indonesian Local Rabbit

The results of the analysis showed that the mtDNA genome length of Indonesian local rabbits was 17,469 bp, consisting of two ribosomal RNA (12S rRNA, 16S rRNA), 22 transfer RNA genes (trnR, trnG, trnK, trnD, trnS, trnY, trnC, trnN, trnA, trnW, trnM, trnQ, trnl, trnL, trnV, trnF, trnP, trnT, trnE, trnL, trnS, trnH), 13 protein-coding genes (PCGs) (ND4l, ND3, COX3, ATP6, ATP8, COX2, COX1, ND2, ND1, CYTB, ND6, ND5, ND4), a replication origin, and a noncoding control region (D-loop) (Fig. 2, Table 2). The positions of trnS, trnY, trnC, trnN, trnA, trnQ, trnE, and ND6 are in the heavy strand (H), while the rest are in the light strand (L). Moreover, a replication origin of the light strand was 33 bp in length and between trnN and trnC. The nucleotide composition in this study showed that thymine (T) was the most abundant (31.4%), followed by adenine (A) (28.3%), guanine (G) (26.7%), and cytosine (C) (13.6%) (Table 3.). The genes with the highest G, C, T, and A compositions were trnP (ugg) (34.8%), ND1 (30.0%), ND6 (40.2%), and trnR (40.3%), respectively. In contrast, the lowest compositions of G, C, T, and A among the genes were ATP8 (7.8%), ND6 (6.7%), trnF (21.7%), and ND6 (19.8%), respectively.

**Table 2:** References used in this study that was obtained from Genbank as comparator

Gene	Position		Size (bp)	Nucleotide (%)				Amino Acid			Strand^a^
	Start	End		G	C	T	A	Length	Start Codon	Stop Codon	
trnF(gaa)	1	69	69	17.4	23.2	21.7	37.7				L
12S rRNA	70	1026	957	18.2	21.9	24.0	35.8				L
trnV(uac)	1027	1092	66	16.7	25.8	27.3	30.3				L
16S rRNA	1091	2671	1581	17.8	20.4	25.4	36.4				L
trnL(uaa)	2672	2746	75	18.7	22.7	26.7	32.0				L
ND1	2749	3705	957	12.3	30.0	28.9	28.7	318	ATG	TAG	L
trnl(gau)	3704	3773	70	17.1	11.4	34.3	37.1				L
trnQ(uug)	3771	3842	72	29.2	13.9	31.9	25.5				H
trnM(cau)	3851	3919	69	18.8	24.6	27.5	29.0				L
ND2	3920	4963	1044	9.1	27.7	28.3	35.0	347	ATT	TAA	L
trnW(uca)	4971	5037	67	19.4	23.9	22.4	34.3				L
trnA(ugc)	5040	5106	67	22.4	14.9	32.8	29.9				H
trnN(guu)	5107	5179	73	28.8	17.8	28.8	24.7				H
replication origin^b^	5180	5212	33	27.3	24.2	12.1	36.4				L
trnC(gca)	5213	5281	69	24.6	21.7	24.6	29.0				H
trnY(gua)	5282	5347	66	22.7	16.7	27.3	33.3				H
COX1	5355	6896	1542	17.4	25.2	31.0	26.4	513	ATG	TAG	L
trnS(uga)	6899	6967	69	27.5	17.4	33.3	21.7				H
trnD(guc)	6971	7039	69	10.1	14.5	39.1	36.2				L
COX2	7040	7723	684	14.5	26.6	28.7	30.3	227	ATG	TAG	L
trnK(uuu)	7727	7795	69	23.2	18.8	26.1	31.9				L
ATP8	7797	8000	204	7.8	29.4	28.4	34.3	67	ATG	TAA	L
ATP6	7958	8638	681	11.3	29.7	29.5	29.5	226	ATG	TAA	L
COX3	8636	9441	804	15.2	28.5	29.1	27.2	267	ATG	TAG	L
trnG(uuc)	9422	9491	70	14.3	22.9	30.0	32.9				L
ND3	9492	9848	357	11.5	23.8	33.1	31.7	118	ATA	TAG	L
trnR(ucg)	9839	9905	67	13.4	10.4	35.8	40.3				L
ND4L	9908	10204	297	10.8	25.6	34.3	29.3	98	ATG	TAA	L
ND4	10198	11572	1378	11.5	29.5	29.3	29.6	459	ATT	TAT	L
trnH(gug)	11575	11643	69	14.5	17.4	30.4	37.7				L
trnS(gcu)	11644	11702	59	16.9	28.8	28.8	25.4				L
trnL(uag)	11703	11772	70	20.0	14.3	25.7	40.0				L
ND5	11773	13584	1812	11.4	27.1	30.6	30.8	603	ATT	TAG	L
ND6	13580	14104	525	33.3	6.7	40.2	19.8	174	ATG	AGG	H
trnE(uuc)	14105	14172	68	17.6	14.7	36.8	30.9				H
CYTB	14176	15315	1140	12.8	20.4	28.9	27.9	379	ATG	AGG	L
trnT(ugu)	15315	15380	66	22.7	19.7	28.8	28.8				L
trnP(ugg)	15381	15446	66	34.8	15.2	28.8	21.2				L
D-loop	15447	17469	2022	12.8	28.8	26.8	31.6				

^a^ *L* light strand, *H* heavy strand

^b^Origin of L-strand replication

**Table 3:** Size and nucleotide percentage of Indonesian local rabbit mtDNA in this study and other references

No	Organism Name	Accession Number	Size	Nucleotide (%)				References
				G	C	T	A	
1	*Oryctolagus cuniculus* (Indonesian local rabbit)	-	17.469	26.7	13.6	31.4	28.3	This study
2	*Oryctolagus cuniculus*	AJ001588	17.245	13.6	26.6	28.3	31.5	[16]
3	*Oryctolagus cuniculus* (Chuanbai Rex)	MN953621	17.174	13.6	26.6	28.4	31.5	[17]
4	*Oryctolagus cuniculus* (New Zealand White)	MH985853	17.418	13.6	26.6	28.3	31.4	[18]
5	*Oryctolagus cuniculus* (Yimeng Wool)	MN296708	16.740	13.7	26.6	28.2	31.5	[19]
6	*Lepus capensis*	GU937113.1	17.722	13.2	25.6	29.5	31.6	[26]
7	*Lepus coreanus*	KF040450.1	17.471	13.2	25.7	29.4	31.8	[27]
8	*Lepus europaeus*	AJ421471	17.734	13.3	25.4	29.8	31.5	[28, 29]
9	*Lepus europaeus*	KY211025.1	16.680	13.6	25.3	29.6	31.5	[30]
10	*Lepus granatensis*	KJ397610.1	16.915	13.3	25.8	29.2	31.6	[31]
11	*Lepus hainanus*	JQ219662.1	16.646	13.4	26.1	29.0	31.6	[32]
12	*Lepus sinensis*	KM362831.1	17.438	13.4	26.0	29.1	31.5	[33]
13	*Lepus timidus*	KR030072.1	17.749	13.0	25.7	29.4	31.8	[34]
14	*Lepus tolai*	KM609214	17.472	13.2	25.7	29.5	31.6	[35]
15	*Lepus tolai*	MN539744.1	17.047	13.1	25.7	29.4	31.8	[36]
16	*Lepus yarkadensis*	MN539747	16.808	13.3	25.7	29.5	31.5	[37]
17	*Lepus yarkadensis*	MN450151.1	17.011	13.2	25.6	29.6	31.6	[38]
18	*Cavia porcellus*	MT017565	16.803	14.6	24.7	28.6	32.1	[39]
19	*Ochotona princeps*	NC_005358	16.481	13.8	29.2	25.9	31.1	[40]
20	*Brachylagus idahoensis*	OL436257	17.021	13.6	27.1	28.5	30.8	[41]

### Variation in the PCGs of the Indonesian local rabbit

The total length of 13 protein-coding genes (PCGs) in this study was 11.323 bp, from the shortest to the longest sequence (bp), and amino acid (aa) lengths were as follows: ATP8 (204 bp, 67 aa), ND4L (297 bp, 98 aa), ND3 (357 bp, 118 aa), ND6 (525 bp, 174 aa), ATP6 (681 bp, 226 aa), COX2 (684 bp, 227 aa), COX3 (804 bp, 267 aa), ND1 (957 bp, 318 aa), ND2 (1044 bp, 347 aa), CYTB (1140 bp, 379 aa), COX1 (1542 bp, 513 aa), ND5 (1812 bp, 603 aa), and ND4 (1378 bp, 459 aa). Nine of the 13 PCGs begin with the start codon ATG, while the rest start with the start codons ATA (ND3) and ATT (ND2, ND5, ND4). The stop codons in the Indonesian local rabbit sequence were dominated by TAG (ND3, COX3, COX2, COX1, ND1, and ND5), while the rest were TAA (ND4L, ATP6, ATP8, and ND2), AGG (CYTB and ND6), and TAT (ND4).

The Indonesian local rabbit (*Oryctolagus cuniculus*) in this study showed variation in most of the PCGs when they were aligned with all references (Supplementary data 1). Based on the alignment of the ND1 gene sequences, the Indonesian local rabbit had a longer ND1 sequence (957 bp) than all references (955 bp). There were insertions of 2 bp nucleotides which were A and G, in the end section of the ND1 gene. In addition, when the ND1 gene sequence of the Indonesian local rabbit was aligned with all references, a point mutation transition was found at site 456^th^ (C➔T). However, this mutation was silent and did not alter the amino acid sequence. The ND3 gene sequence of the Indonesian local rabbit had length 357 bp. At the same time, when aligned with all references, there were a silent transition mutation at site 9^th^ (A➔G) and the insertion of a 10 bp nucleotide (TGATAATTAG) in the end section of the ND3 gene.

The alignment results of the ND4L gene sequence showed that the Indonesian local rabbit had the same length compared to all references (297 bp). Nevertheless, when the Indonesian local rabbit ND4L gene sequence was aligned with all references, a transitional point mutation was found at site 162^nd^ (G➔A) in the Indonesian local rabbit as a silent mutation. Similar to the ND4L gene results, the ND5 gene of the Indonesian local rabbit showed the same length in all references (1378 bp). However, when it came to alignment, there were several point mutations at certain sites, namely 187^th^ (C➔G), 321^st^ (C➔T), 891^st^ (T➔C). The point mutation at site 187^th^ (C➔G) was a transversion mutation, which was interpreted as a missense mutation and was located in the first codon. This resulted in different amino acid sequences between leucine (CTA) in the Indonesian local rabbit and valine (GTA) in all references. Mutations at sites 321^st^ (C➔T) and 891^st^ (T➔C) were transition mutations identified as silent mutations. Moreover, ND6, a missense point mutation, was found at site 16^th^ (G➔A) when the Indonesian local rabbit was aligned with all references. This mutation in the first codon caused a difference in the Indonesian local rabbit with amino acid aspartic acid (GAT), compared to all references with asparagine (AAT).

The alignment results of the COX1 gene sequence of the Indonesian local rabbit showed the same length in all references (1542 bp) but varied at specific sites. These variations were found at site 1017^th^ (A➔C) as a transversion mutation and at 1479 (G➔A) as a transition mutation; however, both mutations were silent. The sequence of the COX2 gene of the Indonesian local rabbit was 684 bp in length, which is similar to all references. However, there was a silent point mutation at site 207^th^ (G➔A). Furthermore, an insertion was found in the end section of the COX3 gene in the Indonesian local rabbit that was 20 bp long with the sequence ACTCTTTTAGTATCAACTAG in the end section of the COX3 gene. Thus, the COX3 gene in the Indonesian local rabbit is 804 bp in length.

Further variations were also observed in ATP6. This study showed a 681 bp in length of ATP6 gene with the insertion of a 1 bp nucleotide at the end section of the ATP6 gene in an Indonesian local rabbit in the form of adenine. Conversely, the ATP8 gene of the Indonesian local rabbit showed no variation when aligned with all references, and it also had the same length (204 bp). The CYTB gene of the Indonesian local rabbit in this study was 1140 bp in length. When the CYTB gene sequence of the Indonesian local rabbit was aligned with all the references, there was a point mutation at site 571^st^ (G➔A), which was interpreted as a missense mutation. This mutation in the first codon caused a difference in the amino acid sequence; the Indonesian local rabbit showed alanine (GCT), whereas all references showed threonine (ACT).

### Variation in rRNA, tRNA and the D-loop of the Indonesian local rabbit

This study showed that the Indonesian local rabbit had a 12S rRNA sequence 957 bp in length and was between trnF and trnV. Furthermore, the alignment results between the Indonesian local rabbit and all references of the 12S RNA sequence showed a point mutation at site 587^th^ (T➔C). Furthermore, sequencing results showed that the 16S rRNA sequence of the Indonesian local rabbit was 1581 bp, flanked by trnV and trnL. Moreover, the alignment results showed that there was variation at site 1110^th^ in the 16S rRNA sequence caused by the transition mutation. This was C in an Indonesian local rabbit, whereas all references showed T. The total length of the 22 tRNA sequences of the Indonesian local rabbits was 1505 bp, with an average length of 68 bp. All tRNAs were distributed among PCGs, rRNA, and D_loop regions, with length ranging from 59 bp (trnS) to 75 bp (trnL). The D-loop sequence of the Indonesian local rabbit was located between trnF (gaa) and trnP (ugg). The Indonesian local rabbit D-loop sequence was 2.023 bp long. It were consisting of the specific sequence along 20 bp in length that was repeated 11 times and the specific sequence along 151 bp in length that was repeated five times.

### Phylogenetic study

**Fig. 3:**
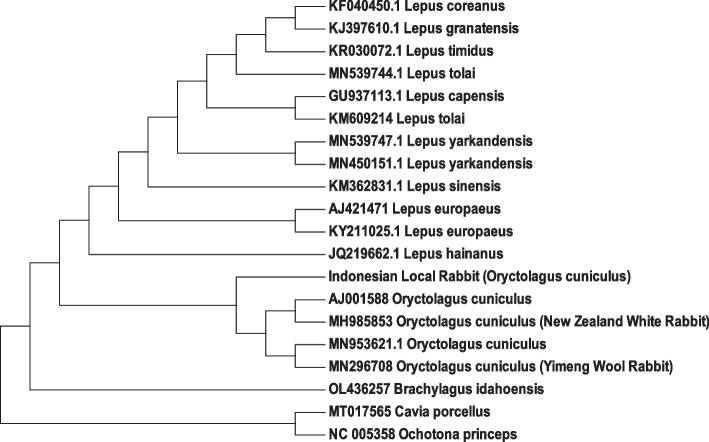
Complete sequences of mitochondrial genomes of 9 *Lepus* species were used to construct phylogenetic tree in comparison to local Indonesian rabbit.The 9 species of Lepus are Lepus coreanus, Lepus granatensis, Lepus timidus, Lepus tolai, Lepus capensis Lepus tolai Lepus yarkendensis, Lepus yarkendensis, Lepus sinensis Lepus eurapaeous, Lepus hainanus. While Cavia porcellus and Ochotona princeps are using as outgroup

A phylogenetic tree was constructed based on the comparison of complete mitochondrial genome sequences of the Indonesian local rabbit (*Oryctolagus cuniculus*) and other 9 species of Leporidae family by the Maximum Likelihood method. Where bootstrap was set at 1000 for phylogeny test (Fig. 3). As one of *Oryctolagus cuniculus* sub-species, the Indonesian local rabbit in this study were in the same main clade with other *Oryctolagus cuniculus* (NZW, YW, CR) and close to *Lepus sp.* and *Brachylagus sp*. While *Cavia sp.* and *Ochotona sp.* were in different main clade.

## Discussion

Result showed that the mtDNA genome of Indonesian local rabbit (17.469 bp) in this study was longer than that of CR rabbits (17.174 bp), YW rabbits (16.740 bp), and fractionally longer than that of New Zealand White rabbits (17.418 bp) [17–19]. Result in this study showed that the positions of trnS, trnY, trnC, trnN, trnA, trnQ, trnE, and ND6 are in the heavy strand (H), while the rest are in the light strand (L). These results was unique to those reported for YW rabbits by Yao et al. [19] and CR rabbits by Wang et al. [17], in which all protein-coding genes were found in the heavy strand, except for ND6, which was present in the light strand. Furthermore, both rabbits showed that tRNA, Gln, Ala, Asn, Cys, Tyr, Ser, Glu, and Pro were found on the light strand, whereas the other 14 tRNAs were found on the heavy strand. Other specific differences were also found in the percentage of nucleotides in the mtDNA genome of *Oryctolagus cuniculus*. The Indonesian local rabbit *Oryctolagus cuniculus* had a two-fold higher percentage of G than the average of other rabbits and outgroups (26% vs. ~ 13%), and the percentage of T was 2–3% higher than that of other rabbits and outgroups. However, the percentage of C was two-fold less than that of other rabbits and outgroups (26% vs. 13%), and the percentage of T was 2–3% lower than that in other rabbits and outgroups.

The difference result of the stop and start codons in PCGs of mtDNA genome of YW rabbits was reported by Yao et al. [19] that the ten protein-coding genes utilize the start codon ATG, except for ND2 and ND5, which use the start codon ATT, and ND3, which uses the start codon ATA. Regarding stop codons in the YW rabbit mtDNA genome, ND2 and ND4L end with TAA, COX2, and ND5 end with TAG, and ND6 ends with AGG. Eight genes also had an incomplete stop codon T– termination, which was the 5′ terminal of the gene next to them. They presumably created a complete stop codon via post-transcriptional polyadenylation, as reported by Anderson et al. [42]. Other previous findings showed that CR rabbits had the same start codon for PCGs as YW rabbits, excluding ND6, which had the start codon CCT, whereas for the stop codon, CR rabbits had TAT for ND1, TAA for ND2, ATP8, ATP6, and ND4L; TAG for COX1, COX2, and ND5; TCT for ND4; CAT for COX3 and ND6; ATA for ND3; and AGG for CYTB [17].

When the ND1 gene sequence of the Indonesian local rabbit was aligned with only CR rabbits [17], and variation were found at sites 316^th^ (C➔T) and 791^st^ (T➔C). The mutation at site 316^th^ (C➔T) was a silent mutation, and the mutation at site 791^st^ (T➔C) was a missense mutation located in the second codon. Proline (CCC) was detected in the CR rabbits and leucine (CTC) in The Indonesian rabbits. The ND3 gene sequence of the Indonesian local rabbit had the same length as the NZW2 rabbit ND3 gene sequence, which was 357 bp [18]. While the other references were only 347 bp long [16, 17, 19]. The insertion of a 10 bp nucleotide (TGATAATTAG) in the end section of the ND3 gene of the rabbit in this study indicated that Indonesian local rabbits had a longer amino acid sequence with the addition of tyrosine, aspartic acid, and asparagine.

The ND4L and ND5 gene sequence had the same length with all references [16–19] and found some mutations in certain sites that comprising a silent mutation in ND4L; 2 silent mutations and a missense mutation in ND5. Other than the three point mutations in ND5, there was also a point mutation found at site 1102^nd^ (T➔C) as a transition mutation, which was also indicated as a silent mutation. However, this mutation was found when the Indonesian local rabbit was aligned with the CR rabbits [17]. Furthermore, two transition mutations were found in ND2 at sites 220^th^ and 267^th^ as A➔G when the Indonesian rabbits in this study were aligned with CR rabbits [17]. These mutations at site 220^th^ of the first codon caused a difference in the amino acid sequence between isoleucine (ATT) in the Indonesian local rabbit and valine (GTT) in CR rabbits, while the mutation at site 267^th^ was a silent mutation. In contrast, a mutation in ND4 was found at site 1370^th^ (C➔T) only when the Indonesian local rabbit was aligned with the NZW1 and NZW2 rabbits [16, 18]. These mutations caused a difference in amino acids, such as proline (CCA) in Indonesian rabbits and leucine (CTA) in NZW1 and NZW2 rabbits. In contrast, when the Indonesian local rabbit ND4 gene sequence was aligned with the YW and CR rabbit ND4 gene sequences, no variation (monomorphic) was found [17, 19]. Based on Hoefs et al. [43], all ND genes including ND1, ND2, ND3, ND4, ND4L, ND5 and ND6 gene provided instructions for making a protein called NADH dehydrogenase. This protein is part of a large enzyme complex known as complex I. It is one of several enzyme complexes necessary for oxidative phosphorylation (OXPHOS) that carries out chemical reactions that drive the production of ATP. Complex I is also responsible for the first step of the electron transport process [44].

Moreover, when COX1 gene sequence of the Indonesian local rabbit was aligned with only CR rabbits [17], three point mutations were identified as transition mutations. These were located at site 569^th^ (T➔C) and 1463^rd^ (C➔T) in the second codon and 1252^nd^ (T➔C) in the first codon, all of which were missense mutations. There were point mutations causing differences between isoleucine (ATC), phenylalanine (TTT), and threonine (ACA) in the Indonesian local rabbit, and threonine (ACC), leucine (CTT), and isoleucine (ATA) in the CR rabbit at sites 569^th^, 1252^nd^, and 1463^rd^, respectively. As another comparison between the Indonesian local rabbit with NZW1 rabbits and NZW2 rabbits [16, 18], a point mutation was found in the site 517^th^ (C➔G) as transversion mutation that interpreted as a missense mutation. This mutation in the first codon led to differences in the amino acid proline (CCC) in the Indonesian local rabbit and alanine (GCC) in the NZW1 and NZW2 rabbits. At the same site, YW and CR rabbits showed no differences [17, 19]. Differently, the point mutation of COX2 gene sequence was at site 640^th^ (G➔A) only when the Indonesian local rabbit was aligned with CR rabbits [17]. These were missense mutations and were present in the first codon, which caused a difference in amino acids at the site between valine (GTT) in the Indonesian local rabbit and isoleucine (ATT) in CR rabbits. According to Kadenbach [45], COX encodes a cytochrome oxidase c enzyme known as complex IV. It is the last enzyme in the respiratory electron transport chain of cells and is located in the membrane. COX plays a key role in the electron transport chain and catalyses the reduction of molecular oxygen to water. In addition, COX is responsible for the last step of OXPHOS before ATP generation [46].

ATP6 gene sequence in this study showed a 681 bp in length and was the same as that in NZW2 rabbits and CR rabbits [17, 18]. Other studies (NZW1 and YW rabbits) showed a 680 bp in length of ATP6 gene [16, 19]. Additionally, Anderson et al. [42], ATP genes play a role in encoding the ATP synthase enzyme, which is also known as complex V. This enzyme was responsible for the final OXPHOS step. More precisely, one component of ATP synthase permits the passage of positively charged particles (protons) through a unique membrane inside the mitochondria. The energy generated by this proton is used by another portion of the enzyme to convert adenosine diphosphate (ADP) into ATP.

CYTB codes for the production of cytochrome b protein. This protein is one of 11 components of a group of proteins called complex III. It performs one step in the OXPHOS process, in which oxygen and simple sugars are used to create adenosine triphosphate (ATP) as the cell's main energy source [47]. The CYTB gene of the Indonesian local rabbit in this study was the same length as NZW2, YW, and CR rabbits (1140 bp) [17–19], but longer than that of NZW1 rabbits [16] that had a 1 bp deletion in the end section of the CYTB gene. In addition, there was also a point mutation at sites 641^st^ (A➔G) and 1103^rd^ (T➔C) as missense mutations; however, these mutations were only found when the CYTB gene of the Indonesian local rabbit was aligned with CR rabbits [17]. Mutation at the site 641^st^ (A➔G) and 1103^rd^ (T➔C) in the second codon was causing a difference in amino acid sequence, where the Indonesian local rabbits showed Asparagine (AAC) and Leucine (CTC), whereas CR rabbits showed Serine (AGC) and Proline (CCC), respectively.

According to Yang et al. [48], ribosomal RNAs which are 12S rRNA and 16S rRNA that encoded by the mitochondrial genome are required to translate mRNA into mitochondrial proteins. Both rRNA which make approximately 1/16 for 12S rRNA and 1/10 for 16S rRNA of the whole mitochondrial genome, have many nucleotide changes. This study showed that the Indonesian local rabbit had a 12S rRNA sequence 957 bp in length and was between trnF and trnV. This is similar to the 12S rRNA sequences of NZW1 and CR rabbits [16, 17]. NZW2 and YW rabbits were reported to have 12S rRNA along 938 bp and 956 bp, respectively [18, 19]. Moreover, some point mutations were also found at sites 162^nd^ (A➔G), 726^th^ (T➔A), 771^st^ (A➔T), and 791^st^ (T➔C), but these mutations were present only when the Indonesian local rabbit was aligned with CR rabbits [17]. On the other hand, the length difference of the 12S rRNA sequence between the Indonesian local rabbit and NZW2 rabbit was due to a deletion of 19 bp in the middle region. At the same time, a 1 bp in length deletion was found in the end region of the 12S rRNA of YW rabbits.

Furthermore, sequencing results showed that the 16S rRNA sequence of the Indonesian local rabbit was 1581 bp, flanked by trnV and trnL. Prior researchers have reported results different from those of this study. NZW1 and CR rabbits were 1579 bp in length [16, 17]. The difference in the 16S rRNA sequence of both rabbits with the Indonesian local rabbit was due to the deletion of 2 bp in the beginning region of the 16S rRNA of NZW1 rabbits and CR rabbits. Other researchers reported that the YW rabbit had 1575 bp of 16S rRNA and it had a 6 bp in length deletion in the beginning region when compared to The Indonesian local rabbit 16S rRNA sequence. In contrast, Hu et al. [18] reported a shorter 16S rRNA sequence (1561 bp) in NZW2 rabbits than in this study and other studies. Deletion of 2 bp in the beginning region and 18 bp in the middle region of the 16S rRNA of NZW2 rabbits led to a length difference in this study. In addition, when 16S rRNA gene sequence of the Indonesian local rabbit was aligned only with the CR rabbits, the results showed more variation at sites 61^st^ (T➔C), 636^th^ (A➔G), and 982^nd^ (A➔G).

tRNAs are mitochondrial components essential for the maturation of RNA into polycistronic transcripts. They decode an RNA molecule that aids in translating messenger RNA (mRNA) sequences into proteins [49]. These genes are frequently located between protein-coding and rRNA genes. In addition, based on Mauro et al. [50], vertebrate mitogenome organization is extremely conserved, and tRNA genes are involved in rearrangements in closely related taxa. Results of tRNAs in this study were showed similar to those obtained for NZW1 rabbit and CR rabbit tRNA sequences [16, 17]. In contrast, Yao et al. [19] reported that the lengths of 22 tRNA genes in YW rabbits ranged from 60 to 74 bp.

Result of D-loop gene sequence in this study was highly different from NZW1 rabbits, particularly in terms of sequence length and character [16]. The Indonesian local rabbit D-loop sequence was 2.023 bp long, whereas NZW1 rabbits were 1.800 bp long. This difference was due to the presence of two specific sequences. First, the specific sequence along 20 bp in length was repeated 11 times in the Indonesian local rabbit D-loop, whereas in NZW1, the rabbit was only repeated eight times. Second, the specific sequence of 151 bp was repeated five times, whereas in NZW1, the rabbit was only repeated four times. According to the aligned sequences between the Indonesian local rabbit and NZW1 rabbit, there were 69 bp nucleotide insertions along the D-loop region of the Indonesian local rabbit. In addition, transitional mutations were also found at some points, where NZW1 rabbits showed cytosine, whereas Indonesian rabbits showed thymine (C > T).

Furthermore, another study reported that the D-loop sequence lengths were 2.010 bp in NZW2 rabbits, which showed nine repetitions of 20 bp of repetition sequence and three repetitions of 151 bp of repetition sequence [18]; 1.743 bp in CR rabbits, which showed five repetitions of 20 bp of repetition sequence and three repetitions of 151 bp of repetition sequence [17]; and 1.294 bp in YW rabbits, which showed three repetitions of 20 bp of repetition sequence and were only shown for one repetition sequence of 151 bp [19]. These differences were the reason for the D-loop sequence of the Indonesian local rabbit, which had the most extended sequence among the other references from GenBank. These circumstances also affected the longer mtDNA genome sequences of the Indonesian local rabbit in this study compared to other GenBank references. These facts are the novelty of this MtDNA genome study of the Indonesian local rabbit, which has not been reported before.

The phylogenic tree described that within sub-species *Oryctolagus cuniculus*, the Indonesian local rabbit was in different branch and separated with NZW, YW and CR rabbit breed. The different among breed was in line with the morphologically distinctive among these rabbits. Indonesian local rabbits have short, coarse fur that is either black, white, agouty, or any mix of those colors. They have medium-sized of body with a maximum mature weight is only 2.5 kg [51]. NZW and CR are the rabbits with commercial body types. This body type is medium-sized, with a body depth that is uniformly equal to their body breadth. When viewed from above, there will be a little taper from the shoulder to the hips. A firmness of flesh and fullness of body are especially crucial for commercial-type rabbits because they are primarily kept for their meat. The adult body weight of both two breeds reached 5 kg and 3.6 kg for NZW and CR, respectively [52, 53]. The color of New Zealand rabbit was white, red, black and broken, while the rex has a broader variety of colors. In the other hand YW is native rabbit in Shandong Province of China. YW is a unique commercial animal with a large market potential due to its increased wool yield and better wool quality, which increases its economic worth [54].

This difference of branch was an evidence and a consequence of the unique specific sequences possessed by only Indonesian local rabbits in this study. The study reported phylogeny of rabbits from complete mtDNA genome was limited. The specific genes have been used for phylogeny studies were Cytochrome b, 12SrNA, 16S rRNA MGF, PRKCI, SPTBN1, THY, and TG [55–57]. They reported that *Oryctolagus cuniculus* known as European rabbit was separated but closely from *Pentalagus, Caprolagus* and *Bunolagus*. Based on geographical cluster, *Pentalagus, Caprolagus, Bunolagus*, and *Oryctolagus* were originated from South Asia, India, South Africa and Europe, respectively. In the level of subspecies, the study in Egypt reported that Baladi Black, California, Chinchilla, Flander, New Zealand white and Rex were grouped as *Oryctolagus cuniculus* [58]. Based on the result, Indonesian local Rabbits strongly suspected brought from Europe during the colonial period in Indonesia.

## Conclusions

mtDNA genome of Indonesian local rabbit was the longest and had the most extended D-loop sequence among the other references of *Oryctolagus cuniculus*. Other specific differences were also found in the percentage of nucleotides and variation in most of the PCGs when they were aligned with *Oryctolagus cuniculus* references from GenBank. According to phylogeny analysis Indonesian local Rabbits strongly suspected brought from Europe during the colonial period in Indonesia.

### Supplementary Information

**Additional file 1. **Genetic characteristics of complete mtDNA genome sequence of Indonesian Local rabbit (Oryctolagus cuniculus) (2023).

## Data Availability

All data are primary data and generated from our original research. All research materials belong to our laboratory in Genetic, Breeding and Reproduction Laboratory, Department of Animal Science, Faculty of Animal and Agricultural Sciences, Universitas Diponegoro.

## Abbreviations

A: Adenine
aa: Amino acid
ATP: Adenine triphosphate
bp: Base pair
C: Cytosine
COX: Cytochrome oxidase
CR: Chuanbai Rex
CYTB: Cytochrome b
D-loop: Displacement loop
DNA: Deoxyribonucleic acid
G: Guanine
MEGA: Molecular evolutionary genetic analysis
mtDNA: Mitochondrial DNA
ND: NADH dehydrogenase
NZW: New Zealand White
PCGs: Protein coding gene
RNA: Ribonucleic acid
rRNA: Ribosomal RNA
T: Tymine
tRNA: Transfer RNA
trnA: TRNA alanine
trnC: TRNA cysteine
trnD: TRNA aspartic acid
trnE: TRNA glutamic acid
trnF: TRNA phenylalanine
trnG: TRNA glycine
trnH: TRNA histidine
trnK: TRNA lysine
trnl: TRNA isoleusine
trnL: TRNA leucine
trnM: TRNA methionine
trnN: TRNA asparagine
trnP: TRNA proline
trnQ: TRNA glutamine
trnR: TRNA arginine
trnS: TRNA serine
trnT: TRNA threonine
trnV: TRNA valine
trnW: TRNA tryptophan
trnY: TRNA tyrosine
WGS: Whole genome sequencing
YW: Yimeng Wool
