# Supplementary material for: Genetic characteristics of complete mtDNA genome sequence of Indonesian local rabbit (Oryctolagus cuniculus)
Source: J Genet Eng Biotechnol. 2023 Oct 9;21:96. doi: 10.1186/s43141-023-00546-1 (PMC10562326; doi:10.1186/s43141-023-00546-1)
Supplement: Supplementary file 1 — Additional file 1. Genetic characteristics of complete mtDNA genome sequence of Indonesian Local rabbit (Oryctolagus cuniculus) (2023). [file 43141_2023_546_MOESM1_ESM.pdf]

Genetic characteristics of complete mtDNA genome sequence of Indonesian Local rabbit (*Oryctolagus cuniculus*) (2023)

by: A. Setiaji, D. A. Lestari, N. S. Pandupuspitasari, I. Agusetyaningsih, F. A. Khan

```
#MEGA
ND1 rabbit alignment

[                                           111 111 111 122 222 222 223 333 333 333 444 444 444 455 555 555 556 666 666 666 777 777 777 ]
[                               123 456 789 012 345 678 901 234 567 890 123 456 789 012 345 678 901 234 567 890 123 456 789 012 345 678 ]
#Indonesian_Local_rabbit_ND1    ATG TTC CTA ATT AAT ACA CTC CTT TTA ATC CTA CCT GTA CTT TTA GCC ATA GCA TTC CTC ACC TTA GTC GAA CGA AAA
#MH985853.1_NZW2_ND1            ... ... ... ... ... ... ... ... ... ... ... ... ... ... ... ... ... ... ... ... ... ... ... ... ... ...
#MN296708_YW_ND1                ... ... ... ... ... ... ... ... ... ... ... ... ... ... ... ... ... ... ... ... ... ... ... ... ... ...
#AJ001588.1_NZW1_ND1            ... ... ... ... ... ... ... ... ... ... ... ... ... ... ... ... ... ... ... ... ... ... ... ... ... ...
#MN953621_CR_ND1                ... ... ... ... ... ... ... ... ... ... ... ... ... ... ... ... ... ... ... ... ... ... ... ... ... ...

[                                                           111 111 111 111 111 111 111 111 111 111 111 111 111 111 111 111 111 111 111 ]
[                               788 888 888 889 999 999 999 000 000 000 011 111 111 112 222 222 222 333 333 333 344 444 444 445 555 555 ]
[                               901 234 567 890 123 456 789 012 345 678 901 234 567 890 123 456 789 012 345 678 901 234 567 890 123 456 ]
#Indonesian_Local_rabbit_ND1    ATC TTA GGG TAC ATA CAA CTA CGT AAA GGC CCA AAC ATT GTA GGA CCC TAT GGC CTC CTC CAA CCA ATC GCA GAC GCT
#MH985853.1_NZW2_ND1            ... ... ... ... ... ... ... ... ... ... ... ... ... ... ... ... ... ... ... ... ... ... ... ... ... ...
#MN296708_YW_ND1                ... ... ... ... ... ... ... ... ... ... ... ... ... ... ... ... ... ... ... ... ... ... ... ... ... ...
#AJ001588.1_NZW1_ND1            ... ... ... ... ... ... ... ... ... ... ... ... ... ... ... ... ... ... ... ... ... ... ... ... ... ...
#MN953621_CR_ND1                ... ... ... ... ... ... ... ... ... ... ... ... ... ... ... ... ... ... ... ... ... ... ... ... ... ...

[                               111 111 111 111 111 111 111 111 111 111 111 111 111 111 122 222 222 222 222 222 222 222 222 222 222 222 ]
[                               555 666 666 666 677 777 777 778 888 888 888 999 999 999 900 000 000 001 111 111 111 222 222 222 233 333 ]
[                               789 012 345 678 901 234 567 890 123 456 789 012 345 678 901 234 567 890 123 456 789 012 345 678 901 234 ]
#Indonesian_Local_rabbit_ND1    ATT AAG CTA TTC ACT AAA GAA CCC CTA CGA CCC CTA ACA TCC TCT CCG CTA CTC TTT ATT ATC GCC CCA ACC CTA GCT
#MH985853.1_NZW2_ND1            ... ... ... ... ... ... ... ... ... ... ... ... ... ... ... ... ... ... ... ... ... ... ... ... ... ...
#MN296708_YW_ND1                ... ... ... ... ... ... ... ... ... ... ... ... ... ... ... ... ... ... ... ... ... ... ... ... ... ...
#AJ001588.1_NZW1_ND1            ... ... ... ... ... ... ... ... ... ... ... ... ... ... ... ... ... ... ... ... ... ... ... ... ... ...
#MN953621_CR_ND1                ... ... ... ... ... ... ... ... ... ... ... ... ... ... ... ... ... ... ... ... ... ... ... ... ... ...

[                               222 222 222 222 222 222 222 222 222 222 222 222 222 222 222 222 222 222 222 222 222 223 333 333 333 333 ]
[                               333 334 444 444 444 555 555 555 566 666 666 667 777 777 777 888 888 888 899 999 999 990 000 000 000 111 ]
[                               567 890 123 456 789 012 345 678 901 234 567 890 123 456 789 012 345 678 901 234 567 890 123 456 789 012 ]
#Indonesian_Local_rabbit_ND1    CTA ACT CTC GCA CTA TCA ATG TGA CTT CCT ATC CCC ATA CCT TAC CCA CTA GTC AAC CTA AAT ATA GGC ATT CTA TTC
#MH985853.1_NZW2_ND1            ... ... ... ... ... ... ... ... ... ... ... ... ... ... ... ... ... ... ... ... ... ... ... ... ... ...
#MN296708_YW_ND1                ... ... ... ... ... ... ... ... ... ... ... ... ... ... ... ... ... ... ... ... ... ... ... ... ... ...
#AJ001588.1_NZW1_ND1            ... ... ... ... ... ... ... ... ... ... ... ... ... ... ... ... ... ... ... ... ... ... ... ... ... ...
#MN953621_CR_ND1                ... ... ... ... ... ... ... ... ... ... ... ... ... ... ... ... ... ... ... ... ... ... ... ... ... ...
```

Genetic characteristics of complete mtDNA genome sequence of Indonesian Local rabbit (*Oryctolagus cuniculus*) (2023)
by: A. Setiaji, D. A. Lestari, N. S. Pandupuspitasari, I. Agusetyaningsih, F. A. Khan

```
[                               333 333 333 333 333 333 333 333 333 333 333 333 333 333 333 333 333 333 333 333 333 333 333 333 333 333 ]
[                               111 111 122 222 222 223 333 333 333 444 444 444 455 555 555 556 666 666 666 777 777 777 788 888 888 889 ]
[                               345 678 901 234 567 890 123 456 789 012 345 678 901 234 567 890 123 456 789 012 345 678 901 234 567 890 ]
#Indonesian_Local_rabbit_ND1    ATC CTA GCA ACC TCC AGC TTA GCC GTT TAC TCA ATC CTA TGA TCA GGA TGA GCA TCC AAC TCA AAA TAT GCC CTA TTT
#MH985853.1_NZW2_ND1            ... ... ... ... ... ... ... ... ... ... ... ... ... ... ... ... ... ... ... ... ... ... ... ... ... ...
#MN296708_YW_ND1                ... ... ... ... ... ... ... ... ... ... ... ... ... ... ... ... ... ... ... ... ... ... ... ... ... ...
#AJ001588.1_NZW1_ND1            ... ... ... ... ... ... ... ... ... ... ... ... ... ... ... ... ... ... ... ... ... ... ... ... ... ...
#MN953621_CR_ND1                ... T.. ... ... ... ... ... ... ... ... ... ... ... ... ... ... ... ... ... ... ... ... ... ... ... ...

[                               333 333 333 444 444 444 444 444 444 444 444 444 444 444 444 444 444 444 444 444 444 444 444 444 444 444 ]
[                               999 999 999 000 000 000 011 111 111 112 222 222 222 333 333 333 344 444 444 445 555 555 555 666 666 666 ]
[                               123 456 789 012 345 678 901 234 567 890 123 456 789 012 345 678 901 234 567 890 123 456 789 012 345 678 ]
#Indonesian_Local_rabbit_ND1    GGT GCT CTC CGA GCA GTC GCA CAA ACC ATT TCT TAC GAA GTC ACA CTT GCA ATT ATC CTC CTA TGT ATC CTC CTA ATA
#MH985853.1_NZW2_ND1            ... ... ... ... ... ... ... ... ... ... ... ... ... ... ... ... ... ... ... ... ... ..C ... ... ... ...
#MN296708_YW_ND1                ... ... ... ... ... ... ... ... ... ... ... ... ... ... ... ... ... ... ... ... ... ..C ... ... ... ...
#AJ001588.1_NZW1_ND1            ... ... ... ... ... ... ... ... ... ... ... ... ... ... ... ... ... ... ... ... ... ..C ... ... ... ...
#MN953621_CR_ND1                ... ... ... ... ... ... ... ... ... ... ... ... ... ... ... ... ... ... ... ... ... ..C ... ... ... ...

[                               444 444 444 444 444 444 444 444 444 444 455 555 555 555 555 555 555 555 555 555 555 555 555 555 555 555 ]
[                               677 777 777 778 888 888 888 999 999 999 900 000 000 001 111 111 111 222 222 222 233 333 333 334 444 444 ]
[                               901 234 567 890 123 456 789 012 345 678 901 234 567 890 123 456 789 012 345 678 901 234 567 890 123 456 ]
#Indonesian_Local_rabbit_ND1    AAT GGC TCA TTT ACA TTA TCA TCC CTA ATC ACA ACA CAA GAA TAT ATA TGA ATC CTA CTA CCA GCA TGA CCA CTT GCC
#MH985853.1_NZW2_ND1            ... ... ... ... ... ... ... ... ... ... ... ... ... ... ... ... ... ... ... ... ... ... ... ... ... ...
#MN296708_YW_ND1                ... ... ... ... ... ... ... ... ... ... ... ... ... ... ... ... ... ... ... ... ... ... ... ... ... ...
#AJ001588.1_NZW1_ND1            ... ... ... ... ... ... ... ... ... ... ... ... ... ... ... ... ... ... ... ... ... ... ... ... ... ...
#MN953621_CR_ND1                ... ... ... ... ... ... ... ... ... ... ... ... ... ... ... ... ... ... ... ... ... ... ... ... ... ...

[                               555 555 555 555 555 555 555 555 555 555 555 555 555 555 555 555 555 556 666 666 666 666 666 666 666 666 ]
[                               444 555 555 555 566 666 666 667 777 777 777 888 888 888 899 999 999 990 000 000 000 111 111 111 122 222 ]
[                               789 012 345 678 901 234 567 890 123 456 789 012 345 678 901 234 567 890 123 456 789 012 345 678 901 234 ]
#Indonesian_Local_rabbit_ND1    ATA ATA TGA TTT ATC TCG ACC CTA GCA GAA ACC AAC CGG GCT CCA TTT GAC TTA ACC GAA GGT GAA TCA GAG CTC GTC
#MH985853.1_NZW2_ND1            ... ... ... ... ... ... ... ... ... ... ... ... ... ... ... ... ... ... ... ... ... ... ... ... ... ...
#MN296708_YW_ND1                ... ... ... ... ... ... ... ... ... ... ... ... ... ... ... ... ... ... ... ... ... ... ... ... ... ...
#AJ001588.1_NZW1_ND1            ... ... ... ... ... ... ... ... ... ... ... ... ... ... ... ... ... ... ... ... ... ... ... ... ... ...
#MN953621_CR_ND1                ... ... ... ... ... ... ... ... ... ... ... ... ... ... ... ... ... ... ... ... ... ... ... ... ... ...
```

```
[                               666 666 666 666 666 666 666 666 666 666 666 666 666 666 666 666 666 666 666 666 666 666 666 666 666 777 ]
[                               222 223 333 333 333 444 444 444 455 555 555 556 666 666 666 777 777 777 788 888 888 889 999 999 999 000 ]
[                               567 890 123 456 789 012 345 678 901 234 567 890 123 456 789 012 345 678 901 234 567 890 123 456 789 012 ]
#Indonesian_Local_rabbit_ND1    TCC GGC TTC AAT GTG GAG TAC GCA GGA GGC CCC TTT GCT CTT TTC TTT TTA GCT GAA TAT ACT AAT ATT ATC CTT ATA
#MH985853.1_NZW2_ND1            ... ... ... ... ... ... ... ... ... ... ... ... ... ... ... ... ... ... ... ... ... ... ... ... ... ...
#MN296708_YW_ND1                ... ... ... ... ... ... ... ... ... ... ... ... ... ... ... ... ... ... ... ... ... ... ... ... ... ...
#AJ001588.1_NZW1_ND1            ... ... ... ... ... ... ... ... ... ... ... ... ... ... ... ... ... ... ... ... ... ... ... ... ... ...
#MN953621_CR_ND1                ... ... ... ... ... ... ... ... ... ... ... ... ... ... ... ... ... ... ... ... ... ... ... ... ... ...

[                               777 777 777 777 777 777 777 777 777 777 777 777 777 777 777 777 777 777 777 777 777 777 777 777 777 777 ]
[                               000 000 011 111 111 112 222 222 222 333 333 333 344 444 444 445 555 555 555 666 666 666 677 777 777 778 ]
[                               345 678 901 234 567 890 123 456 789 012 345 678 901 234 567 890 123 456 789 012 345 678 901 234 567 890 ]
#Indonesian_Local_rabbit_ND1    AAT GCC CTT ACA GCC ATC CTA TTC CTA GGC TCA TTT CAC AGC CAC ACT AAC CCA GAA ATA TTT ACA GTC AAC TTT GCC
#MH985853.1_NZW2_ND1            ... ... ... ... ... ... ... ... ... ... ... ... ... ... ... ... ... ... ... ... ... ... ... ... ... ...
#MN296708_YW_ND1                ... ... ... ... ... ... ... ... ... ... ... ... ... ... ... ... ... ... ... ... ... ... ... ... ... ...
#AJ001588.1_NZW1_ND1            ... ... ... ... ... ... ... ... ... ... ... ... ... ... ... ... ... ... ... ... ... ... ... ... ... ...
#MN953621_CR_ND1                ... ... ... ... ... ... ... ... ... ... ... ... ... ... ... ... ... ... ... ... ... ... ... ... ... ...

[                               777 777 777 777 777 777 788 888 888 888 888 888 888 888 888 888 888 888 888 888 888 888 888 888 888 888 ]
[                               888 888 888 999 999 999 900 000 000 001 111 111 111 222 222 222 233 333 333 334 444 444 444 555 555 555 ]
[                               123 456 789 012 345 678 901 234 567 890 123 456 789 012 345 678 901 234 567 890 123 456 789 012 345 678 ]
#Indonesian_Local_rabbit_ND1    ACC AAG ACC CTC CTA CTC ACA ATA ACA TTT CTA TGG ATT CGA GCA TCC TAT CCC CGA TTT CGC TAT GAC CAG CTA ATA
#MH985853.1_NZW2_ND1            ... ... ... ... ... ... ... ... ... ... ... ... ... ... ... ... ... ... ... ... ... ... ... ... ... ...
#MN296708_YW_ND1                ... ... ... ... ... ... ... ... ... ... ... ... ... ... ... ... ... ... ... ... ... ... ... ... ... ...
#AJ001588.1_NZW1_ND1            ... ... ... ... ... ... ... ... ... ... ... ... ... ... ... ... ... ... ... ... ... ... ... ... ... ...
#MN953621_CR_ND1                ... ... ... .C. ... ... ... ... ... ... ... ... ... ... ... ... ... ... ... ... ... ... ... ... ... ...

[                               888 888 888 888 888 888 888 888 888 888 888 888 888 889 999 999 999 999 999 999 999 999 999 999 999 999 ]
[                               566 666 666 667 777 777 777 888 888 888 899 999 999 990 000 000 000 111 111 111 122 222 222 223 333 333 ]
[                               901 234 567 890 123 456 789 012 345 678 901 234 567 890 123 456 789 012 345 678 901 234 567 890 123 456 ]
#Indonesian_Local_rabbit_ND1    CAC CTC TTG TGA AAA AGT TTT CTA CCC CTC ACA TTA GCC CTG TGC ATA TGA CAT ATC TCA ATG CCT ATC ATA CTT TCA
#MH985853.1_NZW2_ND1            ... ... ... ... ... ... ... ... ... ... ... ... ... ... ... ... ... ... ... ... ... ... ... ... ... ...
#MN296708_YW_ND1                ... ... ... ... ... ... ... ... ... ... ... ... ... ... ... ... ... ... ... ... ... ... ... ... ... ...
#AJ001588.1_NZW1_ND1            ... ... ... ... ... ... ... ... ... ... ... ... ... ... ... ... ... ... ... ... ... ... ... ... ... ...
#MN953621_CR_ND1                ... ... ... ... ... ... ... ... ... ... ... ... ... ... ... ... ... ... ... ... ... ... ... ... ... ...
```

Genetic characteristics of complete mtDNA genome sequence of Indonesian Local rabbit (*Oryctolagus cuniculus*) (2023)
by: A. Setiaji, D. A. Lestari, N. S. Pandupuspitasari, I. Agusetyaningsih, F. A. Khan

```
[                                999 999 999 999 999 999 999 ]
[                                333 444 444 444 455 555 555 ]
[                                789 012 345 678 901 234 567 ]
#Indonesian_Local_rabbit_ND1     AGT ATC CCT CCT CAG ATA TAG
#MH985853.1_NZW2_ND1             ... ... ... ... ... ... .--
#MN296708_YW_ND1                 ... ... ... ... ... ... .--
#AJ001588.1_NZW1_ND1             ... ... ... ... ... ... .--
#MN953621_CR_ND1                 ... ... ... ... ... ... .—
```

Genetic characteristics of complete mtDNA genome sequence of Indonesian Local rabbit (*Oryctolagus cuniculus*) (2023)
by: A. Setiaji, D. A. Lestari, N. S. Pandupuspitasari, I. Agusetyaningsih, F. A. Khan

```
#MEGA
ND2 rabbit alignment

[                                           111 111 111 122 222 222 223 333 333 333 444 444 444 455 555 555 556 666 666 666 777 777 777 ]
[                               123 456 789 012 345 678 901 234 567 890 123 456 789 012 345 678 901 234 567 890 123 456 789 012 345 678 ]
#Indonesian_Local_rabbit_ND2    ATT AAC CCC CTA ACT CTT ATT ATT ATC ATA TTT ACT CTA TTT ATA GGC ACG ATA ATC ACA GTA TTT AGC TCC CAC TGA
#MH98583.1_NZW2_ND2             ... ... ... ... ... ... ... ... ... ... ... ... ... ... ... ... ... ... ... ... ... ... ... ... ... ...
#MN296708_YW_ND2                ... ... ... ... ... ... ... ... ... ... ... ... ... ... ... ... ... ... ... ... ... ... ... ... ... ...
#MN953621_CR_ND2                ... ... ... ... ... ... ... ... ... ... ... ... ... ... ... ... ... ... ... ... ... ... ... ... ... ...
#AJ001588.1_NZW1_ND2            ... ... ... ... ... ... ... ... ... ... ... ... ... ... ... ... ... ... ... ... ... ... ... ... ... ...

[                                                           111 111 111 111 111 111 111 111 111 111 111 111 111 111 111 111 111 111 111 ]
[                               788 888 888 889 999 999 999 000 000 000 011 111 111 112 222 222 222 333 333 333 344 444 444 445 555 555 ]
[                               901 234 567 890 123 456 789 012 345 678 901 234 567 890 123 456 789 012 345 678 901 234 567 890 123 456 ]
#Indonesian_Local_rabbit_ND2    CTA ACT ATA TGA ATC GGA TTA GAA ATA AAC ATA CTA GCA ATT ATC CCT ATT CTA ATC AAC AAA GCC ACC CCA CGA TCA
#MH98583.1_NZW2_ND2             ... ... ... ... ... ... ... ... ... ... ... ... ... ... ... ... ... ... ... ... ... ... ... ... ... ...
#MN296708_YW_ND2                ... ... ... ... ... ... ... ... ... ... ... ... ... ... ... ... ... ... ... ... ... ... ... ... ... ...
#MN953621_CR_ND2                ... ... ... ... ... ... ... ... ... ... ... ... ... ... ... ... ... ... ... ... ... ... ... ... ... ...
#AJ001588.1_NZW1_ND2            ... ... ... ... ... ... ... ... ... ... ... ... ... ... ... ... ... ... ... ... ... ... ... ... ... ...

[                               111 111 111 111 111 111 111 111 111 111 111 111 111 111 122 222 222 222 222 222 222 222 222 222 222 222 ]
[                               555 666 666 666 677 777 777 778 888 888 888 999 999 999 900 000 000 001 111 111 111 222 222 222 233 333 ]
[                               789 012 345 678 901 234 567 890 123 456 789 012 345 678 901 234 567 890 123 456 789 012 345 678 901 234 ]
#Indonesian_Local_rabbit_ND2    ACA GAA GCT GCA ACC AAG TAT TTT CTA ACA CAA GCC ACA GCA TCT ATA ATC TTA ATA ATA GCT ATT ACA CTT AAT ATC
#MH98583.1_NZW2_ND2             ... ... ... ... ... ... ... ... ... ... ... ... ... ... ... ... ... ... ... ... ... ... ... ... ... ...
#MN296708_YW_ND2                ... ... ... ... ... ... ... ... ... ... ... ... ... ... ... ... ... ... ... ... ... ... ... ... ... ...
#MN953621_CR_ND2                ... ... ... ... ... ... ... ... ... ... ... ... ... ... ... ... ... ... ... ... ... G.. ... ... ... ...
#AJ001588.1_NZW1_ND2            ... ... ... ... ... ... ... ... ... ... ... ... ... ... ... ... ... ... ... ... ... ... ... ... ... ...

[                               222 222 222 222 222 222 222 222 222 222 222 222 222 222 222 222 222 222 222 222 222 223 333 333 333 333 ]
[                               333 334 444 444 444 555 555 555 566 666 666 667 777 777 777 888 888 888 899 999 999 990 000 000 000 111 ]
[                               567 890 123 456 789 012 345 678 901 234 567 890 123 456 789 012 345 678 901 234 567 890 123 456 789 012 ]
#Indonesian_Local_rabbit_ND2    CTT GAC TCC GGA CAA TGA ACC CTA ATT AAT CCA CAG AAT CAA CTT ACT CCA GTC CTA ATT ACA CTG GCA CTA ATC ATT
#MH98583.1_NZW2_ND2             ... ... ... ... ... ... ... ... ... ... ... ... ... ... ... ... ... ... ... ... ... ... ... ... ... ...
#MN296708_YW_ND2                ... ... ... ... ... ... ... ... ... ... ... ... ... ... ... ... ... ... ... ... ... ... ... ... ... ...
#MN953621_CR_ND2                ... ... ... ... ... ... ... ... ... ... ..G ... ... ... ... ... ... ... ... ... ... ... ... ... ... ...
#AJ001588.1_NZW1_ND2            ... ... ... ... ... ... ... ... ... ... ... ... ... ... ... ... ... ... ... ... ... ... ... ... ... ...
```

Genetic characteristics of complete mtDNA genome sequence of Indonesian Local rabbit (*Oryctolagus cuniculus*) (2023)
by: A. Setiaji, D. A. Lestari, N. S. Pandupuspitasari, I. Agusetyaningsih, F. A. Khan

```
[                                333 333 333 333 333 333 333 333 333 333 333 333 333 333 333 333 333 333 333 333 333 333 333 333 333 333 ]
[                                111 111 122 222 222 223 333 333 333 444 444 444 455 555 555 556 666 666 666 777 777 777 788 888 888 889 ]
[                                345 678 901 234 567 890 123 456 789 012 345 678 901 234 567 890 123 456 789 012 345 678 901 234 567 890 ]
#Indonesian_Local_rabbit_ND2     AAA CTA GGA ATA GCC CCC TTC CAC TTC TGA GTA CCA GAG GTC ACC CAA GGA GTT CCC CTA AAA TCA GGA TTA ATC CTT
#MH98583.1_NZW2_ND2              ... ... ... ... ... ... ... ... ... ... ... ... ... ... ... ... ... ... ... ... ... ... ... ... ... ...
#MN296708_YW_ND2                 ... ... ... ... ... ... ... ... ... ... ... ... ... ... ... ... ... ... ... ... ... ... ... ... ... ...
#MN953621_CR_ND2                 ... ... ... W.. ... ... ... ... ... ... ... ... ... ... ... ... ... ... ... ... ... ..R ... ... ... ...
#AJ001588.1_NZW1_ND2             ... ... ... ... ... ... ... ... ... ... ... ... ... ... ... ... ... ... ... ... ... ... ... ... ... ...

[                                333 333 333 444 444 444 444 444 444 444 444 444 444 444 444 444 444 444 444 444 444 444 444 444 444 444 ]
[                                999 999 999 000 000 000 011 111 111 112 222 222 222 333 333 333 344 444 444 445 555 555 555 666 666 666 ]
[                                123 456 789 012 345 678 901 234 567 890 123 456 789 012 345 678 901 234 567 890 123 456 789 012 345 678 ]
#Indonesian_Local_rabbit_ND2     CTC ACG TGA CAA AAA TTA GCC CCC CTA TCC ATC CTT TAC CAA ATC TCC CCA TCA ATT AAT CCA ACT ATA ATA ATA TCA
#MH98583.1_NZW2_ND2              ... ... ... ... ... ... ... ... ... ... ... ... ... ... ... ... ... ... ... ... ... ... ... ... ... ...
#MN296708_YW_ND2                 ... ... ... ... ... ... ... ... ... ... ... ... ... ... ... ... ... ... ... ... ... ... ... ... ... ...
#MN953621_CR_ND2                 ... ... ... ... ... ... ... ... ... ... ... ... ... ... ... ... ... ... ... ... ... ... ... ... ... ...
#AJ001588.1_NZW1_ND2             ... ... ... ... ... ... ... ... ... ... ... ... ... ... ... ... ... ... ... ... ... ... ... ... ... ...

[                                444 444 444 444 444 444 444 444 444 444 455 555 555 555 555 555 555 555 555 555 555 555 555 555 555 555 ]
[                                677 777 777 778 888 888 888 999 999 999 900 000 000 001 111 111 111 222 222 222 233 333 333 334 444 444 ]
[                                901 234 567 890 123 456 789 012 345 678 901 234 567 890 123 456 789 012 345 678 901 234 567 890 123 456 ]
#Indonesian_Local_rabbit_ND2     GTA GCC ATT CTA TCA ATT ATA GTT GGC GGT TGA GGT GGA CTA AAC CAA ACC CAA CTA CGA AAA ATC CTA GCA TAC TCT
#MH98583.1_NZW2_ND2              ... ... ... ... ... ... ... ... ... ... ... ... ... ... ... ... ... ... ... ... ... ... ... ... ... ...
#MN296708_YW_ND2                 ... ... ... ... ... ... ... ... ... ... ... ... ... ... ... ... ... ... ... ... ... ... ... ... ... ...
#MN953621_CR_ND2                 ... ... ... ... ... ... ... ... ... ... ... ... ... ... ... ... ... ... ... ... ... ... ... ... ... ...
#AJ001588.1_NZW1_ND2             ... ... ... ... ... ... ... ... ... ... ... ... ... ... ... ... ... ... ... ... ... ... ... ... ... ...

[                                555 555 555 555 555 555 555 555 555 555 555 555 555 555 555 555 555 556 666 666 666 666 666 666 666 666 ]
[                                444 555 555 555 566 666 666 667 777 777 777 888 888 888 899 999 999 990 000 000 000 111 111 111 122 222 ]
[                                789 012 345 678 901 234 567 890 123 456 789 012 345 678 901 234 567 890 123 456 789 012 345 678 901 234 ]
#Indonesian_Local_rabbit_ND2     TCA ATC GCC CAC ATA GGA TGA ATA GCA GCT ATC ATC ACA TTT AAC CCA AAT ACT ATA GTC CTA AAC CTA ATT ATC TAT
#MH98583.1_NZW2_ND2              ... ... ... ... ... ... ... ... ... ... ... ... ... ... ... ... ... ... ... ... ... ... ... ... ... ...
#MN296708_YW_ND2                 ... ... ... ... ... ... ... ... ... ... ... ... ... ... ... ... ... ... ... ... ... ... ... ... ... ...
#MN953621_CR_ND2                 ... ... ... ... ... ... ... ... ... ... ... ... ... ... ... ... ... ... ... ... ... ... ... ... ... ...
#AJ001588.1_NZW1_ND2             ... ... ... ... ... ... ... ... ... ... ... ... ... ... ... ... ... ... ... ... ... ... ... ... ... ...
```

Genetic characteristics of complete mtDNA genome sequence of Indonesian Local rabbit (*Oryctolagus cuniculus*) (2023)
by: A. Setiaji, D. A. Lestari, N. S. Pandupuspitasari, I. Agusetyaningsih, F. A. Khan

```
[                                 666 666 666 666 666 666 666 666 666 666 666 666 666 666 666 666 666 666 666 666 666 666 666 666 666 777 ]
[                                 222 223 333 333 333 444 444 444 455 555 555 556 666 666 666 777 777 777 788 888 888 889 999 999 999 000 ]
[                                 567 890 123 456 789 012 345 678 901 234 567 890 123 456 789 012 345 678 901 234 567 890 123 456 789 012 ]
#Indonesian_Local_rabbit_ND2      ATT CTC ATG ACA ATC CCT ATA TTC ATA ATA TTC ATA CAG CAC TCA AGT ACG ACC ACT CTA TCA CTA TCA CAG ATG TGA
#MH98583.1_NZW2_ND2               ... ... ... ... ... ... ... ... ... ... ... ... ... ... ... ... ... ... ... ... ... ... ... ... ... ...
#MN296708_YW_ND2                  ... ... ... ... ... ... ... ... ... ... ... ... ... ... ... ... ... ... ... ... ... ... ... ... ... ...
#MN953621_CR_ND2                  ... ... ... ... ... ... ... ... ... ... ... ... ... ... ... ... ... ... ... ... ... ... ... ... ... ...
#AJ001588.1_NZW1_ND2              ... ... ... ... ... ... ... ... ... ... ... ... ... ... ... ... ... ... ... ... ... ... ... ... ... ...

[                                 777 777 777 777 777 777 777 777 777 777 777 777 777 777 777 777 777 777 777 777 777 777 777 777 777 777 ]
[                                 000 000 011 111 111 112 222 222 222 333 333 333 344 444 444 445 555 555 555 666 666 666 677 777 777 778 ]
[                                 345 678 901 234 567 890 123 456 789 012 345 678 901 234 567 890 123 456 789 012 345 678 901 234 567 890 ]
#Indonesian_Local_rabbit_ND2      AAC AAA AAT CCA CTA ATA GTA TCT ACT ATC CTA ATC ACC CTA ATA TCC CTA GGA GGC CTG CCC CCA CTC ACA GGT TTT
#MH98583.1_NZW2_ND2               ... ... ... ... ... ... ... ... ... ... ... ... ... ... ... ... ... ... ... ... ... ... ... ... ... ...
#MN296708_YW_ND2                  ... ... ... ... ... ... ... ... ... ... ... ... ... ... ... ... ... ... ... ... ... ... ... ... ... ...
#MN953621_CR_ND2                  ... ... ... ... ... ... ... ... ... ... ... ... ... ... ... ... ... ... ... ... ... ... ... ... ... ...
#AJ001588.1_NZW1_ND2              ... ... ... ... ... ... ... ... ... ... ... ... ... ... ... ... ... ... ... ... ... ... ... ... ... ...

[                                 777 777 777 777 777 777 788 888 888 888 888 888 888 888 888 888 888 888 888 888 888 888 888 888 888 888 ]
[                                 888 888 888 999 999 999 900 000 000 001 111 111 111 222 222 222 233 333 333 334 444 444 444 555 555 555 ]
[                                 123 456 789 012 345 678 901 234 567 890 123 456 789 012 345 678 901 234 567 890 123 456 789 012 345 678 ]
#Indonesian_Local_rabbit_ND2      ATC CCT AAA TGA ATT ATC ATC CAG GAA TTA ACA AAA AAT GGC AAT ATC ATT TTA CCC ACC GCT ATA GCC ATG CTT GCC
#MH98583.1_NZW2_ND2               ... ... ... ... ... ... ... ... ... ... ... ... ... ... ... ... ... ... ... ... ... ... ... ... ... ...
#MN296708_YW_ND2                  ... ... ... ... ... ... ... ... ... ... ... ... ... ... ... ... ... ... ... ... ... ... ... ... ... ...
#MN953621_CR_ND2                  ... ... ... ... ... ... ... ... ... ... ... ... ... ... ... ... ... ... ... ... ... ... ... ... ... ...
#AJ001588.1_NZW1_ND2              ... ... ... ... ... ... ... ... ... ... ... ... ... ... ... ... ... ... ... ... ... ... ... ... ... ...

[                                 888 888 888 888 888 888 888 888 888 888 888 888 888 889 999 999 999 999 999 999 999 999 999 999 999 999 ]
[                                 566 666 666 667 777 777 777 888 888 888 899 999 999 990 000 000 000 111 111 111 122 222 222 223 333 333 ]
[                                 901 234 567 890 123 456 789 012 345 678 901 234 567 890 123 456 789 012 345 678 901 234 567 890 123 456 ]
#Indonesian_Local_rabbit_ND2      CTC CTA AAT CTA TAT TTT TAT ATA CGC CTT ATC TAT TCT TCC TCA CTA ACT ATA TTC CCT ACC ACC AAC AAT CTA AAA
#MH98583.1_NZW2_ND2               ... ... ... ... ... ... ... ... ... ... ... ... ... ... ... ... ... ... ... ... ... ... ... ... ... ...
#MN296708_YW_ND2                  ... ... ... ... ... ... ... ... ... ... ... ... ... ... ... ... ... ... ... ... ... ... ... ... ... ...
#MN953621_CR_ND2                  ... ... ... ... ... ... ... ... ... ... ... ... ... ... ... ... ... ... ... ... ... ... ... ... ... ...
#AJ001588.1_NZW1_ND2              ... ... ... ... ... ... ... ... ... ... ... ... ... ... ... ... ... ... ... ... ... ... ... ... ... ...
```

Genetic characteristics of complete mtDNA genome sequence of Indonesian Local rabbit (*Oryctolagus cuniculus*) (2023)
by: A. Setiaji, D. A. Lestari, N. S. Pandupuspitasari, I. Agusetyaningsih, F. A. Khan

```
[                                                                                                                111 111 111 111 111 ]
[                                 999 999 999 999 999 999 999 999 999 999 999 999 999 999 999 999 999 999 999 999 999 000 000 000 000 000 ]
[                                 333 444 444 444 455 555 555 556 666 666 666 777 777 777 788 888 888 889 999 999 999 000 000 000 011 111 ]
[                                 789 012 345 678 901 234 567 890 123 456 789 012 345 678 901 234 567 890 123 456 789 012 345 678 901 234 ]
#Indonesian_Local_rabbit_ND2     ATA AAA TGA CAA TTT GAA TCA ACA AAA CGC ATA CCC CTC ATC ACT CCA CTA ATT ATT TTA TCA ACT ATA CTC CTC CCC
#MH98583.1_NZW2_ND2              ... ... ... ... ... ... ... ... ... ... ... ... ... ... ... ... ... ... ... ... ... ... ... ... ... ...
#MN296708_YW_ND2                 ... ... ... ... ... ... ... ... ... ... ... ... ... ... ... ... ... ... ... ... ... ... ... ... ... ...
#MN953621_CR_ND2                 ... ... ... ... ... ... ... ... ... ... ... ... ... ... ... ... ... ... ... ... ... ... ... ... ... ...
#AJ001588.1_NZW1_ND2             ... ... ... ... ... ... ... ... ... ... ... ... ... ... ... ... ... ... ... ... ... ... ... ... ... ...

[                                 111 111 111 111 111 111 111 111 111 111 ]
[                                 000 000 000 000 000 000 000 000 000 000 ]
[                                 111 112 222 222 222 333 333 333 344 444 ]
[                                 567 890 123 456 789 012 345 678 901 234 ]
#Indonesian_Local_rabbit_ND2     CTC ACA CCA GCA CTC TCA GTA TTA AAT TAA
#MH98583.1_NZW2_ND2              ... ... ... ... ... ... ... ... ... ...
#MN296708_YW_ND2                 ... ... ... ... ... ... ... ... ... ...
#MN953621_CR_ND2                 ... ... ... ... ... ... ... ... ... ...
#AJ001588.1_NZW1_ND2             ... ... ... ... ... ... ... ... ... ...
```

Genetic characteristics of complete mtDNA genome sequence of Indonesian Local rabbit (*Oryctolagus cuniculus*) (2023)
by: A. Setiaji, D. A. Lestari, N. S. Pandupuspitasari, I. Agusetyaningsih, F. A. Khan

```
#MEGA
ND3 rabbit alignment

[                                           111 111 111 122 222 222 223 333 333 333 444 444 444 455 555 555 556 666 666 666 777 777 777 ]
[                               123 456 789 012 345 678 901 234 567 890 123 456 789 012 345 678 901 234 567 890 123 456 789 012 345 678 ]
#Indonesian_Local_rabbit_ND3    ATA AAC CTA ATA CTA GTT CTA TTA ATT AAC ACC ACC ATT TCC TTA GTC CTA GTT ACA ATT GCA TTC TGA CTC CCT CAA
#MH985853.1_NZW2_ND3            ... ... ..G ... ... ... ... ... ... ... ... ... ... ... ... ... ... ... ... ... ... ... ... ... ... ...
#MN296708_YW_ND3                ... ... ..G ... ... ... ... ... ... ... ... ... ... ... ... ... ... ... ... ... ... ... ... ... ... ...
#AJ001588.1_NWZ1_ND3            ... ... ..G ... ... ... ... ... ... ... ... ... ... ... ... ... ... ... ... ... ... ... ... ... ... ...
#MN953621.1_CR_ND3              ... ... ..G ... ... ... ... ... ... ... ... ... ... ... ... ... ... ... ... ... ... ... ... ... ... ...

[                                                           111 111 111 111 111 111 111 111 111 111 111 111 111 111 111 111 111 111 111 ]
[                               788 888 888 889 999 999 999 000 000 000 011 111 111 112 222 222 222 333 333 333 344 444 444 445 555 555 ]
[                               901 234 567 890 123 456 789 012 345 678 901 234 567 890 123 456 789 012 345 678 901 234 567 890 123 456 ]
#Indonesian_Local_rabbit_ND3    TTA AAT ATC TAT TCA GAA AAA TCA AGC CCT TAT GAA TGC GGA TTC GAT CCC ATA GGA TCA GCA CGA CTC CCT TTC TCT
#MH985853.1_NZW2_ND3            ... ... ... ... ... ... ... ... ... ... ... ... ... ... ... ... ... ... ... ... ... ... ... ... ... ...
#MN296708_YW_ND3                ... ... ... ... ... ... ... ... ... ... ... ... ... ... ... ... ... ... ... ... ... ... ... ... ... ...
#AJ001588.1_NWZ1_ND3            ... ... ... ... ... ... ... ... ... ... ... ... ... ... ... ... ... ... ... ... ... ... ... ... ... ...
#MN953621.1_CR_ND3              ... ... ... ... ... ... ... ... ... ... ... ... ... ... ... ... ... ... ... ... ... ... ... ... ... ...

[                               111 111 111 111 111 111 111 111 111 111 111 111 111 111 122 222 222 222 222 222 222 222 222 222 222 222 ]
[                               555 666 666 666 677 777 777 778 888 888 888 999 999 999 900 000 000 001 111 111 111 222 222 222 233 333 ]
[                               789 012 345 678 901 234 567 890 123 456 789 012 345 678 901 234 567 890 123 456 789 012 345 678 901 234 ]
#Indonesian_Local_rabbit_ND3    ATA AAA TTT TTT CTA GTA GCC ATT ACA TTT TTA CTT TTT GAC CTA GAA ATT GCT CTC CTC CTC CCA CTC CCA TGA GCC
#MH985853.1_NZW2_ND3            ... ... ... ... ... ... ... ... ... ... ... ... ... ... ... ... ... ... ... ... ... ... ... ... ... ...
#MN296708_YW_ND3                ... ... ... ... ... ... ... ... ... ... ... ... ... ... ... ... ... ... ... ... ... ... ... ... ... ...
#AJ001588.1_NWZ1_ND3            ... ... ... ... ... ... ... ... ... ... ... ... ... ... ... ... ... ... ... ... ... ... ... ... ... ...
#MN953621.1_CR_ND3              ... ... ... ... ... ... ... ... ... ... ... ... ... ... ... ... ... ... ... ... ... ... ... ... ... ...

[                               222 222 222 222 222 222 222 222 222 222 222 222 222 222 222 222 222 222 222 222 222 223 333 333 333 333 ]
[                               333 334 444 444 444 555 555 555 566 666 666 667 777 777 777 888 888 888 899 999 999 990 000 000 000 111 ]
[                               567 890 123 456 789 012 345 678 901 234 567 890 123 456 789 012 345 678 901 234 567 890 123 456 789 012 ]
#Indonesian_Local_rabbit_ND3    GCA CAA TTT AAT AAT TTA AAC CTA GTC CTC ATC ATA GCA CTT ATG TTA ATC TCA ATC TTA GCC CTT GGA TTA GCC TAC
#MH985853.1_NZW2_ND3            ... ... ... ... ... ... ... ... ... ... ... ... ... ... ... ... ... ... ... ... ... ... ... ... ... ...
#MN296708_YW_ND3                ... ... ... ... ... ... ... ... ... ... ... ... ... ... ... ... ... ... ... ... ... ... ... ... ... ...
#AJ001588.1_NWZ1_ND3            ... ... ... ... ... ... ... ... ... ... ... ... ... ... ... ... ... ... ... ... ... ... ... ... ... ...
#MN953621.1_CR_ND3              ... ... ... ... ... ... ... ... ... ... ... ... ... ... ... ... ... ... ... ... ... ... ... ... ... ...
```

Genetic characteristics of complete mtDNA genome sequence of Indonesian Local rabbit (*Oryctolagus cuniculus*) (2023)
by: A. Setiaji, D. A. Lestari, N. S. Pandupuspitasari, I. Agusetyaningsih, F. A. Khan

```
[                                333 333 333 333 333 333 333 333 333 333 333 333 333 333 333 ]
[                                111 111 122 222 222 223 333 333 333 444 444 444 455 555 555 ]
[                                345 678 901 234 567 890 123 456 789 012 345 678 901 234 567 ]
#Indonesian_Local_rabbit_ND3     GAA TGA ATC CAA AAA GGA CTA GAA TGA GTT GAA TAT GAT AAT TAG
#MH985853.1_NZW2_ND3             ... ... ... ... ... ... ... ... ... ... ... ... ... ... ...
#MN296708_YW_ND3                 ... ... ... ... ... ... ... ... ... ... ... ..- --- --- ---
#AJ001588.1_NWZ1_ND3             ... ... ... ... ... ... ... ... ... ... ... ..- --- --- ---
#MN953621.1_CR_ND3               ... ... ... ... ... ... ... ... ... ... ... ..- --- --- ---
```

```
#MEGA
ND4 rabbit alignment

[                                               111 111 111 122 222 222 223 333 333 333 444 444 444 455 555 555 556 666 666 666 777 777 777 ]
[                                   123 456 789 012 345 678 901 234 567 890 123 456 789 012 345 678 901 234 567 890 123 456 789 012 345 678 ]
#Indonesian_Local_rabbit_ND4        ATG CTA AAA ACG ATT ATC CCC ACA ATC ATA CTT ATT CCA ACC GTA TGG TGA TCA AAA CCC CAT ATA ATT TGA ATT AAC
#MH985853.1_NZW2_ND4                ... ... ... ... ... ... ... ... ... ... ... ... ... ... ... ... ... ... ... ... ... ... ... ... ... ...
#MN296708_YW_ND4                    ... ... ... ... ... ... ... ... ... ... ... ... ... ... ... ... ... ... ... ... ... ... ... ... ... ...
#AJ001588.1_NZW1_ND4                ... ... ... ... ... ... ... ... ... ... ... ... ... ... ... ... ... ... ... ... ... ... ... ... ... ...
#MN953621.1_CR_ND4                  ... ... ... ... ... ... ... ... ... ... ... ... ... ... ... ... ... ... ... ... ... ... ... ... ... ...

[                                                               111 111 111 111 111 111 111 111 111 111 111 111 111 111 111 111 111 111 111 ]
[                                   788 888 888 889 999 999 999 000 000 000 011 111 111 112 222 222 222 333 333 333 344 444 444 445 555 555 ]
[                                   901 234 567 890 123 456 789 012 345 678 901 234 567 890 123 456 789 012 345 678 901 234 567 890 123 456 ]
#Indonesian_Local_rabbit_ND4        GCA ACA GTT TAC AGC CTA TTA ATT AGC CTA ACT ACC CTC TCT CTA CTT AAT CAG CCA AGT GAC ACC AAC CTA AAC TTC
#MH985853.1_NZW2_ND4                ... ... ... ... ... ... ... ... ... ... ... ... ... ... ... ... ... ... ... ... ... ... ... ... ... ...
#MN296708_YW_ND4                    ... ... ... ... ... ... ... ... ... ... ... ... ... ... ... ... ... ... ... ... ... ... ... ... ... ...
#AJ001588.1_NZW1_ND4                ... ... ... ... ... ... ... ... ... ... ... ... ... ... ... ... ... ... ... ... ... ... ... ... ... ...
#MN953621.1_CR_ND4                  ... ... ... ... ... ... ... ... ... ... ... ... ... ... ... ... ... ... ... ... ... ... ... ... ... ...

[                                   111 111 111 111 111 111 111 111 111 111 111 111 111 111 122 222 222 222 222 222 222 222 222 222 222 222 ]
[                                   555 666 666 666 677 777 777 778 888 888 888 999 999 999 900 000 000 001 111 111 111 222 222 222 233 333 ]
[                                   789 012 345 678 901 234 567 890 123 456 789 012 345 678 901 234 567 890 123 456 789 012 345 678 901 234 ]
#Indonesian_Local_rabbit_ND4        TCA ACC ACA TTT TTT TCA GAT GCC TTA TCC ACT CCT CTC CTG ATG TTA ACG ACC TGA CTT CTT CCA CTC ATA ATC TTA
#MH985853.1_NZW2_ND4                ... ... ... ... ... ... ... ... ... ... ... ... ... ... ... ... ... ... ... ... ... ... ... ... ... ...
#MN296708_YW_ND4                    ... ... ... ... ... ... ... ... ... ... ... ... ... ... ... ... ... ... ... ... ... ... ... ... ... ...
#AJ001588.1_NZW1_ND4                ... ... ... ... ... ... ... ... ... ... ... ... ... ... ... ... ... ... ... ... ... ... ... ... ... ...
#MN953621.1_CR_ND4                  ... ... ... ... ... ... ... ... ... ... ... ... ... ... ... ... ... ... ... ... ... ... ... ... ... ...

[                                   222 222 222 222 222 222 222 222 222 222 222 222 222 222 222 222 222 222 222 222 222 223 333 333 333 333 ]
[                                   333 334 444 444 444 555 555 555 566 666 666 667 777 777 777 888 888 888 899 999 999 990 000 000 000 111 ]
[                                   567 890 123 456 789 012 345 678 901 234 567 890 123 456 789 012 345 678 901 234 567 890 123 456 789 012 ]
#Indonesian_Local_rabbit_ND4        GCA AGC CAA CAT CAC TTA GAC AAA GAA ACC TTA ACC CGT AAA AAA ATT TAT ATT TCT CTT TTA ATT TCC TTA CAA GTA
#MH985853.1_NZW2_ND4                ... ... ... ... ... ... ... ... ... ... ... ... ... ... ... ... ... ... ... ... ... ... ... ... ... ...
#MN296708_YW_ND4                    ... ... ... ... ... ... ... ... ... ... ... ... ... ... ... ... ... ... ... ... ... ... ... ... ... ...
#AJ001588.1_NZW1_ND4                ... ... ... ... ... ... ... ... ... ... ... ... ... ... ... ... ... ... ... ... ... ... ... ... ... ...
#MN953621.1_CR_ND4                  ... ... ... ... ... ... ... ... ... ... ... ... ... ... ... ... ... ... ... ... ... ... ... ... ... ...
```

Genetic characteristics of complete mtDNA genome sequence of Indonesian Local rabbit (*Oryctolagus cuniculus*) (2023)
by: A. Setiaji, D. A. Lestari, N. S. Pandupuspitasari, I. Agusetyaningsih, F. A. Khan

```
[                               333 333 333 333 333 333 333 333 333 333 333 333 333 333 333 333 333 333 333 333 333 333 333 333 333 333 ]
[                               111 111 122 222 222 223 333 333 333 444 444 444 455 555 555 556 666 666 666 777 777 777 788 888 888 889 ]
[                               345 678 901 234 567 890 123 456 789 012 345 678 901 234 567 890 123 456 789 012 345 678 901 234 567 890 ]
#Indonesian_Local_rabbit_ND4    TTC CTA GTA ATA ACA TTT TCA GCC ACA GAA TTT ATC CTG TTT TAT ATT CTA TTT GAA GCC ACA CTG ATT CCC ACT CTT
#MH985853.1_NZW2_ND4            ... ... ... ... ... ... ... ... ... ... ... ... ... ... ... ... ... ... ... ... ... ... ... ... ... ...
#MN296708_YW_ND4                ... ... ... ... ... ... ... ... ... ... ... ... ... ... ... ... ... ... ... ... ... ... ... ... ... ...
#AJ001588.1_NZW1_ND4            ... ... ... ... ... ... ... ... ... ... ... ... ... ... ... ... ... ... ... ... ... ... ... ... ... ...
#MN953621.1_CR_ND4              ... ... ... ... ... ... ... ... ... ... ... ... ... ... ... ... ... ... ... ... ... ... ... ... ... ...

[                               333 333 333 444 444 444 444 444 444 444 444 444 444 444 444 444 444 444 444 444 444 444 444 444 444 444 ]
[                               999 999 999 000 000 000 011 111 111 112 222 222 222 333 333 333 344 444 444 445 555 555 555 666 666 666 ]
[                               123 456 789 012 345 678 901 234 567 890 123 456 789 012 345 678 901 234 567 890 123 456 789 012 345 678 ]
#Indonesian_Local_rabbit_ND4    ATC ATT ATT ACA CGA TGA GGT AAT CAA ACA GAG CGA CTC AAC GCA GGA ACC TAC TTC CTA TTT TAT ACC CTC ATA GGA
#MH985853.1_NZW2_ND4            ... ... ... ... ... ... ... ... ... ... ... ... ... ... ... ... ... ... ... ... ... ... ... ... ... ...
#MN296708_YW_ND4                ... ... ... ... ... ... ... ... ... ... ... ... ... ... ... ... ... ... ... ... ... ... ... ... ... ...
#AJ001588.1_NZW1_ND4            ... ... ... ... ... ... ... ... ... ... ... ... ... ... ... ... ... ... ... ... ... ... ... ... ... ...
#MN953621.1_CR_ND4              ... ... ... ... ... ... ... ... ... ... ... ... ... ... ... ... ... ... ... ... ... ... ... ... ... ...

[                               444 444 444 444 444 444 444 444 444 444 455 555 555 555 555 555 555 555 555 555 555 555 555 555 555 555 ]
[                               677 777 777 778 888 888 888 999 999 999 900 000 000 001 111 111 111 222 222 222 233 333 333 334 444 444 ]
[                               901 234 567 890 123 456 789 012 345 678 901 234 567 890 123 456 789 012 345 678 901 234 567 890 123 456 ]
#Indonesian_Local_rabbit_ND4    TCC CTC CCC CTT CTA GTG GCT TTA ATT TAT TTA CAG AAC TCT ATA GGA TCT CTA AAC TTC CTC CTA CTT CAA CTT ATA
#MH985853.1_NZW2_ND4            ... ... ... ... ... ... ... ... ... ... ... ... ... ... ... ... ... ... ... ... ... ... ... ... ... ...
#MN296708_YW_ND4                ... ... ... ... ... ... ... ... ... ... ... ... ... ... ... ... ... ... ... ... ... ... ... ... ... ...
#AJ001588.1_NZW1_ND4            ... ... ... ... ... ... ... ... ... ... ... ... ... ... ... ... ... ... ... ... ... ... ... ... ... ...
#MN953621.1_CR_ND4              ... ... ... ... ... ... ... ... ... ... ... ... ... ... ... ... ... ... ... ... ... ... ... ... ... ...

[                               555 555 555 555 555 555 555 555 555 555 555 555 555 555 555 555 555 556 666 666 666 666 666 666 666 666 ]
[                               444 555 555 555 566 666 666 667 777 777 777 888 888 888 899 999 999 990 000 000 000 111 111 111 122 222 ]
[                               789 012 345 678 901 234 567 890 123 456 789 012 345 678 901 234 567 890 123 456 789 012 345 678 901 234 ]
#Indonesian_Local_rabbit_ND4    AAT AAA TCC ATT ACA ACA TCG TGA TCA AAC TCA CTG ATA TGA CTA GCA TGC ATA ATA GCA TTT TTA GTC AAA ATA CCC
#MH985853.1_NZW2_ND4            ... ... ... ... ... ... ... ... ... ... ... ... ... ... ... ... ... ... ... ... ... ... ... ... ... ...
#MN296708_YW_ND4                ... ... ... ... ... ... ... ... ... ... ... ... ... ... ... ... ... ... ... ... ... ... ... ... ... ...
#AJ001588.1_NZW1_ND4            ... ... ... ... ... ... ... ... ... ... ... ... ... ... ... ... ... ... ... ... ... ... ... ... ... ...
#MN953621.1_CR_ND4              ... ... ... ... ... ... ... ... ... ... ... ... ... ... ... ... ... ... ... ... ... ... ... ... ... ...
```

Genetic characteristics of complete mtDNA genome sequence of Indonesian Local rabbit (*Oryctolagus cuniculus*) (2023)
by: A. Setiaji, D. A. Lestari, N. S. Pandupuspitasari, I. Agusetyaningsih, F. A. Khan

```
[                                666 666 666 666 666 666 666 666 666 666 666 666 666 666 666 666 666 666 666 666 666 666 666 666 666 777 ]
[                                222 223 333 333 333 444 444 444 455 555 555 556 666 666 666 777 777 777 788 888 888 889 999 999 999 000 ]
[                                567 890 123 456 789 012 345 678 901 234 567 890 123 456 789 012 345 678 901 234 567 890 123 456 789 012 ]
#Indonesian_Local_rabbit_ND4     TTA TAT GGG CTT CAC CTG TGA TTA CCA AAA GCT CAC GTT GAA GCC CCC ATC GCT GGC TCA ATA GTC CTG GCA GCT ATT
#MH985853.1_NZW2_ND4             ... ... ... ... ... ... ... ... ... ... ... ... ... ... ... ... ... ... ... ... ... ... ... ... ... ...
#MN296708_YW_ND4                 ... ... ... ... ... ... ... ... ... ... ... ... ... ... ... ... ... ... ... ... ... ... ... ... ... ...
#AJ001588.1_NZW1_ND4             ... ... ... ... ... ... ... ... ... ... ... ... ... ... ... ... ... ... ... ... ... ... ... ... ... ...
#MN953621.1_CR_ND4               ... ... ... ... ... ... ... ... ... ... ... ... ... ... ... ... ... ... ... ... ... ... ... ... ... ...

[                                777 777 777 777 777 777 777 777 777 777 777 777 777 777 777 777 777 777 777 777 777 777 777 777 777 777 ]
[                                000 000 011 111 111 112 222 222 222 333 333 333 344 444 444 445 555 555 555 666 666 666 677 777 777 778 ]
[                                345 678 901 234 567 890 123 456 789 012 345 678 901 234 567 890 123 456 789 012 345 678 901 234 567 890 ]
#Indonesian_Local_rabbit_ND4     CTA CTT AAA CTA GGC GGC TAC GGA ATA ATA CGC ATT ACC ATC CTA CTC AGC CCT ATT ACA GAC TAT ATA GCC TAC CCA
#MH985853.1_NZW2_ND4             ... ... ... ... ... ... ... ... ... ... ... ... ... ... ... ... ... ... ... ... ... ... ... ... ... ...
#MN296708_YW_ND4                 ... ... ... ... ... ... ... ... ... ... ... ... ... ... ... ... ... ... ... ... ... ... ... ... ... ...
#AJ001588.1_NZW1_ND4             ... ... ... ... ... ... ... ... ... ... ... ... ... ... ... ... ... ... ... ... ... ... ... ... ... ...
#MN953621.1_CR_ND4               ... ... ... ... ... ... ... ... ... ... ... ... ... ... ... ... ... ... ... ... ... ... ... ... ... ...

[                                777 777 777 777 777 777 788 888 888 888 888 888 888 888 888 888 888 888 888 888 888 888 888 888 888 888 ]
[                                888 888 888 999 999 999 900 000 000 001 111 111 111 222 222 222 233 333 333 334 444 444 444 555 555 555 ]
[                                123 456 789 012 345 678 901 234 567 890 123 456 789 012 345 678 901 234 567 890 123 456 789 012 345 678 ]
#Indonesian_Local_rabbit_ND4     TTC CTC ATA CTC TCC CTC TGA GGC ATA ATT ATA ACC AGC TCT ATC TGT CTC CGG CAA ACA GAC CTC AAA TCA CTC ATT
#MH985853.1_NZW2_ND4             ... ... ... ... ... ... ... ... ... ... ... ... ... ... ... ... ... ... ... ... ... ... ... ... ... ...
#MN296708_YW_ND4                 ... ... ... ... ... ... ... ... ... ... ... ... ... ... ... ... ... ... ... ... ... ... ... ... ... ...
#AJ001588.1_NZW1_ND4             ... ... ... ... ... ... ... ... ... ... ... ... ... ... ... ... ... ... ... ... ... ... ... ... ... ...
#MN953621.1_CR_ND4               ... ... ... ... ... ... ... ... ... ... ... ... ... ... ... ... ... ... ... ... ... ... ... ... ... ...

[                                888 888 888 888 888 888 888 888 888 888 888 888 888 889 999 999 999 999 999 999 999 999 999 999 999 999 ]
[                                566 666 666 667 777 777 777 888 888 888 899 999 999 990 000 000 000 111 111 111 122 222 222 223 333 333 ]
[                                901 234 567 890 123 456 789 012 345 678 901 234 567 890 123 456 789 012 345 678 901 234 567 890 123 456 ]
#Indonesian_Local_rabbit_ND4     GCT TAT TCA TCA GTG AGC CAC ATA GCC CTA GTA ATC GTA GCC ATC CTT ATT CAA ACA CCA TGA AGC TTT ATA GGC GCC
#MH985853.1_NZW2_ND4             ... ... ... ... ... ... ... ... ... ... ... ... ... ... ... ... ... ... ... ... ... ... ... ... ... ...
#MN296708_YW_ND4                 ... ... ... ... ... ... ... ... ... ... ... ... ... ... ... ... ... ... ... ... ... ... ... ... ... ...
#AJ001588.1_NZW1_ND4             ... ... ... ... ... ... ... ... ... ... ... ... ... ... ... ... ... ... ... ... ... ... ... ... ... ...
#MN953621.1_CR_ND4               ... ... ... ... ... ... ... ... ... ... ... ... ... ... ... ... ... ... ... ... ... ... ... ... ... ...
```

Genetic characteristics of complete mtDNA genome sequence of Indonesian Local rabbit (*Oryctolagus cuniculus*) (2023)
by: A. Setiaji, D. A. Lestari, N. S. Pandupuspitasari, I. Agusetyaningsih, F. A. Khan

```
[                                                                                                                   111 111 111 111 111 ]
[                               999 999 999 999 999 999 999 999 999 999 999 999 999 999 999 999 999 999 999 999 999 000 000 000 000 000 ]
[                               333 444 444 444 455 555 555 556 666 666 666 777 777 777 788 888 888 889 999 999 999 000 000 000 011 111 ]
[                               789 012 345 678 901 234 567 890 123 456 789 012 345 678 901 234 567 890 123 456 789 012 345 678 901 234 ]
#Indonesian_Local_rabbit_ND4    ACC GCT CTA ATA ATC GCA CAT GGC CTT ACT TCC TCA TTA TTA TTC TGC CTA GCC AAT TCC AAC TAC GAG CGC ATT CAC
#MH985853.1_NZW2_ND4            ... ... ... ... ... ... ... ... ... ... ... ... ... ... ... ... ... ... ... ... ... ... ... ... ... ...
#MN296708_YW_ND4                ... ... ... ... ... ... ... ... ... ... ... ... ... ... ... ... ... ... ... ... ... ... ... ... ... ...
#AJ001588.1_NZW1_ND4            ... ... ... ... ... ... ... ... ... ... ... ... ... ... ... ... ... ... ... ... ... ... ... ... ... ...
#MN953621.1_CR_ND4              ... ... ... ... ... ... ... ... ... ... ... ... ... ... ... ... ... ... ... ... ... ... ... ... ... ...

[                               111 111 111 111 111 111 111 111 111 111 111 111 111 111 111 111 111 111 111 111 111 111 111 111 111 111 ]
[                               000 000 000 000 000 000 000 000 000 000 000 000 000 000 000 000 000 000 000 000 000 000 000 000 000 000 ]
[                               111 112 222 222 222 333 333 333 344 444 444 445 555 555 555 666 666 666 677 777 777 778 888 888 888 999 ]
[                               567 890 123 456 789 012 345 678 901 234 567 890 123 456 789 012 345 678 901 234 567 890 123 456 789 012 ]
#Indonesian_Local_rabbit_ND4    AGC CGA ACC ATA CTA CTA GCC CGA GGC CTA CAG ACT ATT CTT CCA CTA ATA GCA GCA TGA TGA CTA GTT GCT AGC CTT
#MH985853.1_NZW2_ND4            ... ... ... ... ... ... ... ... ... ... ... ... ... ... ... ... ... ... ... ... ... ... ... ... ... ...
#MN296708_YW_ND4                ... ... ... ... ... ... ... ... ... ... ... ... ... ... ... ... ... ... ... ... ... ... ... ... ... ...
#AJ001588.1_NZW1_ND4            ... ... ... ... ... ... ... ... ... ... ... ... ... ... ... ... ... ... ... ... ... ... ... ... ... ...
#MN953621.1_CR_ND4              ... ... ... ... ... ... ... ... ... ... ... ... ... ... ... ... ... ... ... ... ... ... ... ... ... ...

[                               111 111 111 111 111 111 111 111 111 111 111 111 111 111 111 111 111 111 111 111 111 111 111 111 111 111 ]
[                               000 000 011 111 111 111 111 111 111 111 111 111 111 111 111 111 111 111 111 111 111 111 111 111 111 111 ]
[                               999 999 900 000 000 001 111 111 111 222 222 222 233 333 333 334 444 444 444 555 555 555 566 666 666 667 ]
[                               345 678 901 234 567 890 123 456 789 012 345 678 901 234 567 890 123 456 789 012 345 678 901 234 567 890 ]
#Indonesian_Local_rabbit_ND4    ACC AAC CTG GCC CTA CCT CCA ACT ATT AAT CTG CTA GGG GAA CTT CTT ATC ATT ATG GCC TCC TTC TCA TGA TCT AAC
#MH985853.1_NZW2_ND4            ... ... ... ... ... ... ... ... ... ... ... ... ... ... ... ... ... ... ... ... ... ... ... ... ... ...
#MN296708_YW_ND4                ... ... ... ... ... ... ... ... ... ... ... ... ... ... ... ... ... ... ... ... ... ... ... ... ... ...
#AJ001588.1_NZW1_ND4            ... ... ... ... ... ... ... ... ... ... ... ... ... ... ... ... ... ... ... ... ... ... ... ... ... ...
#MN953621.1_CR_ND4              ... ... ... ... ... ... ... ... ... ... ... ... ... ... ... ... ... ... ... ... ... ... ... ... ... ...

[                               111 111 111 111 111 111 111 111 111 111 111 111 111 111 111 111 111 111 111 111 111 111 111 111 111 111 ]
[                               111 111 111 111 111 111 111 111 111 112 222 222 222 222 222 222 222 222 222 222 222 222 222 222 222 222 ]
[                               777 777 777 888 888 888 899 999 999 990 000 000 000 111 111 111 122 222 222 223 333 333 333 444 444 444 ]
[                               123 456 789 012 345 678 901 234 567 890 123 456 789 012 345 678 901 234 567 890 123 456 789 012 345 678 ]
#Indonesian_Local_rabbit_ND4    CTC ACA ATC ATC CTG ATA GGG ACT AAC GTC CTA ATC ACG GCC CTC TAC TCC CTC TAT ATA CTA TCA ACC ACC CAA CGA
#MH985853.1_NZW2_ND4            ... ... ... ... ... ... ... ... ... ... ... ... ... ... ... ... ... ... ... ... ... ... ... ... ... ...
#MN296708_YW_ND4                ... ... ... ... ... ... ... ... ... ... ... ... ... ... ... ... ... ... ... ... ... ... ... ... ... ...
#AJ001588.1_NZW1_ND4            ... ... ... ... ... ... ... ... ... ... ... ... ... ... ... ... ... ... ... ... ... ... ... ... ... ...
#MN953621.1_CR_ND4              ... ... ... ... ... ... ... ... ... ... ... ... ... ... ... ... ... ... ... ... ... ... ... ... ... ...
```

Genetic characteristics of complete mtDNA genome sequence of Indonesian Local rabbit (*Oryctolagus cuniculus*) (2023)
by: A. Setiaji, D. A. Lestari, N. S. Pandupuspitasari, I. Agusetyaningsih, F. A. Khan

```
[                                111 111 111 111 111 111 111 111 111 111 111 111 111 111 111 111 111 111 111 111 111 111 111 111 111 111 ]
[                                222 222 222 222 222 222 222 222 222 222 222 222 222 222 222 222 222 333 333 333 333 333 333 333 333 333 ]
[                                455 555 555 556 666 666 666 777 777 777 788 888 888 889 999 999 999 000 000 000 011 111 111 112 222 222 ]
[                                901 234 567 890 123 456 789 012 345 678 901 234 567 890 123 456 789 012 345 678 901 234 567 890 123 456 ]
#Indonesian_Local_rabbit_ND4     GGC AAA TTC ACG TAT CAC ACA AAC AAC ATT TCC CCT ACA TTC ACC CGA GAA AAT ACT CTT ATA GTA CTT CAC CTA GCC
#MH985853.1_NZW2_ND4             ... ... ... ... ... ... ... ... ... ... ... ... ... ... ... ... ... ... ... ... ... ... ... ... ... ...
#MN296708_YW_ND4                 ... ... ... ... ... ... ... ... ... ... ... ... ... ... ... ... ... ... ... ... ... ... ... ... ... ...
#AJ001588.1_NZW1_ND4             ... ... ... ... ... ... ... ... ... ... ... ... ... ... ... ... ... ... ... ... ... ... ... ... ... ...
#MN953621.1_CR_ND4               ... ... ... ... ... ... ... ... ... ... ... ... ... ... ... ... ... ... ... ... ... ... ... ... ... ...

[                                111 111 111 111 111 111 111 111 111 111 111 111 111 111 111 111 111 1]
[                                333 333 333 333 333 333 333 333 333 333 333 333 333 333 333 333 333 3]
[                                222 333 333 333 344 444 444 445 555 555 555 666 666 666 677 777 777 7]
[                                789 012 345 678 901 234 567 890 123 456 789 012 345 678 901 234 567 8]
#Indonesian_Local_rabbit_ND4     CCA CTT CTT CTA CTA TCA ATT AGC CCA AAA ATC ATC TTA GGC CCA ATA TTC T
#MH985853.1_NZW2_ND4             ... ... ... ... ... ... ... ... ... ... ... ... ... ... .T. ... ... .
#MN296708_YW_ND4                 ... ... ... ... ... ... ... ... ... ... ... ... ... ... ... ... ... .
#AJ001588.1_NZW1_ND4             ... ... ... ... ... ... ... ... ... ... ... ... ... ... .T. ... ... .
#MN953621.1_CR_ND4               ... ... ... ... ... ... ... ... ... ... ... ... ... ... ... ... ... .
```

```
#MEGA
ND4L rabbit alignment

[                                            111 111 111 122 222 222 223 333 333 333 444 444 444 455 555 555 556 666 666 666 777 777 777 ]
[                                123 456 789 012 345 678 901 234 567 890 123 456 789 012 345 678 901 234 567 890 123 456 789 012 345 678 ]
#Indonesian_Local_rabbit_ND4L    ATG CCT TCA ATC TAT ATT AAT ATC TTC CTA GCA TTT ATC CTA GCT CTT CTA GGC ATA CTA GTC TAT CGA TCC CAT CTA
#MH985853.1_NZW2_ND4L            ... ... ... ... ... ... ... ... ... ... ... ... ... ... ... ... ... ... ... ... ... ... ... ... ... ...
#MN296708_YW_ND4L                ... ... ... ... ... ... ... ... ... ... ... ... ... ... ... ... ... ... ... ... ... ... ... ... ... ...
#AJ001588.1_NZW1_ND4L            ... ... ... ... ... ... ... ... ... ... ... ... ... ... ... ... ... ... ... ... ... ... ... ... ... ...
#MN953621.1_CR_ND4L              ... ... ... ... ... ... ... ... ... ... ... ... ... ... ... ... ... ... ... ... ... ... ... ... ... ...

[                                                            111 111 111 111 111 111 111 111 111 111 111 111 111 111 111 111 111 111 111 ]
[                                788 888 888 889 999 999 999 000 000 000 011 111 111 112 222 222 222 333 333 333 344 444 444 445 555 555 ]
[                                901 234 567 890 123 456 789 012 345 678 901 234 567 890 123 456 789 012 345 678 901 234 567 890 123 456 ]
#Indonesian_Local_rabbit_ND4L    ATA TCA TCC CTT CTA TGC CTA GAA GGC ATA ATA CTC TCA CTA TTT GTT CTA ATT ACA TTA ACA GCC CTT AAT ACC CAC
#MH985853.1_NZW2_ND4L            ... ... ... ... ... ... ... ... ... ... ... ... ... ... ... ... ... ... ... ... ... ... ... ... ... ...
#MN296708_YW_ND4L                ... ... ... ... ... ... ... ... ... ... ... ... ... ... ... ... ... ... ... ... ... ... ... ... ... ...
#AJ001588.1_NZW1_ND4L            ... ... ... ... ... ... ... ... ... ... ... ... ... ... ... ... ... ... ... ... ... ... ... ... ... ...
#MN953621.1_CR_ND4L              ... ... ... ... ... ... ... ... ... ... ... ... ... ... ... ... ... ... ... ... ... ... ... ... ... ...

[                                111 111 111 111 111 111 111 111 111 111 111 111 111 111 122 222 222 222 222 222 222 222 222 222 222 222 ]
[                                555 666 666 666 677 777 777 778 888 888 888 999 999 999 900 000 000 001 111 111 111 222 222 222 233 333 ]
[                                789 012 345 678 901 234 567 890 123 456 789 012 345 678 901 234 567 890 123 456 789 012 345 678 901 234 ]
#Indonesian_Local_rabbit_ND4L    TTC ACG CTA TCA TTT ATA TTT CCA ATC ATC CTA CTA GTA TTC GCA GCC TGT GAA GCA GCA GTT GGA CTA GCT CTT CTA
#MH985853.1_NZW2_ND4L            ... ..A ... ... ... ... ... ... ... ... ... ... ... ... ... ... ... ... ... ... ... ... ... ... ... ...
#MN296708_YW_ND4L                ... ..A ... ... ... ... ... ... ... ... ... ... ... ... ... ... ... ... ... ... ... ... ... ... ... ...
#AJ001588.1_NZW1_ND4L            ... ..A ... ... ... ... ... ... ... ... ... ... ... ... ... ... ... ... ... ... ... ... ... ... ... ...
#MN953621.1_CR_ND4L              ... ..A ... ... ... ... ... ... ... ... ... ... ... ... ... ... ... ... ... ... ... ... ... ... ... ...

[                                222 222 222 222 222 222 222 222 222 222 222 222 222 222 222 222 222 222 222 222 222 ]
[                                333 334 444 444 444 555 555 555 566 666 666 667 777 777 777 888 888 888 899 999 999 ]
[                                567 890 123 456 789 012 345 678 901 234 567 890 123 456 789 012 345 678 901 234 567 ]
#Indonesian_Local_rabbit_ND4L    GTT ATA GTC TCC AAC ACT TAT GGA ATA GAC TAT GTA CAA AAC CTA AAT CTT CTA CAA TGC TAA
#MH985853.1_NZW2_ND4L            ... ... ... ... ... ... ... ... ... ... ... ... ... ... ... ... ... ... ... ... ...
#MN296708_YW_ND4L                ... ... ... ... ... ... ... ... ... ... ... ... ... ... ... ... ... ... ... ... ...
#AJ001588.1_NZW1_ND4L            ... ... ... ... ... ... ... ... ... ... ... ... ... ... ... ... ... ... ... ... ...
#MN953621.1_CR_ND4L              ... ... ... ... ... ... ... ... ... ... ... ... ... ... ... ... ... ... ... ... ...
```

Genetic characteristics of complete mtDNA genome sequence of Indonesian Local rabbit (*Oryctolagus cuniculus*) (2023)
by: A. Setiaji, D. A. Lestari, N. S. Pandupuspitasari, I. Agusetyaningsih, F. A. Khan

```
#MEGA
ND5 rabbit alignment

[                                                   111 111 111 122 222 222 223 333 333 333 444 444 444 455 555 555 556 666 666 666 777 777 777 ]
[                                       123 456 789 012 345 678 901 234 567 890 123 456 789 012 345 678 901 234 567 890 123 456 789 012 345 678 ]
#Indonesian_Local_rabbit_ND5            ATT AAC TTA TTC TCC ACC TCT GTA GCT GTA TCA ATT ATT ATT TTA GTC CTC CCT ATT GTG GCC TCT TTC ACT AAC ATT
#MH985853.1_NZW2_ND5                    ... ... ... ... ... ... ... ... ... ... ... ... ... ... ... ... ... ... ... ... ... ... ... ... ... ...
#MN296708_YW_ND5                        ... ... ... ... ... ... ... ... ... ... ... ... ... ... ... ... ... ... ... ... ... ... ... ... ... ...
#AJ001588.1_NZW1_ND5                    ... ... ... ... ... ... ... ... ... ... ... ... ... ... ... ... ... ... ... ... ... ... ... ... ... ...
#MN953621.1_CR_ND5                      ... ... ... ... ... ... ... ... ... ... ... ... ... ... ... ... ... ... ... ... ... ... ... ... ... ...

[                                                                   111 111 111 111 111 111 111 111 111 111 111 111 111 111 111 111 111 111 111 ]
[                                       788 888 888 889 999 999 999 000 000 000 011 111 111 112 222 222 222 333 333 333 344 444 444 445 555 555 ]
[                                       901 234 567 890 123 456 789 012 345 678 901 234 567 890 123 456 789 012 345 678 901 234 567 890 123 456 ]
#Indonesian_Local_rabbit_ND5            TTT AAT AGT CCT AAC TAC CCT CAC TAC GTA AAA ACT TCA GTA TCT TAT GCA TTC ACT ATC AGT CTC ATT CCA ACA TTA
#MH985853.1_NZW2_ND5                    ... ... ... ... ... ... ... ... ... ... ... ... ... ... ... ... ... ... ... ... ... ... ... ... ... ...
#MN296708_YW_ND5                        ... ... ... ... ... ... ... ... ... ... ... ... ... ... ... ... ... ... ... ... ... ... ... ... ... ...
#AJ001588.1_NZW1_ND5                    ... ... ... ... ... ... ... ... ... ... ... ... ... ... ... ... ... ... ... ... ... ... ... ... ... ...
#MN953621.1_CR_ND5                      ... ... ... ... ... ... ... ... ... ... ... ... ... ... ... ... ... ... ... ... ... ... ... ... ... ...

[                                       111 111 111 111 111 111 111 111 111 111 111 111 111 111 122 222 222 222 222 222 222 222 222 222 222 222 ]
[                                       555 666 666 666 677 777 777 778 888 888 888 999 999 999 900 000 000 001 111 111 111 222 222 222 233 333 ]
[                                       789 012 345 678 901 234 567 890 123 456 789 012 345 678 901 234 567 890 123 456 789 012 345 678 901 234 ]
#Indonesian_Local_rabbit_ND5            ATT TTC ATC GCT ACT AGT CAA GAA ATA ATA CTA TCC AAC TGA CAC TGA ATA ACA ATT CAC ACC TTA AAA TTA ACA ACC
#MH985853.1_NZW2_ND5                    ... ... ... ... ... ... ... ... ... ... G.. ... ... ... ... ... ... ... ... ... ... ... ... ... ... ...
#MN296708_YW_ND5                        ... ... ... ... ... ... ... ... ... ... G.. ... ... ... ... ... ... ... ... ... ... ... ... ... ... ...
#AJ001588.1_NZW1_ND5                    ... ... ... ... ... ... ... ... ... ... G.. ... ... ... ... ... ... ... ... ... ... ... ... ... ... ...
#MN953621.1_CR_ND5                      ... ... ... ... ... ... ... ... ... ... G.. ... ... ... ... ... ... ... ... ... ... ... ... ... ... ...

[                                       222 222 222 222 222 222 222 222 222 222 222 222 222 222 222 222 222 222 222 222 222 223 333 333 333 333 ]
[                                       333 334 444 444 444 555 555 555 566 666 666 667 777 777 777 888 888 888 899 999 999 990 000 000 000 111 ]
[                                       567 890 123 456 789 012 345 678 901 234 567 890 123 456 789 012 345 678 901 234 567 890 123 456 789 012 ]
#Indonesian_Local_rabbit_ND5            AGC TTT AAA TTA GAT TAC TTC TCT ATA CTA TTC ACC CCA ATT GCA CTA TTT GTT ACA TGG TCT ATC ATA GAA TTT TCC
#MH985853.1_NZW2_ND5                    ... ... ... ... ... ... ... ... ... ... ... ... ... ... ... ... ... ... ... ... ... ... ... ... ... ...
#MN296708_YW_ND5                        ... ... ... ... ... ... ... ... ... ... ... ... ... ... ... ... ... ... ... ... ... ... ... ... ... ...
#AJ001588.1_NZW1_ND5                    ... ... ... ... ... ... ... ... ... ... ... ... ... ... ... ... ... ... ... ... ... ... ... ... ... ...
#MN953621.1_CR_ND5                      ... ... ... ... ... ... ... ... ... ... ... ... ... ... ... ... ... ... ... ... ... ... ... ... ... ...
```

Genetic characteristics of complete mtDNA genome sequence of Indonesian Local rabbit (*Oryctolagus cuniculus*) (2023)
by: A. Setiaji, D. A. Lestari, N. S. Pandupuspitasari, I. Agusetyaningsih, F. A. Khan

```
[                                333 333 333 333 333 333 333 333 333 333 333 333 333 333 333 333 333 333 333 333 333 333 333 333 333 333 ]
[                                111 111 122 222 222 223 333 333 333 444 444 444 455 555 555 556 666 666 666 777 777 777 788 888 888 889 ]
[                                345 678 901 234 567 890 123 456 789 012 345 678 901 234 567 890 123 456 789 012 345 678 901 234 567 890 ]
#Indonesian_Local_rabbit_ND5     ATA TGA TAC ATA CAC TCA GAC CCT AAA ATC AAC CAA TTC TTT AAA TAC CTA CTC ATA TTT CTT ATT ACT ATA CTA ATT
#MH985853.1_NZW2_ND5             ... ... ..T ... ... ... ... ... ... ... ... ... ... ... ... ... ... ... ... ... ... ... ... ... ... ...
#MN296708_YW_ND5                 ... ... ..T ... ... ... ... ... ... ... ... ... ... ... ... ... ... ... ... ... ... ... ... ... ... ...
#AJ001588.1_NZW1_ND5             ... ... ..T ... ... ... ... ... ... ... ... ... ... ... ... ... ... ... ... ... ... ... ... ... ... ...
#MN953621.1_CR_ND5               ... ... ..T ... ... ... ... ... ... ... ... ... ... ... ... ... ... ... ... ... ... ... ... ... ... ...

[                                333 333 333 444 444 444 444 444 444 444 444 444 444 444 444 444 444 444 444 444 444 444 444 444 444 444 ]
[                                999 999 999 000 000 000 011 111 111 112 222 222 222 333 333 333 344 444 444 445 555 555 555 666 666 666 ]
[                                123 456 789 012 345 678 901 234 567 890 123 456 789 012 345 678 901 234 567 890 123 456 789 012 345 678 ]
#Indonesian_Local_rabbit_ND5     TTA GTC ACC GCT AAT AAT ATA TTT CAG CTA TTT ATT GGA TGG GAG GGA GTG GGA ATC ATA TCA TTT CTC CTC ATC GGC
#MH985853.1_NZW2_ND5             ... ... ... ... ... ... ... ... ... ... ... ... ... ... ... ... ... ... ... ... ... ... ... ... ... ...
#MN296708_YW_ND5                 ... ... ... ... ... ... ... ... ... ... ... ... ... ... ... ... ... ... ... ... ... ... ... ... ... ...
#AJ001588.1_NZW1_ND5             ... ... ... ... ... ... ... ... ... ... ... ... ... ... ... ... ... ... ... ... ... ... ... ... ... ...
#MN953621.1_CR_ND5               ... ... ... ... ... ... ... ... ... ... ... ... ... ... ... ... ... ... ... ... ... ... ... ... ... ...

[                                444 444 444 444 444 444 444 444 444 444 455 555 555 555 555 555 555 555 555 555 555 555 555 555 555 555 ]
[                                677 777 777 778 888 888 888 999 999 999 900 000 000 001 111 111 111 222 222 222 233 333 333 334 444 444 ]
[                                901 234 567 890 123 456 789 012 345 678 901 234 567 890 123 456 789 012 345 678 901 234 567 890 123 456 ]
#Indonesian_Local_rabbit_ND5     TGA TGA CAC GGG CGA ACA GAT GCC AAT ACT GCA GCC TTA CAA GCA ATC TTA TAC AAC CGC ATT GGA GAT ATT GGC TTT
#MH985853.1_NZW2_ND5             ... ... ... ... ... ... ... ... ... ... ... ... ... ... ... ... ... ... ... ... ... ... ... ... ... ...
#MN296708_YW_ND5                 ... ... ... ... ... ... ... ... ... ... ... ... ... ... ... ... ... ... ... ... ... ... ... ... ... ...
#AJ001588.1_NZW1_ND5             ... ... ... ... ... ... ... ... ... ... ... ... ... ... ... ... ... ... ... ... ... ... ... ... ... ...
#MN953621.1_CR_ND5               ... ... ... ... ... ... ... ... ... ... ... ... ... ... ... ... ... ... ... ... ... ... ... ... ... ...

[                                555 555 555 555 555 555 555 555 555 555 555 555 555 555 555 555 555 556 666 666 666 666 666 666 666 666 ]
[                                444 555 555 555 566 666 666 667 777 777 777 888 888 888 899 999 999 990 000 000 000 111 111 111 122 222 ]
[                                789 012 345 678 901 234 567 890 123 456 789 012 345 678 901 234 567 890 123 456 789 012 345 678 901 234 ]
#Indonesian_Local_rabbit_ND5     ATT ATA GCC TTA GCC TGA TTT GCT ATT AAC CTC AAC ACA TGA GAA CTC CAA CAA ATC TTT ATT TTA GAC AAT AAC ATT
#MH985853.1_NZW2_ND5             ... ... ... ... ... ... ... ... ... ... ... ... ... ... ... ... ... ... ... ... ... ... ... ... ... ...
#MN296708_YW_ND5                 ... ... ... ... ... ... ... ... ... ... ... ... ... ... ... ... ... ... ... ... ... ... ... ... ... ...
#AJ001588.1_NZW1_ND5             ... ... ... ... ... ... ... ... ... ... ... ... ... ... ... ... ... ... ... ... ... ... ... ... ... ...
#MN953621.1_CR_ND5               ... ... ... ... ... ... ... ... ... ... ... ... ... ... ... ... ... ... ... ... ... ... ... ... ... ...
```

Genetic characteristics of complete mtDNA genome sequence of Indonesian Local rabbit (*Oryctolagus cuniculus*) (2023)
by: A. Setiaji, D. A. Lestari, N. S. Pandupuspitasari, I. Agusetyaningsih, F. A. Khan

```
[                                666 666 666 666 666 666 666 666 666 666 666 666 666 666 666 666 666 666 666 666 666 666 666 666 666 777 ]
[                                222 223 333 333 333 444 444 444 455 555 555 556 666 666 666 777 777 777 788 888 888 889 999 999 999 000 ]
[                                567 890 123 456 789 012 345 678 901 234 567 890 123 456 789 012 345 678 901 234 567 890 123 456 789 012 ]
#Indonesian_Local_rabbit_ND5     ACC ATC CTC CCA CTG ATA GGA TTA ATT CTT GCC GCC ACG GGC AAA TCC GCA CAA TTT GGT CTA CAC CCA TGA CTC CCA
#MH985853.1_NZW2_ND5             ... ... ... ... ... ... ... ... ... ... ... ... ... ... ... ... ... ... ... ... ... ... ... ... ... ...
#MN296708_YW_ND5                 ... ... ... ... ... ... ... ... ... ... ... ... ... ... ... ... ... ... ... ... ... ... ... ... ... ...
#AJ001588.1_NZW1_ND5             ... ... ... ... ... ... ... ... ... ... ... ... ... ... ... ... ... ... ... ... ... ... ... ... ... ...
#MN953621.1_CR_ND5               ... ... ... ... ... ... ... ... ... ... ... ... ... ... ... ... ... ... ... ... ... ... ... ... ... ...

[                                777 777 777 777 777 777 777 777 777 777 777 777 777 777 777 777 777 777 777 777 777 777 777 777 777 777 ]
[                                000 000 011 111 111 112 222 222 222 333 333 333 344 444 444 445 555 555 555 666 666 666 677 777 777 778 ]
[                                345 678 901 234 567 890 123 456 789 012 345 678 901 234 567 890 123 456 789 012 345 678 901 234 567 890 ]
#Indonesian_Local_rabbit_ND5     TCA GCT ATA GAA GGC CCG ACG CCC GTA TCA GCC CTA CTT CAT TCT AGC ACA ATA GTA GTA GCT GGA GTT TTC CTC CTA
#MH985853.1_NZW2_ND5             ... ... ... ... ... ... ... ... ... ... ... ... ... ... ... ... ... ... ... ... ... ... ... ... ... ...
#MN296708_YW_ND5                 ... ... ... ... ... ... ... ... ... ... ... ... ... ... ... ... ... ... ... ... ... ... ... ... ... ...
#AJ001588.1_NZW1_ND5             ... ... ... ... ... ... ... ... ... ... ... ... ... ... ... ... ... ... ... ... ... ... ... ... ... ...
#MN953621.1_CR_ND5               ... ... ... ... ... ... ... ... ... ... ... ... ... ... ... ... ... ... ... ... ... ... ... ... ... ...

[                                777 777 777 777 777 777 788 888 888 888 888 888 888 888 888 888 888 888 888 888 888 888 888 888 888 888 ]
[                                888 888 888 999 999 999 900 000 000 001 111 111 111 222 222 222 233 333 333 334 444 444 444 555 555 555 ]
[                                123 456 789 012 345 678 901 234 567 890 123 456 789 012 345 678 901 234 567 890 123 456 789 012 345 678 ]
#Indonesian_Local_rabbit_ND5     ATC CGC TTC TAC CCC TTG CTA GAA AAT AAC AAA ACT GCC CAA ACA TTA ATC CTA TGC CTA GGC GCA ATC ACT ACC TTA
#MH985853.1_NZW2_ND5             ... ... ... ... ... ... ... ... ... ... ... ... ... ... ... ... ... ... ... ... ... ... ... ... ... ...
#MN296708_YW_ND5                 ... ... ... ... ... ... ... ... ... ... ... ... ... ... ... ... ... ... ... ... ... ... ... ... ... ...
#AJ001588.1_NZW1_ND5             ... ... ... ... ... ... ... ... ... ... ... ... ... ... ... ... ... ... ... ... ... ... ... ... ... ...
#MN953621.1_CR_ND5               ... ... ... ... ... ... ... ... ... ... ... ... ... ... ... ... ... ... ... ... ... ... ... ... ... ...

[                                888 888 888 888 888 888 888 888 888 888 888 888 888 889 999 999 999 999 999 999 999 999 999 999 999 999 ]
[                                566 666 666 667 777 777 777 888 888 888 899 999 999 990 000 000 000 111 111 111 122 222 222 223 333 333 ]
[                                901 234 567 890 123 456 789 012 345 678 901 234 567 890 123 456 789 012 345 678 901 234 567 890 123 456 ]
#Indonesian_Local_rabbit_ND5     TTC ACA GCC CTA TGT GCC CTC ACA CAA AAC GAT ATC AAA AAA ATT GTA GCC TTT TCC ACA TCA AGT CAA CTC GGA CTA
#MH985853.1_NZW2_ND5             ... ... ... ... ... ... ... ... ... ... ..C ... ... ... ... ... ... ... ... ... ... ... ... ... ... ...
#MN296708_YW_ND5                 ... ... ... ... ... ... ... ... ... ... ..C ... ... ... ... ... ... ... ... ... ... ... ... ... ... ...
#AJ001588.1_NZW1_ND5             ... ... ... ... ... ... ... ... ... ... ..C ... ... ... ... ... ... ... ... ... ... ... ... ... ... ...
#MN953621.1_CR_ND5               ... ... ... ... ... ... ... ... ... ... ..C ... ... ... ... ... ... ... ... ... ... ... ... ... ... ...
```

Genetic characteristics of complete mtDNA genome sequence of Indonesian Local rabbit (*Oryctolagus cuniculus*) (2023)
by: A. Setiaji, D. A. Lestari, N. S. Pandupuspitasari, I. Agusetyaningsih, F. A. Khan

```
[                                                                                                    111 111 111 111 111 ]
[                               999 999 999 999 999 999 999 999 999 999 999 999 999 999 999 999 999 999 999 999 999 000 000 000 000 000 ]
[                               333 444 444 444 455 555 555 556 666 666 666 777 777 777 788 888 888 889 999 999 999 000 000 000 011 111 ]
[                               789 012 345 678 901 234 567 890 123 456 789 012 345 678 901 234 567 890 123 456 789 012 345 678 901 234 ]
#Indonesian_Local_rabbit_ND5    ATA ATG GTC ACA ATC GGA ATT AAC CAA CCA CAC CTG GCA TTT CTC CAC ATC TGC ACC CAC GCC TTC TTC AAA GCT ATG
#MH985853.1_NZW2_ND5            ... ... ... ... ... ... ... ... ... ... ... ... ... ... ... ... ... ... ... ... ... ... ... ... ... ...
#MN296708_YW_ND5                ... ... ... ... ... ... ... ... ... ... ... ... ... ... ... ... ... ... ... ... ... ... ... ... ... ...
#AJ001588.1_NZW1_ND5            ... ... ... ... ... ... ... ... ... ... ... ... ... ... ... ... ... ... ... ... ... ... ... ... ... ...
#MN953621.1_CR_ND5              ... ... ... ... ... ... ... ... ... ... ... ... ... ... ... ... ... ... ... ... ... ... ... ... ... ...

[                               111 111 111 111 111 111 111 111 111 111 111 111 111 111 111 111 111 111 111 111 111 111 111 111 111 111 ]
[                               000 000 000 000 000 000 000 000 000 000 000 000 000 000 000 000 000 000 000 000 000 000 000 000 000 000 ]
[                               111 112 222 222 222 333 333 333 344 444 444 445 555 555 555 666 666 666 677 777 777 778 888 888 888 999 ]
[                               567 890 123 456 789 012 345 678 901 234 567 890 123 456 789 012 345 678 901 234 567 890 123 456 789 012 ]
#Indonesian_Local_rabbit_ND5    CTC TTT CTC TGC TCC GGC TCA ATT ATT CAC AGC CTC AAT GAT GAA CAG GAT ATC CGA AAG ATA GGC GGC CTA TAT AAG
#MH985853.1_NZW2_ND5            ... ... ... ... ... ... ... ... ... ... ... ... ... ... ... ... ... ... ... ... ... ... ... ... ... ...
#MN296708_YW_ND5                ... ... ... ... ... ... ... ... ... ... ... ... ... ... ... ... ... ... ... ... ... ... ... ... ... ...
#AJ001588.1_NZW1_ND5            ... ... ... ... ... ... ... ... ... ... ... ... ... ... ... ... ... ... ... ... ... ... ... ... ... ...
#MN953621.1_CR_ND5              ... ... ... ... ... ... ... ... ... ... ... ... ... ... ... ... ... ... ... ... ... ... ... ... ... ...

[                               111 111 111 111 111 111 111 111 111 111 111 111 111 111 111 111 111 111 111 111 111 111 111 111 111 111 ]
[                               000 000 011 111 111 111 111 111 111 111 111 111 111 111 111 111 111 111 111 111 111 111 111 111 111 111 ]
[                               999 999 900 000 000 001 111 111 111 222 222 222 233 333 333 334 444 444 444 555 555 555 566 666 666 667 ]
[                               345 678 901 234 567 890 123 456 789 012 345 678 901 234 567 890 123 456 789 012 345 678 901 234 567 890 ]
#Indonesian_Local_rabbit_ND5    ACT ATA CCC TTC ACA GCC TCA GCC CTC ACT ATT GGT AGT CTG GCC CTC ACA GGT ATA CCA TTC CTA ACA GGC TTT TAC
#MH985853.1_NZW2_ND5            ... ... ... ... ... ... ... ... ... ... ... ... ... ... ... ... ... ... ... ... ... ... ... ... ... ...
#MN296708_YW_ND5                ... ... ... ... ... ... ... ... ... ... ... ... ... ... ... ... ... ... ... ... ... ... ... ... ... ...
#AJ001588.1_NZW1_ND5            ... ... ... ... ... ... ... ... ... ... ... ... ... ... ... ... ... ... ... ... ... ... ... ... ... ...
#MN953621.1_CR_ND5              ... ... ... C.. ... ... ... ... ... ... ... ... ... ... ... ... ... ... ... ... ... ... ... ... ... ...

[                               111 111 111 111 111 111 111 111 111 111 111 111 111 111 111 111 111 111 111 111 111 111 111 111 111 111 ]
[                               111 111 111 111 111 111 111 111 111 112 222 222 222 222 222 222 222 222 222 222 222 222 222 222 222 222 ]
[                               777 777 777 888 888 888 899 999 999 990 000 000 000 111 111 111 122 222 222 223 333 333 333 444 444 444 ]
[                               123 456 789 012 345 678 901 234 567 890 123 456 789 012 345 678 901 234 567 890 123 456 789 012 345 678 ]
#Indonesian_Local_rabbit_ND5    TCA AAA GAT CTA ATT ATT GAA TCA GCA AAC ACG TCT AAT ACC AAC GCC TGA GCC CTT ATC ATC ACT CTT ATT GCC ACA
#MH985853.1_NZW2_ND5            ... ... ... ... ... ... ... ... ... ... ... ... ... ... ... ... ... ... ... ... ... ... ... ... ... ...
#MN296708_YW_ND5                ... ... ... ... ... ... ... ... ... ... ... ... ... ... ... ... ... ... ... ... ... ... ... ... ... ...
#AJ001588.1_NZW1_ND5            ... ... ... ... ... ... ... ... ... ... ... ... ... ... ... ... ... ... ... ... ... ... ... ... ... ...
#MN953621.1_CR_ND5              ... ... ... ... ... ... ... ... ... ... ... ... ... ... ... ... ... ... ... ... ... ... ... ... ... ...
```

Genetic characteristics of complete mtDNA genome sequence of Indonesian Local rabbit (*Oryctolagus cuniculus*) (2023)
by: A. Setiaji, D. A. Lestari, N. S. Pandupuspitasari, I. Agusetyaningsih, F. A. Khan

Genetic characteristics of complete mtDNA genome sequence of Indonesian Local rabbit (*Oryctolagus cuniculus*) (2023)
by: A. Setiaji, D. A. Lestari, N. S. Pandupuspitasari, I. Agusetyaningsih, F. A. Khan

```
[                                111 111 111 111 111 111 111 111 111 111 111 111 111 111 111 111 111 111 111 111 111 111 111 111 111 111 ]
[                                222 222 222 222 222 222 222 222 222 222 222 222 222 222 222 222 222 333 333 333 333 333 333 333 333 333 ]
[                                455 555 555 556 666 666 666 777 777 777 788 888 888 889 999 999 999 000 000 000 011 111 111 112 222 222 ]
[                                901 234 567 890 123 456 789 012 345 678 901 234 567 890 123 456 789 012 345 678 901 234 567 890 123 456 ]
#Indonesian_Local_rabbit_ND5     TCC CTA ACA GCT GTC TAT AGC ACT CGA ATT ATC TTC TTC GCC CTC CTG GGT CAA CCC CGT TAC CCA GCC CTT ATT GTT
#MH985853.1_NZW2_ND5             ... ... ... ... ... ... ... ... ... ... ... ... ... ... ... ... ... ... ... ... ... ... ... ... ... ...
#MN296708_YW_ND5                 ... ... ... ... ... ... ... ... ... ... ... ... ... ... ... ... ... ... ... ... ... ... ... ... ... ...
#AJ001588.1_NZW1_ND5             ... ... ... ... ... ... ... ... ... ... ... ... ... ... ... ... ... ... ... ... ... ... ... ... ... ...
#MN953621.1_CR_ND5               ... ... ... ... ... ... ... ... ... ... ... ... ... ... ... ... ... ... ... ... ... ... ... ... ... ...

[                                111 111 111 111 111 111 111 111 111 111 111 111 111 111 111 111 111 111 111 111 111 111 111 111 111 111 ]
[                                333 333 333 333 333 333 333 333 333 333 333 333 333 333 333 333 333 333 333 333 333 333 333 333 344 444 ]
[                                222 333 333 333 344 444 444 445 555 555 555 666 666 666 677 777 777 778 888 888 888 999 999 999 900 000 ]
[                                789 012 345 678 901 234 567 890 123 456 789 012 345 678 901 234 567 890 123 456 789 012 345 678 901 234 ]
#Indonesian_Local_rabbit_ND5     ATT AAC GAA AAT AAC CCT CTA CTC ATT AAC TCT ATT AAA CGC CTT GCA CTA GGA AGC ATT TTT GCA GGA TTC TTA ATT
#MH985853.1_NZW2_ND5             ... ... ... ... ... ... ... ... ... ... ... ... ... ... ... ... ... ... ... ... ... ... ... ... ... ...
#MN296708_YW_ND5                 ... ... ... ... ... ... ... ... ... ... ... ... ... ... ... ... ... ... ... ... ... ... ... ... ... ...
#AJ001588.1_NZW1_ND5             ... ... ... ... ... ... ... ... ... ... ... ... ... ... ... ... ... ... ... ... ... ... ... ... ... ...
#MN953621.1_CR_ND5               ... ... ... ... ... ... ... ... ... ... ... ... ... ... ... ... ... ... ... ... ... ... ... ... ... ...

[                                111 111 111 111 111 111 111 111 111 111 111 111 111 111 111 111 111 111 111 111 111 111 111 111 111 111 ]
[                                444 444 444 444 444 444 444 444 444 444 444 444 444 444 444 444 444 444 444 444 444 444 444 444 444 444 ]
[                                000 001 111 111 111 222 222 222 233 333 333 334 444 444 444 555 555 555 566 666 666 667 777 777 777 888 ]
[                                567 890 123 456 789 012 345 678 901 234 567 890 123 456 789 012 345 678 901 234 567 890 123 456 789 012 ]
#Indonesian_Local_rabbit_ND5     TCC AAC CTT ATT ACA CCA AAC AAT GTC CCT CAA ATA ACT ATA CCC CTA TAT ATA AAA ATA ACT GCT CTA TTT GTT ACC
#MH985853.1_NZW2_ND5             ... ... ... ... ... ... ... ... ... ... ... ... ... ... ... ... ... ... ... ... ... ... ... ... ... ...
#MN296708_YW_ND5                 ... ... ... ... ... ... ... ... ... ... ... ... ... ... ... ... ... ... ... ... ... ... ... ... ... ...
#AJ001588.1_NZW1_ND5             ... ... ... ... ... ... ... ... ... ... ... ... ... ... ... ... ... ... ... ... ... ... ... ... ... ...
#MN953621.1_CR_ND5               ... ... ... ... ... ... ... ... ... ... ... ... ... ... ... ... ... ... ... ... ... ... ... ... ... ...

[                                111 111 111 111 111 111 111 111 111 111 111 111 111 111 111 111 111 111 111 111 111 111 111 111 111 111 ]
[                                444 444 444 444 444 445 555 555 555 555 555 555 555 555 555 555 555 555 555 555 555 555 555 555 555 555 ]
[                                888 888 899 999 999 990 000 000 000 111 111 111 122 222 222 223 333 333 333 444 444 444 455 555 555 556 ]
[                                345 678 901 234 567 890 123 456 789 012 345 678 901 234 567 890 123 456 789 012 345 678 901 234 567 890 ]
#Indonesian_Local_rabbit_ND5     ATT ATA GGC TTT ACT ATT GCA ATA GAA CTT AAC CAA CTC AGC CTT AGT CTA AAA ATA ACT ACA CAA TCT CCG TAC TTT
#MH985853.1_NZW2_ND5             ... ... ... ... ... ... ... ... ... ... ... ... ... ... ... ... ... ... ... ... ... ... ... ... ... ...
#MN296708_YW_ND5                 ... ... ... ... ... ... ... ... ... ... ... ... ... ... ... ... ... ... ... ... ... ... ... ... ... ...
#AJ001588.1_NZW1_ND5             ... ... ... ... ... ... ... ... ... ... ... ... ... ... ... ... ... ... ... ... ... ... ... ... ... ...
#MN953621.1_CR_ND5               ... ... ... ... ... ... ... ... ... ... ... ... ... ... ... ... ... ... ... ... ... ... ... ... ... ...
```

Genetic characteristics of complete mtDNA genome sequence of Indonesian Local rabbit (*Oryctolagus cuniculus*) (2023)
by: A. Setiaji, D. A. Lestari, N. S. Pandupuspitasari, I. Agusetyaningsih, F. A. Khan

Genetic characteristics of complete mtDNA genome sequence of Indonesian Local rabbit (*Oryctolagus cuniculus*) (2023)
by: A. Setiaji, D. A. Lestari, N. S. Pandupuspitasari, I. Agusetyaningsih, F. A. Khan

```
[                                111 111 111 111 111 111 111 111 111 111 111 111 111 111 111 111 111 111 111 111 111 111 111 111 111 111 ]
[                                555 555 555 555 555 555 555 555 555 555 555 555 555 666 666 666 666 666 666 666 666 666 666 666 666 666 ]
[                                666 666 666 777 777 777 788 888 888 889 999 999 999 000 000 000 011 111 111 112 222 222 222 333 333 333 ]
[                                123 456 789 012 345 678 901 234 567 890 123 456 789 012 345 678 901 234 567 890 123 456 789 012 345 678 ]
#Indonesian_Local_rabbit_ND5     AAC TTC TCA AAC ATA CTA GGG TTC TTT CCA ATG ACA ATT CAT CGT ATT CTA CCC TAT CTA AAC CTT TCA GCT AGC CAA
#MH985853.1_NZW2_ND5             ... ... ... ... ... ... ... ... ... ... ... ... ... ... ... ... ... ... ... ... ... ... ... ... ... ...
#MN296708_YW_ND5                 ... ... ... ... ... ... ... ... ... ... ... ... ... ... ... ... ... ... ... ... ... ... ... ... ... ...
#AJ001588.1_NZW1_ND5             ... ... ... ... ... ... ... ... ... ... ... ... ... ... ... ... ... ... ... ... ... ... ... ... ... ...
#MN953621.1_CR_ND5               ... ... ... ... ... ... ... ... ... ... ... ... ... ... ... ... ... ... ... ... ... ... ... ... ... ...

[                                111 111 111 111 111 111 111 111 111 111 111 111 111 111 111 111 111 111 111 111 111 111 111 111 111 111 ]
[                                666 666 666 666 666 666 666 666 666 666 666 666 666 666 666 666 666 666 666 666 677 777 777 777 777 777 ]
[                                344 444 444 445 555 555 555 666 666 666 677 777 777 778 888 888 888 999 999 999 900 000 000 001 111 111 ]
[                                901 234 567 890 123 456 789 012 345 678 901 234 567 890 123 456 789 012 345 678 901 234 567 890 123 456 ]
#Indonesian_Local_rabbit_ND5     AAC ATA GCC ACA TTA CTC CTA GAT ATG ACC TGA ACT GAA AAA GCG ATC CCG AAA AAT ATC TCA GAC ATT CAA ATT TTC
#MH985853.1_NZW2_ND5             ... ... ... ... ... ... ... ... ... ... ... ... ... ... ... ... ... ... ... ... ... ... ... ... ... ...
#MN296708_YW_ND5                 ... ... ... ... ... ... ... ... ... ... ... ... ... ... ... ... ... ... ... ... ... ... ... ... ... ...
#AJ001588.1_NZW1_ND5             ... ... ... ... ... ... ... ... ... ... ... ... ... ... ... ... ... ... ... ... ... ... ... ... ... ...
#MN953621.1_CR_ND5               ... ... ... ... ... ... ... ... ... ... ... ... ... ... ... ... ... ... ... ... ... ... ... ... ... ...

[                                111 111 111 111 111 111 111 111 111 111 111 111 111 111 111 111 111 111 111 111 111 111 111 111 111 111 ]
[                                777 777 777 777 777 777 777 777 777 777 777 777 777 777 777 777 777 777 777 777 777 777 777 777 777 777 ]
[                                111 222 222 222 233 333 333 334 444 444 444 555 555 555 566 666 666 667 777 777 777 888 888 888 899 999 ]
[                                789 012 345 678 901 234 567 890 123 456 789 012 345 678 901 234 567 890 123 456 789 012 345 678 901 234 ]
#Indonesian_Local_rabbit_ND5     GCA TCA ACC TCC GTA TCC TCA CAA AAA GGC CTC ATT AAA CTC TAC TTC CTA TCT TTC TTA ATC TCT TTA CTC TTA GTA
#MH985853.1_NZW2_ND5             ... ... ... ... ... ... ... ... ... ... ... ... ... ... ... ... ... ... ... ... ... ... ... ... ... ...
#MN296708_YW_ND5                 ... ... ... ... ... ... ... ... ... ... ... ... ... ... ... ... ... ... ... ... ... ... ... ... ... ...
#AJ001588.1_NZW1_ND5             ... ... ... ... ... ... ... ... ... ... ... ... ... ... ... ... ... ... ... ... ... ... ... ... ... ...
#MN953621.1_CR_ND5               ... ... ... ... ... ... ... ... ... ... ... ... ... ... ... ... ... ... ... ... ... ... ... ... ... ...

[                                111 111 111 111 111 111 ]
[                                777 778 888 888 888 888 ]
[                                999 990 000 000 000 111 ]
[                                567 890 123 456 789 012 ]
#Indonesian_Local_rabbit_ND5     CTA TTT ATT TTA ACC TAG
#MH985853.1_NZW2_ND5             ... ... ... ... ... ...
#MN296708_YW_ND5                 ... ... ... ... ... ...
#AJ001588.1_NZW1_ND5             ... ... ... ... ... ...
#MN953621.1_CR_ND5               ... ... ... ... ... ...
```

Genetic characteristics of complete mtDNA genome sequence of Indonesian Local rabbit (*Oryctolagus cuniculus*) (2023)
by: A. Setiaji, D. A. Lestari, N. S. Pandupuspitasari, I. Agusetyaningsih, F. A. Khan

```
#MEGA
ND6 rabbit alignment

[                                           111 111 111 122 222 222 223 333 333 333 444 444 444 455 555 555 556 666 666 666 777 777 777 ]
[                               123 456 789 012 345 678 901 234 567 890 123 456 789 012 345 678 901 234 567 890 123 456 789 012 345 678 ]
#Indonesian_Local_rabbit_ND6    CCT AGC CCC ACG AGT GAT CTC AAT AAC AAT AAA AAT ACT AAC AAA TAA AGA TCA ACC CGC AAC AAC CAT CAA TCA ACT
#MH985853.1_NZW2_ND6            ... ... ... ... ... A.. ... ... ... ... ... ... ... ... ... ... ... ... ... ... ... ... ... ... ... ...
#MN296708_YW_ND6                ... ... ... ... ... A.. ... ... ... ... ... ... ... ... ... ... ... ... ... ... ... ... ... ... ... ...
#AJ001588.1_NZW1_ND6            ... ... ... ... ... A.. ... ... ... ... ... ... ... ... ... ... ... ... ... ... ... ... ... ... ... ...
#MN953621.1_CR_ND6              ... ... ... ... ... A.. ... ... ... ... ... ... ... ... ... ... ... ... ... ... ... ... ... ... ..R ...

[                                                           111 111 111 111 111 111 111 111 111 111 111 111 111 111 111 111 111 111 111 ]
[                               788 888 888 889 999 999 999 000 000 000 011 111 111 112 222 222 222 333 333 333 344 444 444 445 555 555 ]
[                               901 234 567 890 123 456 789 012 345 678 901 234 567 890 123 456 789 012 345 678 901 234 567 890 123 456 ]
#Indonesian_Local_rabbit_ND6    ACC ATA ACT ATA CAA CGC CGC CAC CCC TAT AGA ATC CTC CCG GAT TAA TCC TAC CTC GTC CCC TTC AAA AAC CAC CCA
#MH985853.1_NZW2_ND6            ... ... ... ... ... ... ... ... ... ... ... ... ... ... ... ... ... ... ... ... ... ... ... ... ... ...
#MN296708_YW_ND6                ... ... ... ... ... ... ... ... ... ... ... ... ... ... ... ... ... ... ... ... ... ... ... ... ... ...
#AJ001588.1_NZW1_ND6            ... ... ... ... ... ... ... ... ... ... ... ... ... ... ... ... ... ... ... ... ... ... ... ... ... ...
#MN953621.1_CR_ND6              ... ... ... ... ... ... ... ... ... ... ... ... ... ... ... ... ... ... ... ... ... ... ... ... ... ...

[                               111 111 111 111 111 111 111 111 111 111 111 111 111 111 122 222 222 222 222 222 222 222 222 222 222 222 ]
[                               555 666 666 666 677 777 777 778 888 888 888 999 999 999 900 000 000 001 111 111 111 222 222 222 233 333 ]
[                               789 012 345 678 901 234 567 890 123 456 789 012 345 678 901 234 567 890 123 456 789 012 345 678 901 234 ]
#Indonesian_Local_rabbit_ND6    ATC CCC CAT ATT TTT AAA ATC AAC CAC AAT CTC AAC CCC ATC ACT CAT CAC TAT ATA CAC AAC CAA ACC TAC CTC TAT
#MH985853.1_NZW2_ND6            ... ... ... ... ... ... ... ... ... ... ... ... ... ... ... ... ... ... ... ... ... ... ... ... ... ...
#MN296708_YW_ND6                ... ... ... ... ... ... ... ... ... ... ... ... ... ... ... ... ... ... ... ... ... ... ... ... ... ...
#AJ001588.1_NZW1_ND6            ... ... ... ... ... ... ... ... ... ... ... ... ... ... ... ... ... ... ... ... ... ... ... ... ... ...
#MN953621.1_CR_ND6              ... ... ... ... ... ... ... ... ... ... ... ... ... ... ... ... ... ... ... ... ... ... ... ... ... ...

[                               222 222 222 222 222 222 222 222 222 222 222 222 222 222 222 222 222 222 222 222 222 223 333 333 333 333 ]
[                               333 334 444 444 444 555 555 555 566 666 666 667 777 777 777 888 888 888 899 999 999 990 000 000 000 111 ]
[                               567 890 123 456 789 012 345 678 901 234 567 890 123 456 789 012 345 678 901 234 567 890 123 456 789 012 ]
#Indonesian_Local_rabbit_ND6    TAA CAC CCC TAA AAC GAA TAT ACC CAA AAT CAT AAC ATT CGA TCC TCA AGT CTC AGG ATA CTC CTC AGT TGC CAT CGC
#MH985853.1_NZW2_ND6            ... ... ... ... ... ... ... ... ... ... ... ... ... ... ... ... ... ... ... ... ... ... ... ... ... ...
#MN296708_YW_ND6                ... ... ... ... ... ... ... ... ... ... ... ... ... ... ... ... ... ... ... ... ... ... ... ... ... ...
#AJ001588.1_NZW1_ND6            ... ... ... ... ... ... ... ... ... ... ... ... ... ... ... ... ... ... ... ... ... ... ... ... ... ...
#MN953621.1_CR_ND6              ... ... ... ... ... ... ... ... ... ... ... ... ... ... ... ... ... ... ... ... ... ... ... ... ... ...
```

Genetic characteristics of complete mtDNA genome sequence of Indonesian Local rabbit (*Oryctolagus cuniculus*) (2023)
by: A. Setiaji, D. A. Lestari, N. S. Pandupuspitasari, I. Agusetyaningsih, F. A. Khan

```
[                                333 333 333 333 333 333 333 333 333 333 333 333 333 333 333 333 333 333 333 333 333 333 333 333 333 333 ]
[                                111 111 122 222 222 223 333 333 333 444 444 444 455 555 555 556 666 666 666 777 777 777 788 888 888 889 ]
[                                345 678 901 234 567 890 123 456 789 012 345 678 901 234 567 890 123 456 789 012 345 678 901 234 567 890 ]
#Indonesian_Local_rabbit_ND6     AGT AGT ATA CCC AAA AAC TAC CAA CAT CCC CCC TAA ATA AAT TAA AAA CAT CAT TAA ACC TAA AAA TGA ACC CCC GAA
#MH985853.1_NZW2_ND6             ... ... ... ... ... ... ... ... ... ... ... ... ... ... ... ... ... ... ... ... ... ... ... ... ... ...
#MN296708_YW_ND6                 ... ... ... ... ... ... ... ... ... ... ... ... ... ... ... ... ... ... ... ... ... ... ... ... ... ...
#AJ001588.1_NZW1_ND6             ... ... ... ... ... ... ... ... ... ... ... ... ... ... ... ... ... ... ... ... ... ... ... ... ... ...
#MN953621.1_CR_ND6               ... ... ... ... ... ... ... ... ... ... ... ... ... ... ... ... ... ... ... ... ... ... ... ... ... ...

[                                333 333 333 444 444 444 444 444 444 444 444 444 444 444 444 444 444 444 444 444 444 444 444 444 444 444 ]
[                                999 999 999 000 000 000 011 111 111 112 222 222 222 333 333 333 344 444 444 445 555 555 555 666 666 666 ]
[                                123 456 789 012 345 678 901 234 567 890 123 456 789 012 345 678 901 234 567 890 123 456 789 012 345 678 ]
#Indonesian_Local_rabbit_ND6     ACT TAA AAC AAT ACC ACA CCC GAC CCC ACC ACT AAC AAT CAA CCC AAG CCC TCC ATA AAT TGG TGA TGG CTT AGA AGA
#MH985853.1_NZW2_ND6             ... ... ... ... ... ... ... ... ... ... ... ... ... ... ... ... ... ... ... ... ... ... ... ... ... ...
#MN296708_YW_ND6                 ... ... ... ... ... ... ... ... ... ... ... ... ... ... ... ... ... ... ... ... ... ... ... ... ... ...
#AJ001588.1_NZW1_ND6             ... ... ... ... ... ... ... ... ... ... ... ... ... ... ... ... ... ... ... ... ... ... ... ... ... ...
#MN953621.1_CR_ND6               ... ... ... ... ... ... ... ... ... ... ... ... ... ... ... ... ... ... ... ... ... ... ... ... ... ...

[                                444 444 444 444 444 444 444 444 444 444 455 555 555 555 555 555 555 555 555 ]
[                                677 777 777 778 888 888 888 999 999 999 900 000 000 001 111 111 111 222 222 ]
[                                901 234 567 890 123 456 789 012 345 678 901 234 567 890 123 456 789 012 345 ]
#Indonesian_Local_rabbit_ND6     AAA CCC AAC AAA CCC CAT CAC AAA CAT TAC ACT TAA TAA AAA CAC TAC GTA TGT CAT
#MH985853.1_NZW2_ND6             ... ... ... ... ... ... ... ... ... ... ... ... ... ... ... ... ... ... ...
#MN296708_YW_ND6                 ... ... ... ... ... ... ... ... ... ... ... ... ... ... ... ... ... ... ...
#AJ001588.1_NZW1_ND6             ... ... ... ... ... ... ... ... ... ... ... ... ... ... ... ... ... ... ...
#MN953621.1_CR_ND6               ... ... ... ... ... ... ... ... ... ... ... ... ... ... ... ... ... ... ...
```

```
#MEGA
COX1 rabbit alignment

[                                            111 111 111 122 222 222 223 333 333 333 444 444 444 455 555 555 556 666 666 666 777 777 777 ]
[                                123 456 789 012 345 678 901 234 567 890 123 456 789 012 345 678 901 234 567 890 123 456 789 012 345 678 ]
#Indenesian_Local_rabbit_COX1    ATG TTC GTC AAT CGT TGA CTT TTC TCT ACC AAC CAC AAA GAC ATC GGC ACT CTT TAT CTC CTA TTT GGA GCT TGA GCT
#MH985853.1_NZW2_COX1            ... ... ... ... ... ... ... ... ... ... ... ... ... ... ... ... ... ... ... ... ... ... ... ... ... ...
#MN296708_YW_COX1                ... ... ... ... ... ... ... ... ... ... ... ... ... ... ... ... ... ... ... ... ... ... ... ... ... ...
#AJ001588.1_NZW1_COX1            ... ... ... ... ... ... ... ... ... ... ... ... ... ... ... ... ... ... ... ... ... ... ... ... ... ...
#MN953621_CR_COX1                ... ... ... ... ... ... ... ... ... ... ... ... ... ... ... ... ... ... ... ... ... ... ... ... ... ...

[                                                            111 111 111 111 111 111 111 111 111 111 111 111 111 111 111 111 111 111 111 ]
[                                788 888 888 889 999 999 999 000 000 000 011 111 111 112 222 222 222 333 333 333 344 444 444 445 555 555 ]
[                                901 234 567 890 123 456 789 012 345 678 901 234 567 890 123 456 789 012 345 678 901 234 567 890 123 456 ]
#Indenesian_Local_rabbit_COX1    GGG ATG GTG GGA ACA GCC CTC AGC CTG CTA ATT CGA GCA GAA TTA GGT CAG CCA GGG ACT CTA CTC GGG GAT GAT CAA
#MH985853.1_NZW2_COX1            ... ... ... ... ... ... ... ... ... ... ... ... ... ... ... ... ... ... ... ... ... ... ... ... ... ...
#MN296708_YW_COX1                ... ... ... ... ... ... ... ... ... ... ... ... ... ... ... ... ... ... ... ... ... ... ... ... ... ...
#AJ001588.1_NZW1_COX1            ... ... ... ... ... ... ... ... ... ... ... ... ... ... ... ... ... ... ... ... ... ... ... ... ... ...
#MN953621_CR_COX1                ... ... ... ... ... ... ... ... ... ... ... ... ... ... ... ... ... ... ... ... ... ... ... ... ... ...

[                                111 111 111 111 111 111 111 111 111 111 111 111 111 111 122 222 222 222 222 222 222 222 222 222 222 222 ]
[                                555 666 666 666 677 777 777 778 888 888 888 999 999 999 900 000 000 001 111 111 111 222 222 222 233 333 ]
[                                789 012 345 678 901 234 567 890 123 456 789 012 345 678 901 234 567 890 123 456 789 012 345 678 901 234 ]
#Indenesian_Local_rabbit_COX1    ATC TAT AAT GTA ATC GTC ACC GCA CAT GCC TTT GTA ATA ATC TTC TTT ATA GTC ATG CCT ATT ATA ATT GGA GGC TTC
#MH985853.1_NZW2_COX1            ... ... ... ... ... ... ... ... ... ... ... ... ... ... ... ... ... ... ... ... ... ... ... ... ... ...
#MN296708_YW_COX1                ... ... ... ... ... ... ... ... ... ... ... ... ... ... ... ... ... ... ... ... ... ... ... ... ... ...
#AJ001588.1_NZW1_COX1            ... ... ... ... ... ... ... ... ... ... ... ... ... ... ... ... ... ... ... ... ... ... ... ... ... ...
#MN953621_CR_COX1                ... ... ... ... ... ... ... ... ... ... ... ... ... ... ... ... ... ... ... ... ... ... ... ... ... ...

[                                222 222 222 222 222 222 222 222 222 222 222 222 222 222 222 222 222 222 222 222 222 223 333 333 333 333 ]
[                                333 334 444 444 444 555 555 555 566 666 666 667 777 777 777 888 888 888 899 999 999 990 000 000 000 111 ]
[                                567 890 123 456 789 012 345 678 901 234 567 890 123 456 789 012 345 678 901 234 567 890 123 456 789 012 ]
#Indenesian_Local_rabbit_COX1    GGG AAC TGG CTT GTC CCC CTG ATA ATT GGG GCT CCT GAC ATA GCC TTC CCC CGA ATA AAT AAT ATG AGC TTC TGA CTT
#MH985853.1_NZW2_COX1            ... ... ... ... ... ... ... ... ... ... ... ... ... ... ... ... ... ... ... ... ... ... ... ... ... ...
#MN296708_YW_COX1                ... ... ... ... ... ... ... ... ... ... ... ... ... ... ... ... ... ... ... ... ... ... ... ... ... ...
#AJ001588.1_NZW1_COX1            ... ... ... ... ... ... ... ... ... ... ... ... ... ... ... ... ... ... ... ... ... ... ... ... ... ...
#MN953621_CR_COX1                ... ... ... ... ... ... ... ... ... ... ... ... ... ... ... ... ... ... ... ... ... ... ... ... ... ...
```

Genetic characteristics of complete mtDNA genome sequence of Indonesian Local rabbit (*Oryctolagus cuniculus*) (2023)
by: A. Setiaji, D. A. Lestari, N. S. Pandupuspitasari, I. Agusetyaningsih, F. A. Khan

```
[                                 333 333 333 333 333 333 333 333 333 333 333 333 333 333 333 333 333 333 333 333 333 333 333 333 333 333 ]
[                                 111 111 122 222 222 223 333 333 333 444 444 444 455 555 555 556 666 666 666 777 777 777 788 888 888 889 ]
[                                 345 678 901 234 567 890 123 456 789 012 345 678 901 234 567 890 123 456 789 012 345 678 901 234 567 890 ]
#Indenesian_Local_rabbit_COX1     CTC CCC CCT TCA TTC CTT CTT CTA CTA GCC TCC TCA ATA GTA GAA GCT GGG GCG GGG ACT GGC TGA ACT GTT TAT CCA
#MH985853.1_NZW2_COX1             ... ... ... ... ... ... ... ... ... ... ... ... ... ... ... ... ... ... ... ... ... ... ... ... ... ...
#MN296708_YW_COX1                 ... ... ... ... ... ... ... ... ... ... ... ... ... ... ... ... ... ... ... ... ... ... ... ... ... ...
#AJ001588.1_NZW1_COX1             ... ... ... ... ... ... ... ... ... ... ... ... ... ... ... ... ... ... ... ... ... ... ... ... ... ...
#MN953621_CR_COX1                 ... ... ... ... ... ... ... ... ... ... ... ... ... ... ... ... ... ... ... ... ... ... ... ... ... ...

[                                 333 333 333 444 444 444 444 444 444 444 444 444 444 444 444 444 444 444 444 444 444 444 444 444 444 444 ]
[                                 999 999 999 000 000 000 011 111 111 112 222 222 222 333 333 333 344 444 444 445 555 555 555 666 666 666 ]
[                                 123 456 789 012 345 678 901 234 567 890 123 456 789 012 345 678 901 234 567 890 123 456 789 012 345 678 ]
#Indenesian_Local_rabbit_COX1     CCT CTA GCC GGT AAT CTT GCA CAT GCT GGA GCC TCA GTG GAT CTT ACT ATT TTC TCC CTT CAC TTA GCT GGA GTA TCA
#MH985853.1_NZW2_COX1             ... ... ... ... ... ... ... ... ... ... ... ... ... ... ... ... ... ... ... ... ... ... ... ... ... ...
#MN296708_YW_COX1                 ... ... ... ... ... ... ... ... ... ... ... ... ... ... ... ... ... ... ... ... ... ... ... ... ... ...
#AJ001588.1_NZW1_COX1             ... ... ... ... ... ... ... ... ... ... ... ... ... ... ... ... ... ... ... ... ... ... ... ... ... ...
#MN953621_CR_COX1                 ... ... ... ... ... ... ... ... ... ... ... ... ... ... ... ... ... ... ... ... ... ... ... ... ... ...

[                                 444 444 444 444 444 444 444 444 444 444 455 555 555 555 555 555 555 555 555 555 555 555 555 555 555 555 ]
[                                 677 777 777 778 888 888 888 999 999 999 900 000 000 001 111 111 111 222 222 222 233 333 333 334 444 444 ]
[                                 901 234 567 890 123 456 789 012 345 678 901 234 567 890 123 456 789 012 345 678 901 234 567 890 123 456 ]
#Indenesian_Local_rabbit_COX1     TCT ATT TTA GGG GCT ATT AAC TTT ATT ACA ACT ATT ATT AAT ATG AAA CCC CCT GCA ATA TCT CAA TAT CAA ACC CCC
#MH985853.1_NZW2_COX1             ... ... ... ... ... ... ... ... ... ... ... ... ... ... ... ... G.. ... ... ... ... ... ... ... ... ...
#MN296708_YW_COX1                 ... ... ... ... ... ... ... ... ... ... ... ... ... ... ... ... ... ... ... ... ... ... ... ... ... ...
#AJ001588.1_NZW1_COX1             ... ... ... ... ... ... ... ... ... ... ... ... ... ... ... ... G.. ... ... ... ... ... ... ... ... ...
#MN953621_CR_COX1                 ... ... ... ... ... ... ... ... ... ... ... ... ... ... ... ... ... ... ... ... ... ... ... ... ... ...

[                                 555 555 555 555 555 555 555 555 555 555 555 555 555 555 555 555 555 556 666 666 666 666 666 666 666 666 ]
[                                 444 555 555 555 566 666 666 667 777 777 777 888 888 888 899 999 999 990 000 000 000 111 111 111 122 222 ]
[                                 789 012 345 678 901 234 567 890 123 456 789 012 345 678 901 234 567 890 123 456 789 012 345 678 901 234 ]
#Indenesian_Local_rabbit_COX1     TTA TTC GTA TGA TCT GTT CTA ATC ACA GCC GTA CTT CTT CTT CTC TCT TTA CCA GTC CTA GCT GCT GGC ATT ACA ATG
#MH985853.1_NZW2_COX1             ... ... ... ... ... ... ... ... ... ... ... ... ... ... ... ... ... ... ... ... ... ... ... ... ... ...
#MN296708_YW_COX1                 ... ... ... ... ... ... ... ... ... ... ... ... ... ... ... ... ... ... ... ... ... ... ... ... ... ...
#AJ001588.1_NZW1_COX1             ... ... ... ... ... ... ... ... ... ... ... ... ... ... ... ... ... ... ... ... ... ... ... ... ... ...
#MN953621_CR_COX1                 ... ... ... ... ... ... ... .C. ... ... ... ... ... ... ... ... ... ... ... ... ... ... ... ... ... ...
```

Genetic characteristics of complete mtDNA genome sequence of Indonesian Local rabbit (*Oryctolagus cuniculus*) (2023)
by: A. Setiaji, D. A. Lestari, N. S. Pandupuspitasari, I. Agusetyaningsih, F. A. Khan

```
[                                666 666 666 666 666 666 666 666 666 666 666 666 666 666 666 666 666 666 666 666 666 666 666 666 666 777 ]
[                                222 223 333 333 333 444 444 444 455 555 555 556 666 666 666 777 777 777 788 888 888 889 999 999 999 000 ]
[                                567 890 123 456 789 012 345 678 901 234 567 890 123 456 789 012 345 678 901 234 567 890 123 456 789 012 ]
#Indenesian_Local_rabbit_COX1    CTT TTA ACA GAC CGA AAC TTA AAT ACA ACC TTC TTT GAT CCT GCA GGA GGA GGA GAC CCT ATC CTC TAC CAA CAC CTA
#MH985853.1_NZW2_COX1            ... ... ... ... ... ... ... ... ... ... ... ... ... ... ... ... ... ... ... ... ... ... ... ... ... ...
#MN296708_YW_COX1                ... ... ... ... ... ... ... ... ... ... ... ... ... ... ... ... ... ... ... ... ... ... ... ... ... ...
#AJ001588.1_NZW1_COX1            ... ... ... ... ... ... ... ... ... ... ... ... ... ... ... ... ... ... ... ... ... ... ... ... ... ...
#MN953621_CR_COX1                ... ... ... ... ... ... ... ... ... ... ... ... ... ... ... ... ... ... ... ... ... ... ... ... ... ...

[                                777 777 777 777 777 777 777 777 777 777 777 777 777 777 777 777 777 777 777 777 777 777 777 777 777 777 ]
[                                000 000 011 111 111 112 222 222 222 333 333 333 344 444 444 445 555 555 555 666 666 666 677 777 777 778 ]
[                                345 678 901 234 567 890 123 456 789 012 345 678 901 234 567 890 123 456 789 012 345 678 901 234 567 890 ]
#Indenesian_Local_rabbit_COX1    TTC TGA TTT TTC GGG CAC CCC GAA GTA TAT ATT CTT ATT CTT CCA GGA TTT GGA ATA ATT TCG CAC ATT GTG ACA TAC
#MH985853.1_NZW2_COX1            ... ... ... ... ... ... ... ... ... ... ... ... ... ... ... ... ... ... ... ... ... ... ... ... ... ...
#MN296708_YW_COX1                ... ... ... ... ... ... ... ... ... ... ... ... ... ... ... ... ... ... ... ... ... ... ... ... ... ...
#AJ001588.1_NZW1_COX1            ... ... ... ... ... ... ... ... ... ... ... ... ... ... ... ... ... ... ... ... ... ... ... ... ... ...
#MN953621_CR_COX1                ... ... ... ... ... ... ... ... ... ... ... ... ... ... ... ... ... ... ... ... ... ... ... ... ... ...

[                                777 777 777 777 777 777 788 888 888 888 888 888 888 888 888 888 888 888 888 888 888 888 888 888 888 888 ]
[                                888 888 888 999 999 999 900 000 000 001 111 111 111 222 222 222 233 333 333 334 444 444 444 555 555 555 ]
[                                123 456 789 012 345 678 901 234 567 890 123 456 789 012 345 678 901 234 567 890 123 456 789 012 345 678 ]
#Indenesian_Local_rabbit_COX1    TAT TCC GGG AAA AAA GAG CCA TTT GGC TAT ATA GGA ATA GTA TGA GCT ATA ATA TCA ATT GGT TTC CTT GGA TTT ATC
#MH985853.1_NZW2_COX1            ... ... ... ... ... ... ... ... ... ... ... ... ... ... ... ... ... ... ... ... ... ... ... ... ... ...
#MN296708_YW_COX1                ... ... ... ... ... ... ... ... ... ... ... ... ... ... ... ... ... ... ... ... ... ... ... ... ... ...
#AJ001588.1_NZW1_COX1            ... ... ... ... ... ... ... ... ... ... ... ... ... ... ... ... ... ... ... ... ... ... ... ... ... ...
#MN953621_CR_COX1                ... ... ... ... ... ... ... ... ... ... ... ... ... ... ... ... ... ... ... ... ... ... ... ... ... ...

[                                888 888 888 888 888 888 888 888 888 888 888 888 888 889 999 999 999 999 999 999 999 999 999 999 999 999 ]
[                                566 666 666 667 777 777 777 888 888 888 899 999 999 990 000 000 000 111 111 111 122 222 222 223 333 333 ]
[                                901 234 567 890 123 456 789 012 345 678 901 234 567 890 123 456 789 012 345 678 901 234 567 890 123 456 ]
#Indenesian_Local_rabbit_COX1    GTA TGG GCC CAT CAT ATA TTT ACA GTA GGA ATA GAT GTA GAC ACA CGA GCC TAC TTT ACA TCA GCT ACC ATA ATC ATC
#MH985853.1_NZW2_COX1            ... ... ... ... ... ... ... ... ... ... ... ... ... ... ... ... ... ... ... ... ... ... ... ... ... ...
#MN296708_YW_COX1                ... ... ... ... ... ... ... ... ... ... ... ... ... ... ... ... ... ... ... ... ... ... ... ... ... ...
#AJ001588.1_NZW1_COX1            ... ... ... ... ... ... ... ... ... ... ... ... ... ... ... ... ... ... ... ... ... ... ... ... ... ...
#MN953621_CR_COX1                ... ... ... ... ... ... ... ... ... ... ... ... ... ... ... ... ... ... ... ... ... ... ... ... ... ...
```

Genetic characteristics of complete mtDNA genome sequence of Indonesian Local rabbit (*Oryctolagus cuniculus*) (2023)
by: A. Setiaji, D. A. Lestari, N. S. Pandupuspitasari, I. Agusetyaningsih, F. A. Khan

```
[                                                                                                                111 111 111 111 111 ]
[                                 999 999 999 999 999 999 999 999 999 999 999 999 999 999 999 999 999 999 999 999 999 000 000 000 000 000 ]
[                                 333 444 444 444 455 555 555 556 666 666 666 777 777 777 788 888 888 889 999 999 999 000 000 000 011 111 ]
[                                 789 012 345 678 901 234 567 890 123 456 789 012 345 678 901 234 567 890 123 456 789 012 345 678 901 234 ]
#Indenesian_Local_rabbit_COX1     GCT ATC CCT ACT GGA GTC AAA GTA TTT AGC TGG CTA GCA ACA CTG CAT GGC GGC AAC ATC AAA TGA TCC CCC GCT ATG
#MH985853.1_NZW2_COX1             ... ... ... ... ... ... ... ... ... ... ... ... ... ... ... ... ... ... ... ... ... ... ... ... ... ...
#MN296708_YW_COX1                 ... ... ... ... ... ... ... ... ... ... ... ... ... ... ... ... ... ... ... ... ... ... ... ... ... ...
#AJ001588.1_NZW1_COX1             ... ... ... ... ... ... ... ... ... ... ... ... ... ... ... ... ... ... ... ... ... ... ... ... ... ...
#MN953621_CR_COX1                 ... ... ... ... ... ... ... ... ... ... ... ... ... ... ... ... ... ... ... ... ... .K. ... ... ... ...

[                                 111 111 111 111 111 111 111 111 111 111 111 111 111 111 111 111 111 111 111 111 111 111 111 111 111 111 ]
[                                 000 000 000 000 000 000 000 000 000 000 000 000 000 000 000 000 000 000 000 000 000 000 000 000 000 000 ]
[                                 111 112 222 222 222 333 333 333 344 444 444 445 555 555 555 666 666 666 677 777 777 778 888 888 888 999 ]
[                                 567 890 123 456 789 012 345 678 901 234 567 890 123 456 789 012 345 678 901 234 567 890 123 456 789 012 ]
#Indenesian_Local_rabbit_COX1     CTA TGA GCT CTA GGC TTT ATT TTC CTA TTT ACA GTA GGC GGT CTT ACA GGA ATT GTG CTA GCC AAC TCC TCT TTA GAC
#MH985853.1_NZW2_COX1             ..C ... ... ... ... ... ... ... ... ... ... ... ... ... ... ... ... ... ... ... ... ... ... ... ... ...
#MN296708_YW_COX1                 ..C ... ... ... ... ... ... ... ... ... ... ... ... ... ... ... ... ... ... ... ... ... ... ... ... ...
#AJ001588.1_NZW1_COX1             ..C ... ... ... ... ... ... ... ... ... ... ... ... ... ... ... ... ... ... ... ... ... ... ... ... ...
#MN953621_CR_COX1                 ..C ... ... ... ... ... ... ... ... ... ... ... ... ... ... ... ... ... ... ... ... ... ... ... ... ...

[                                 111 111 111 111 111 111 111 111 111 111 111 111 111 111 111 111 111 111 111 111 111 111 111 111 111 111 ]
[                                 000 000 011 111 111 111 111 111 111 111 111 111 111 111 111 111 111 111 111 111 111 111 111 111 111 111 ]
[                                 999 999 900 000 000 001 111 111 111 222 222 222 233 333 333 334 444 444 444 555 555 555 566 666 666 667 ]
[                                 345 678 901 234 567 890 123 456 789 012 345 678 901 234 567 890 123 456 789 012 345 678 901 234 567 890 ]
#Indenesian_Local_rabbit_COX1     ATC GTA CTA CAT GAC ACC TAC TAT GTA GTA GCT CAC TTC CAC TAT GTC CTA TCT ATG GGG GCT GTA TTT GCT ATT ATA
#MH985853.1_NZW2_COX1             ... ... ... ... ... ... ... ... ... ... ... ... ... ... ... ... ... ... ... ... ... ... ... ... ... ...
#MN296708_YW_COX1                 ... ... ... ... ... ... ... ... ... ... ... ... ... ... ... ... ... ... ... ... ... ... ... ... ... ...
#AJ001588.1_NZW1_COX1             ... ... ... ... ... ... ... ... ... ... ... ... ... ... ... ... ... ... ... ... ... ... ... ... ... ...
#MN953621_CR_COX1                 ... ... ... ... ... ... ... ... ... ... ... ... ... ... ... ... ... ... ... ... ... ... ... ... ... ...

[                                 111 111 111 111 111 111 111 111 111 111 111 111 111 111 111 111 111 111 111 111 111 111 111 111 111 111 ]
[                                 111 111 111 111 111 111 111 111 111 112 222 222 222 222 222 222 222 222 222 222 222 222 222 222 222 222 ]
[                                 777 777 777 888 888 888 899 999 999 990 000 000 000 111 111 111 122 222 222 223 333 333 333 444 444 444 ]
[                                 123 456 789 012 345 678 901 234 567 890 123 456 789 012 345 678 901 234 567 890 123 456 789 012 345 678 ]
#Indenesian_Local_rabbit_COX1     GGA GGA TTT GCC CAT TGA TTC CCC CTA TTC TCA GGC TAT ACC CTT GAC CCA ACC TGA GCT AAA ATT CAC TTC ACT GTG
#MH985853.1_NZW2_COX1             ... ... ... ... ... ... ... ... ... ... ... ... ... ... ... ... ... ... ... ... ... ... ... ... ... ...
#MN296708_YW_COX1                 ... ... ... ... ... ... ... ... ... ... ... ... ... ... ... ... ... ... ... ... ... ... ... ... ... ...
#AJ001588.1_NZW1_COX1             ... ... ... ... ... ... ... ... ... ... ... ... ... ... ... ... ... ... ... ... ... ... ... ... ... ...
#MN953621_CR_COX1                 ... ... ... ... ... ... ... ... ... ... ... ... ... ... ... ... ... ... ... ... ... ... ... ... ... ...
```

Genetic characteristics of complete mtDNA genome sequence of Indonesian Local rabbit (*Oryctolagus cuniculus*) (2023)
by: A. Setiaji, D. A. Lestari, N. S. Pandupuspitasari, I. Agusetyaningsih, F. A. Khan

```
[                               111 111 111 111 111 111 111 111 111 111 111 111 111 111 111 111 111 111 111 111 111 111 111 111 111 111 ]
[                               222 222 222 222 222 222 222 222 222 222 222 222 222 222 222 222 222 333 333 333 333 333 333 333 333 333 ]
[                               455 555 555 556 666 666 666 777 777 777 788 888 888 889 999 999 999 000 000 000 011 111 111 112 222 222 ]
[                               901 234 567 890 123 456 789 012 345 678 901 234 567 890 123 456 789 012 345 678 901 234 567 890 123 456 ]
#Indenesian_Local_rabbit_COX1   ATA TTT GTA GGA GTC AAC TTA ACT TTC TTC CCT CAA CAT TTC CTT GGC CTC TCA GGT ATA CCC CGA CGG TAC TCA GAC
#MH985853.1_NZW2_COX1           ... ... ... ... ... ... ... ... ... ... ... ... ... ... ... ... ... ... ... ... ... ... ... ... ... ...
#MN296708_YW_COX1               ... ... ... ... ... ... ... ... ... ... ... ... ... ... ... ... ... ... ... ... ... ... ... ... ... ...
#AJ001588.1_NZW1_COX1           ... ... ... ... ... ... ... ... ... ... ... ... ... ... ... ... ... ... ... ... ... ... ... ... ... ...
#MN953621_CR_COX1               ... C.. ... ... ... ... ... ... ... ... ... ... ... ... ... ... ... ... ... ... ... ... ... ... ... ...

[                               111 111 111 111 111 111 111 111 111 111 111 111 111 111 111 111 111 111 111 111 111 111 111 111 111 111 ]
[                               333 333 333 333 333 333 333 333 333 333 333 333 333 333 333 333 333 333 333 333 333 333 333 333 344 444 ]
[                               222 333 333 333 344 444 444 445 555 555 555 666 666 666 677 777 777 778 888 888 888 999 999 999 900 000 ]
[                               789 012 345 678 901 234 567 890 123 456 789 012 345 678 901 234 567 890 123 456 789 012 345 678 901 234 ]
#Indenesian_Local_rabbit_COX1   TAC CCG GAC GCC TAT ACA ATA TGA AAT ACT GTT TCA TCA ATA GGC TCA TTC ATT TCC CTA ACT GCC GTA ATA GTA ATA
#MH985853.1_NZW2_COX1           ... ... ... ... ... ... ... ... ... ... ... ... ... ... ... ... ... ... ... ... ... ... ... ... ... ...
#MN296708_YW_COX1               ... ... ... ... ... ... ... ... ... ... ... ... ... ... ... ... ... ... ... ... ... ... ... ... ... ...
#AJ001588.1_NZW1_COX1           ... ... ... ... ... ... ... ... ... ... ... ... ... ... ... ... ... ... ... ... ... ... ... ... ... ...
#MN953621_CR_COX1               ... ... ... ... ... ... ... ... ... ... ... ... ... ... ... ... ... ... ... ... ... ... ... ... ... ...

[                               111 111 111 111 111 111 111 111 111 111 111 111 111 111 111 111 111 111 111 111 111 111 111 111 111 111 ]
[                               444 444 444 444 444 444 444 444 444 444 444 444 444 444 444 444 444 444 444 444 444 444 444 444 444 444 ]
[                               000 001 111 111 111 222 222 222 233 333 333 334 444 444 444 555 555 555 566 666 666 667 777 777 777 888 ]
[                               567 890 123 456 789 012 345 678 901 234 567 890 123 456 789 012 345 678 901 234 567 890 123 456 789 012 ]
#Indenesian_Local_rabbit_COX1   ATT TTT ATA ATC TGA GAA GCC TTC GCC TCA AAA CGA GAA GTA GAG ACT ATT GAA CTA ACA ACC ACA AAC CTA GAG TGA
#MH985853.1_NZW2_COX1           ... ... ... ... ... ... ... ... ... ... ... ... ... ... ... ... ... ... ... ... ... ... ... ... ..A ...
#MN296708_YW_COX1               ... ... ... ... ... ... ... ... ... ... ... ... ... ... ... ... ... ... ... ... ... ... ... ... ..A ...
#AJ001588.1_NZW1_COX1           ... ... ... ... ... ... ... ... ... ... ... ... ... ... ... ... ... ... ... ... ... ... ... ... ..A ...
#MN953621_CR_COX1               ... ... ... ... ... ... ... ... ... ... ... ... ... ... ... ... ... ... ... .T. ... ... ... ... ..A ...

[                               111 111 111 111 111 111 111 111 111 111 111 111 111 111 111 111 111 111 111 111 ]
[                               444 444 444 444 444 445 555 555 555 555 555 555 555 555 555 555 555 555 555 555 ]
[                               888 888 899 999 999 990 000 000 000 111 111 111 122 222 222 223 333 333 333 444 ]
[                               345 678 901 234 567 890 123 456 789 012 345 678 901 234 567 890 123 456 789 012 ]
#Indenesian_Local_rabbit_COX1   CTT CAT GGA TGC CCT CCC CCA TAC CAT ACA TTT GAA GAA CCC GCT TTC GTA AAA GCT TAG
#MH985853.1_NZW2_COX1           ... ... ... ... ... ... ... ... ... ... ... ... ... ... ... ... ... ... ... ...
#MN296708_YW_COX1               ... ... ... ... ... ... ... ... ... ... ... ... ... ... ... ... ... ... ... ...
#AJ001588.1_NZW1_COX1           ... ... ... ... ... ... ... ... ... ... ... ... ... ... ... ... ... ... ... ...
#MN953621_CR_COX1               ... ... ... ... ... ... ... ... ... ... ... ... ... ... ... ... ... ... ... ...
```

Genetic characteristics of complete mtDNA genome sequence of Indonesian Local rabbit (*Oryctolagus cuniculus*) (2023)
by: A. Setiaji, D. A. Lestari, N. S. Pandupuspitasari, I. Agusetyaningsih, F. A. Khan

```
#MEGA
COX2 rabbit alignment

[                                            111 111 111 122 222 222 223 333 333 333 444 444 444 455 555 555 556 666 666 666 777 777 777 ]
[                                123 456 789 012 345 678 901 234 567 890 123 456 789 012 345 678 901 234 567 890 123 456 789 012 345 678 ]
#Indonesian_Local_rabbit_COX2    ATG GCG TAC CCC TTC CAA CTG GGC TTT CAG GAT GCC TCA TCC CCT ATT ATA GAA GAA TTA CTT CAC TTT CAT GAC CAC
#MH985853_NZW2_COX2              ... ... ... ... ... ... ... ... ... ... ... ... ... ... ... ... ... ... ... ... ... ... ... ... ... ...
#MN296708_YW_COX2                ... ... ... ... ... ... ... ... ... ... ... ... ... ... ... ... ... ... ... ... ... ... ... ... ... ...
#AJ001588.1_NZW1_COX2            ... ... ... ... ... ... ... ... ... ... ... ... ... ... ... ... ... ... ... ... ... ... ... ... ... ...
#MN953621_CR_COX2                ... ... ... ... ... ... ... ... ... ... ... ... ... ... ... ... ... ... ... ... ... ... ... ... ... ...

[                                                            111 111 111 111 111 111 111 111 111 111 111 111 111 111 111 111 111 111 111 ]
[                                788 888 888 889 999 999 999 000 000 000 011 111 111 112 222 222 222 333 333 333 344 444 444 445 555 555 ]
[                                901 234 567 890 123 456 789 012 345 678 901 234 567 890 123 456 789 012 345 678 901 234 567 890 123 456 ]
#Indonesian_Local_rabbit_COX2    ACT CTC ATA ATC GTC TTT CTA ATC AGC TCC CTA GTC CTT TAT ATT ATT TCT CTT ATA TTA ACT ACA AAG CTC ACT CAC
#MH985853_NZW2_COX2              ... ... ... ... ... ... ... ... ... ... ... ... ... ... ... ... ... ... ... ... ... ... ... ... ... ...
#MN296708_YW_COX2                ... ... ... ... ... ... ... ... ... ... ... ... ... ... ... ... ... ... ... ... ... ... ... ... ... ...
#AJ001588.1_NZW1_COX2            ... ... ... ... ... ... ... ... ... ... ... ... ... ... ... ... ... ... ... ... ... ... ... ... ... ...
#MN953621_CR_COX2                ... ... ... ... ... ... ... ... ... ... ... ... ... ... ... ... ... ... ... ... ... ... ... ... ... ...

[                                111 111 111 111 111 111 111 111 111 111 111 111 111 111 122 222 222 222 222 222 222 222 222 222 222 222 ]
[                                555 666 666 666 677 777 777 778 888 888 888 999 999 999 900 000 000 001 111 111 111 222 222 222 233 333 ]
[                                789 012 345 678 901 234 567 890 123 456 789 012 345 678 901 234 567 890 123 456 789 012 345 678 901 234 ]
#Indonesian_Local_rabbit_COX2    ACA AGC ACA ATG GAT GCT CAG GAG GTA GAG ACA ATC TGA ACC ATC CTC CCG GCC ATT ATT CTT ATT TTA ATT GCG CTG
#MH985853_NZW2_COX2              ... ... ... ... ... ... ... ... ... ... ... ... ... ... ... ... ..A ... ... ... ... ... ... ... ... ...
#MN296708_YW_COX2                ... ... ... ... ... ... ... ... ... ... ... ... ... ... ... ... ..A ... ... ... ... ... ... ... ... ...
#AJ001588.1_NZW1_COX2            ... ... ... ... ... ... ... ... ... ... ... ... ... ... ... ... ..A ... ... ... ... ... ... ... ... ...
#MN953621_CR_COX2                ... ... ... ... ... ... ... ... ... ... ... ... ... ... ... ... ..A ... ... ... ... ... ... ... ... ...

[                                222 222 222 222 222 222 222 222 222 222 222 222 222 222 222 222 222 222 222 222 222 223 333 333 333 333 ]
[                                333 334 444 444 444 555 555 555 566 666 666 667 777 777 777 888 888 888 899 999 999 990 000 000 000 111 ]
[                                567 890 123 456 789 012 345 678 901 234 567 890 123 456 789 012 345 678 901 234 567 890 123 456 789 012 ]
#Indonesian_Local_rabbit_COX2    CCC TCC CTA CGA ATC CTA TAT ATA ATA GAT GAA ATC AAC AAC CCC TCT TTA ACA GTA AAA ACG ATA GGC CAC CAA TGA
#MH985853_NZW2_COX2              ... ... ... ... ... ... ... ... ... ... ... ... ... ... ... ... ... ... ... ... ... ... ... ... ... ...
#MN296708_YW_COX2                ... ... ... ... ... ... ... ... ... ... ... ... ... ... ... ... ... ... ... ... ... ... ... ... ... ...
#AJ001588.1_NZW1_COX2            ... ... ... ... ... ... ... ... ... ... ... ... ... ... ... ... ... ... ... ... ... ... ... ... ... ...
#MN953621_CR_COX2                ... ... ... ... ... ... ... ... ... ... ... ... ... ... ... ... ... ... ... ... ... ... ... ... ... ...
```

Genetic characteristics of complete mtDNA genome sequence of Indonesian Local rabbit (*Oryctolagus cuniculus*) (2023)
by: A. Setiaji, D. A. Lestari, N. S. Pandupuspitasari, I. Agusetyaningsih, F. A. Khan

```
[                                333 333 333 333 333 333 333 333 333 333 333 333 333 333 333 333 333 333 333 333 333 333 333 333 333 333 ]
[                                111 111 122 222 222 223 333 333 333 444 444 444 455 555 555 556 666 666 666 777 777 777 788 888 888 889 ]
[                                345 678 901 234 567 890 123 456 789 012 345 678 901 234 567 890 123 456 789 012 345 678 901 234 567 890 ]
#Indonesian_Local_rabbit_COX2    TAT TGA AGC TAT GAG TAC ACA GAC TAT GAG GAT TTA AAT TTT GAT TCT TAC ATG ATC CCA ACA TCA GAC CTG AAT CCA
#MH985853_NZW2_COX2              ... ... ... ... ... ... ... ... ... ... ... ... ... ... ... ... ... ... ... ... ... ... ... ... ... ...
#MN296708_YW_COX2                ... ... ... ... ... ... ... ... ... ... ... ... ... ... ... ... ... ... ... ... ... ... ... ... ... ...
#AJ001588.1_NZW1_COX2            ... ... ... ... ... ... ... ... ... ... ... ... ... ... ... ... ... ... ... ... ... ... ... ... ... ...
#MN953621_CR_COX2                ... ... ... ... ... ... ... ... ... ... ... ... ... ... ... ... ... ... ... ... ... ... ... ... ... ...

[                                333 333 333 444 444 444 444 444 444 444 444 444 444 444 444 444 444 444 444 444 444 444 444 444 444 444 ]
[                                999 999 999 000 000 000 011 111 111 112 222 222 222 333 333 333 344 444 444 445 555 555 555 666 666 666 ]
[                                123 456 789 012 345 678 901 234 567 890 123 456 789 012 345 678 901 234 567 890 123 456 789 012 345 678 ]
#Indonesian_Local_rabbit_COX2    GGT GAC CTA CGA CTA CTA GAA GTT GAC AAT CGA GTT GTA CTT CCC ATA GAA CTC CCA ATC CGC ATG CTA ATC TCC TCG
#MH985853_NZW2_COX2              ... ... ... ... ... ... ... ... ... ... ... ... ... ... ... ... ... ... ... ... ... ... ... ... ... ...
#MN296708_YW_COX2                ... ... ... ... ... ... ... ... ... ... ... ... ... ... ... ... ... ... ... ... ... ... ... ... ... ...
#AJ001588.1_NZW1_COX2            ... ... ... ... ... ... ... ... ... ... ... ... ... ... ... ... ... ... ... ... ... ... ... ... ... ...
#MN953621_CR_COX2                ... ... ... ... ... ... ... ... ... ... ... ... ... ... ... ... ... ... ... ... ... ... ... ... ... ...

[                                444 444 444 444 444 444 444 444 444 444 455 555 555 555 555 555 555 555 555 555 555 555 555 555 555 555 ]
[                                677 777 777 778 888 888 888 999 999 999 900 000 000 001 111 111 111 222 222 222 233 333 333 334 444 444 ]
[                                901 234 567 890 123 456 789 012 345 678 901 234 567 890 123 456 789 012 345 678 901 234 567 890 123 456 ]
#Indonesian_Local_rabbit_COX2    GAA GAC GTA CTT CAC TCA TGA GCC GTA CCA TCA CTA GGA CTA AAA ACA GAT GCC ATT CCC GGA CGC TTA AAT CAA GCC
#MH985853_NZW2_COX2              ... ... ... ... ... ... ... ... ... ... ... ... ... ... ... ... ... ... ... ... ... ... ... ... ... ...
#MN296708_YW_COX2                ... ... ... ... ... ... ... ... ... ... ... ... ... ... ... ... ... ... ... ... ... ... ... ... ... ...
#AJ001588.1_NZW1_COX2            ... ... ... ... ... ... ... ... ... ... ... ... ... ... ... ... ... ... ... ... ... ... ... ... ... ...
#MN953621_CR_COX2                ... ... ... ... ... ... ... ... ... ... ... ... ... ... ... ... ... ... ... ... ... ... ... ... ... ...

[                                555 555 555 555 555 555 555 555 555 555 555 555 555 555 555 555 555 556 666 666 666 666 666 666 666 666 ]
[                                444 555 555 555 566 666 666 667 777 777 777 888 888 888 899 999 999 990 000 000 000 111 111 111 122 222 ]
[                                789 012 345 678 901 234 567 890 123 456 789 012 345 678 901 234 567 890 123 456 789 012 345 678 901 234 ]
#Indonesian_Local_rabbit_COX2    ACA CTT ATC TCA ACT CGA CCA GGA CTT TTC TAT GGT CAG TGC TCA GAA ATT TGT GGC TCA AAC CAT AGC TTT ATA CCT
#MH985853_NZW2_COX2              ... ... ... ... ... ... ... ... ... ... ... ... ... ... ... ... ... ... ... ... ... ... ... ... ... ...
#MN296708_YW_COX2                ... ... ... ... ... ... ... ... ... ... ... ... ... ... ... ... ... ... ... ... ... ... ... ... ... ...
#AJ001588.1_NZW1_COX2            ... ... ... ... ... ... ... ... ... ... ... ... ... ... ... ... ... ... ... ... ... ... ... ... ... ...
#MN953621_CR_COX2                ... ... ... ... ... ... ... ... ... ... ... ... ... ... ... ... ... ... ... ... ... ... ... ... ... ...
```

```
[                                666 666 666 666 666 666 666 666 666 666 666 666 666 666 666 666 666 666 666 666 ]
[                                222 223 333 333 333 444 444 444 455 555 555 556 666 666 666 777 777 777 788 888 ]
[                                567 890 123 456 789 012 345 678 901 234 567 890 123 456 789 012 345 678 901 234 ]
#Indonesian_Local_rabbit_COX2    ATT GTC CTC GAA ATA GTT CCA CTT AAA CAC TTC GAA AAC TGA TCC CTA TCT ATG ATT TAG
#MH985853_NZW2_COX2              ... ... ... ... ... ... ... ... ... ... ... ... ... ... ... ... ... ... ... ...
#MN296708_YW_COX2                ... ... ... ... ... ... ... ... ... ... ... ... ... ... ... ... ... ... ... ...
#AJ001588.1_NZW1_COX2            ... ... ... ... ... ... ... ... ... ... ... ... ... ... ... ... ... ... ... ...
#MN953621_CR_COX2                ... ... ... ... ... A.. ... ... ... ... ... ... ... ... ... ... ... ... ... ...
```

Genetic characteristics of complete mtDNA genome sequence of Indonesian Local rabbit (*Oryctolagus cuniculus*) (2023)
by: A. Setiaji, D. A. Lestari, N. S. Pandupuspitasari, I. Agusetyaningsih, F. A. Khan

```
#MEGA
COX3 rabbit alignment

[                                             111 111 111 122 222 222 223 333 333 333 444 444 444 455 555 555 556 666 666 666 777 777 777 ]
[                                 123 456 789 012 345 678 901 234 567 890 123 456 789 012 345 678 901 234 567 890 123 456 789 012 345 678 ]
#Indonesian_Local_rabbit_COX3     ATG ACA CAC CAA ACT CAC GCT TAC CAT ATA GTC AAC CCA AGC CCC TGA CCA CTC ACC GGA GCC CTA TCT GCC CTT CTT
#MH985853.1_NZW2_COX3             ... ... ... ... ... ... ... ... ... ... ... ... ... ... ... ... ... ... ... ... ... ... ... ... ... ...
#MN296708_YW_COX3                 ... ... ... ... ... ... ... ... ... ... ... ... ... ... ... ... ... ... ... ... ... ... ... ... ... ...
#AJ001588.1_NZW1_COX3             ... ... ... ... ... ... ... ... ... ... ... ... ... ... ... ... ... ... ... ... ... ... ... ... ... ...
#MN953621_CR_COX3                 ... ... ... ... ... ... ... ... ... ... ... ... ... ... ... ... ... ... ... ... ... ... ... ... ... ...

[                                                             111 111 111 111 111 111 111 111 111 111 111 111 111 111 111 111 111 111 111 ]
[                                 788 888 888 889 999 999 999 000 000 000 011 111 111 112 222 222 222 333 333 333 344 444 444 445 555 555 ]
[                                 901 234 567 890 123 456 789 012 345 678 901 234 567 890 123 456 789 012 345 678 901 234 567 890 123 456 ]
#Indonesian_Local_rabbit_COX3     ATG ACA TCA GGC CTA GCC ATA TGA TTT CAT TTT AAC TCC CCC TCA CTT CTA TTA ATC GGC TTA GTA ACC AAC ACT CTC
#MH985853.1_NZW2_COX3             ... ... ... ... ... ... ... ... ... ... ... ... ... ... ... ... ... ... ... ... ... ... ... ... ... ...
#MN296708_YW_COX3                 ... ... ... ... ... ... ... ... ... ... ... ... ... ... ... ... ... ... ... ... ... ... ... ... ... ...
#AJ001588.1_NZW1_COX3             ... ... ... ... ... ... ... ... ... ... ... ... ... ... ... ... ... ... ... ... ... ... ... ... ... ...
#MN953621_CR_COX3                 ... ... ... ... ... ... ... ... ... ... ... ... ... ... ... ... ... ... ... ... ... ... ... ... ... ...

[                                 111 111 111 111 111 111 111 111 111 111 111 111 111 111 122 222 222 222 222 222 222 222 222 222 222 222 ]
[                                 555 666 666 666 677 777 777 778 888 888 888 999 999 999 900 000 000 001 111 111 111 222 222 222 233 333 ]
[                                 789 012 345 678 901 234 567 890 123 456 789 012 345 678 901 234 567 890 123 456 789 012 345 678 901 234 ]
#Indonesian_Local_rabbit_COX3     ACC ATA TAC CAA TGA TGA CGA GAC ATT GTA CGA GAA GGT ACA TTC CAA GGC CAC CAT ACT CCT ATT GTA CAA AAA GGT
#MH985853.1_NZW2_COX3             ... ... ... ... ... ... ... ... ... ... ... ... ... ... ... ... ... ... ... ... ... ... ... ... ... ...
#MN296708_YW_COX3                 ... ... ... ... ... ... ... ... ... ... ... ... ... ... ... ... ... ... ... ... ... ... ... ... ... ...
#AJ001588.1_NZW1_COX3             ... ... ... ... ... ... ... ... ... ... ... ... ... ... ... ... ... ... ... ... ... ... ... ... ... ...
#MN953621_CR_COX3                 ... ... ... ... ... ... ... ... ... ... ... ... ... ... ... ... ... ... ... ... ... ... ... ... ... ...

[                                 222 222 222 222 222 222 222 222 222 222 222 222 222 222 222 222 222 222 222 222 222 223 333 333 333 333 ]
[                                 333 334 444 444 444 555 555 555 566 666 666 667 777 777 777 888 888 888 899 999 999 990 000 000 000 111 ]
[                                 567 890 123 456 789 012 345 678 901 234 567 890 123 456 789 012 345 678 901 234 567 890 123 456 789 012 ]
#Indonesian_Local_rabbit_COX3     CTA CGA TAT GGC ATA ATT CTC TTT ATC ATC TCA GAA GTA TTC TTT TTC GCG GGC TTC TTC TGA GCC TTC TAC CAC TCA
#MH985853.1_NZW2_COX3             ... ... ... ... ... ... ... ... ... ... ... ... ... ... ... ... ... ... ... ... ... ... ... ... ... ...
#MN296708_YW_COX3                 ... ... ... ... ... ... ... ... ... ... ... ... ... ... ... ... ... ... ... ... ... ... ... ... ... ...
#AJ001588.1_NZW1_COX3             ... ... ... ... ... ... ... ... ... ... ... ... ... ... ... ... ... ... ... ... ... ... ... ... ... ...
#MN953621_CR_COX3                 ... ... ... ... ... ... ... ... ... ... ... ... ... ... ... ... ... ... ... ... ... ... ... ... ... ...
```

Genetic characteristics of complete mtDNA genome sequence of Indonesian Local rabbit (*Oryctolagus cuniculus*) (2023)
by: A. Setiaji, D. A. Lestari, N. S. Pandupuspitasari, I. Agusetyaningsih, F. A. Khan

```
[                                333 333 333 333 333 333 333 333 333 333 333 333 333 333 333 333 333 333 333 333 333 333 333 333 333 333 ]
[                                111 111 122 222 222 223 333 333 333 444 444 444 455 555 555 556 666 666 666 777 777 777 788 888 888 889 ]
[                                345 678 901 234 567 890 123 456 789 012 345 678 901 234 567 890 123 456 789 012 345 678 901 234 567 890 ]
#Indonesian_Local_rabbit_COX3    AGC CTA GCT CCT ACC CCA GAA CTA GGA GGA TGC TGA CCT CCA ACA GGC ATC AAC CCC CTT AAT CCC CTT GAA GTG CCG
#MH985853.1_NZW2_COX3            ... ... ... ... ... ... ... ... ... ... ... ... ... ... ... ... ... ... ... ... ... ... ... ... ... ...
#MN296708_YW_COX3                ... ... ... ... ... ... ... ... ... ... ... ... ... ... ... ... ... ... ... ... ... ... ... ... ... ...
#AJ001588.1_NZW1_COX3            ... ... ... ... ... ... ... ... ... ... ... ... ... ... ... ... ... ... ... ... ... ... ... ... ... ...
#MN953621_CR_COX3                ... ... ... ... ... ... ... ... ... ... ... ... ... ... ... ... ... ... ... ... ... ... ... ... ... ...

[                                333 333 333 444 444 444 444 444 444 444 444 444 444 444 444 444 444 444 444 444 444 444 444 444 444 444 ]
[                                999 999 999 000 000 000 011 111 111 112 222 222 222 333 333 333 344 444 444 445 555 555 555 666 666 666 ]
[                                123 456 789 012 345 678 901 234 567 890 123 456 789 012 345 678 901 234 567 890 123 456 789 012 345 678 ]
#Indonesian_Local_rabbit_COX3    CTA CTA AAC ACT TCA GTC CTC CTG GCT TCA GGA GTC TCA ATC ACC TGG GCC CAC CAC AGT TTA ATA GAA GGC AAT CGC
#MH985853.1_NZW2_COX3            ... ... ... ... ... ... ... ... ... ... ... ... ... ... ... ... ... ... ... ... ... ... ... ... ... ...
#MN296708_YW_COX3                ... ... ... ... ... ... ... ... ... ... ... ... ... ... ... ... ... ... ... ... ... ... ... ... ... ...
#AJ001588.1_NZW1_COX3            ... ... ... ... ... ... ... ... ... ... ... ... ... ... ... ... ... ... ... ... ... ... ... ... ... ...
#MN953621_CR_COX3                ... ... ... ... ... ... ... ... ... ... ... ... ... ... ... ... ... ... ... ... ... ... ... ... ... ...

[                                444 444 444 444 444 444 444 444 444 444 455 555 555 555 555 555 555 555 555 555 555 555 555 555 555 555 ]
[                                677 777 777 778 888 888 888 999 999 999 900 000 000 001 111 111 111 222 222 222 233 333 333 334 444 444 ]
[                                901 234 567 890 123 456 789 012 345 678 901 234 567 890 123 456 789 012 345 678 901 234 567 890 123 456 ]
#Indonesian_Local_rabbit_COX3    AAA AAC ATA CAG CAA GCT CTA GCC ATT ACC ATT CTC CTA GGT ATC TAT TTT ACC CTA CTT CAA GCA TCG GAG TAT TAC
#MH985853.1_NZW2_COX3            ... ... ... ... ... ... ... ... ... ... ... ... ... ... ... ... ... ... ... ... ... ... ... ... ... ...
#MN296708_YW_COX3                ... ... ... ... ... ... ... ... ... ... ... ... ... ... ... ... ... ... ... ... ... ... ... ... ... ...
#AJ001588.1_NZW1_COX3            ... ... ... ... ... ... ... ... ... ... ... ... ... ... ... ... ... ... ... ... ... ... ... ... ... ...
#MN953621_CR_COX3                ... ... ... ... ... ... ... ... ... ... ... ... ... ... ... ... ... ... ... ... ... ... ... ... ... ...

[                                555 555 555 555 555 555 555 555 555 555 555 555 555 555 555 555 555 556 666 666 666 666 666 666 666 666 ]
[                                444 555 555 555 566 666 666 667 777 777 777 888 888 888 899 999 999 990 000 000 000 111 111 111 122 222 ]
[                                789 012 345 678 901 234 567 890 123 456 789 012 345 678 901 234 567 890 123 456 789 012 345 678 901 234 ]
#Indonesian_Local_rabbit_COX3    GAA ACA TCT TTT ACT ATT TCA GAT GGA GTC TAC GGA TCA ACA TTC TTC ATA GCC ACA GGA TTT CAC GGT CTT CAC GTC
#MH985853.1_NZW2_COX3            ... ... ... ... ... ... ... ... ... ... ... ... ... ... ... ... ... ... ... ... ... ... ... ... ... ...
#MN296708_YW_COX3                ... ... ... ... ... ... ... ... ... ... ... ... ... ... ... ... ... ... ... ... ... ... ... ... ... ...
#AJ001588.1_NZW1_COX3            ... ... ... ... ... ... ... ... ... ... ... ... ... ... ... ... ... ... ... ... ... ... ... ... ... ...
#MN953621_CR_COX3                ... ... ... ... ... ... ... ... ... ... ... ... ... ... ... ... ... ... ... ... ... ... ... ... ... ...
```

Genetic characteristics of complete mtDNA genome sequence of Indonesian Local rabbit (*Oryctolagus cuniculus*) (2023)
by: A. Setiaji, D. A. Lestari, N. S. Pandupuspitasari, I. Agusetyaningsih, F. A. Khan

```
[                                 666 666 666 666 666 666 666 666 666 666 666 666 666 666 666 666 666 666 666 666 666 666 666 666 666 777 ]
[                                 222 223 333 333 333 444 444 444 455 555 555 556 666 666 666 777 777 777 788 888 888 889 999 999 999 000 ]
[                                 567 890 123 456 789 012 345 678 901 234 567 890 123 456 789 012 345 678 901 234 567 890 123 456 789 012 ]
#Indonesian_Local_rabbit_COX3     ATC ATT GGG TCC ACT TTT CTT ACG GTC TGC CTT CTA CGA CAA TTT AAC TTT CAC TTT ACA TCG AAC CAC CAC TTT GGT
#MH985853.1_NZW2_COX3             ... ... ... ... ... ... ... ... ... ... ... ... ... ... ... ... ... ... ... ... ... ... ... ... ... ...
#MN296708_YW_COX3                 ... ... ... ... ... ... ... ... ... ... ... ... ... ... ... ... ... ... ... ... ... ... ... ... ... ...
#AJ001588.1_NZW1_COX3             ... ... ... ... ... ... ... ... ... ... ... ... ... ... ... ... ... ... ... ... ... ... ... ... ... ...
#MN953621_CR_COX3                 ... ... ... ... ... ... ... ... ... ... ... ... ... ... ... ... ... ... ... ... ... ... ... ... ... ...

[                                 777 777 777 777 777 777 777 777 777 777 777 777 777 777 777 777 777 777 777 777 777 777 777 777 777 777 ]
[                                 000 000 011 111 111 112 222 222 222 333 333 333 344 444 444 445 555 555 555 666 666 666 677 777 777 778 ]
[                                 345 678 901 234 567 890 123 456 789 012 345 678 901 234 567 890 123 456 789 012 345 678 901 234 567 890 ]
#Indonesian_Local_rabbit_COX3     TTT GAA GCA GCC GCA TGA TAC TGA CAC TTT GTA GAT GTA GTC TGA CTA TTC CTA TAT GTA TCA ATC TAT TGA TGA GGG
#MH985853.1_NZW2_COX3             ... ... ... ... ... ... ... ... ... ... ... ... ... ... ... ... ... ... ... ... ... ... ... ... ... ...
#MN296708_YW_COX3                 ... ... ... ... ... ... ... ... ... ... ... ... ... ... ... ... ... ... ... ... ... ... ... ... ... ...
#AJ001588.1_NZW1_COX3             ... ... ... ... ... ... ... ... ... ... ... ... ... ... ... ... ... ... ... ... ... ... ... ... ... ...
#MN953621_CR_COX3                 ... ... ... ... ... ... ... ... ... ... ... ... ... ... ... ... ... ... ... ... ... ... ... ... ... ...

[                                 777 777 777 777 777 777 788 888 ]
[                                 888 888 888 999 999 999 900 000 ]
[                                 123 456 789 012 345 678 901 234 ]
#Indonesian_Local_rabbit_COX3     TCA TAC TCT TTT AGT ATC AAC TAG
#MH985853.1_NZW2_COX3             ... ... ... ... ... ... ... ...
#MN296708_YW_COX3                 ... .-- --- --- --- --- --- ---
#AJ001588.1_NZW1_COX3             ... .-- --- --- --- --- --- ---
#MN953621_CR_COX3                 ... .-- --- --- --- --- --- ---
```

Genetic characteristics of complete mtDNA genome sequence of Indonesian Local rabbit (*Oryctolagus cuniculus*) (2023)
by: A. Setiaji, D. A. Lestari, N. S. Pandupuspitasari, I. Agusetyaningsih, F. A. Khan

```
#MEGA
ATP8 rabbit alignment

[                                              111 111 111 122 222 222 223 333 333 333 444 444 444 455 555 555 556 666 666 666 777 777 777 ]
[                                  123 456 789 012 345 678 901 234 567 890 123 456 789 012 345 678 901 234 567 890 123 456 789 012 345 678 ]
#Indonesian_Local_rabbit_ATP8      ATG CCA CAA CTT GAC ACA TCC ACA TGA TTT ACT ACC ATT GTC GCC ATA ATT CTT TCA CTA TTT ATC CTA ATA CAA CTC
#MH985853.1_NZW2_ATP8              ... ... ... ... ... ... ... ... ... ... ... ... ... ... ... ... ... ... ... ... ... ... ... ... ... ...
#MN296708_YW_ATP8                  ... ... ... ... ... ... ... ... ... ... ... ... ... ... ... ... ... ... ... ... ... ... ... ... ... ...
#AJ001588.1_NZW1_ATP8              ... ... ... ... ... ... ... ... ... ... ... ... ... ... ... ... ... ... ... ... ... ... ... ... ... ...
#MN953621.1_CR_ATP8                ... ... ... ... ... ... ... ... ... ... ... ... ... ... ... ... ... ... ... ... ... ... ... ... ... ...

[                                                              111 111 111 111 111 111 111 111 111 111 111 111 111 111 111 111 111 111 111 ]
[                                  788 888 888 889 999 999 999 000 000 000 011 111 111 112 222 222 222 333 333 333 344 444 444 445 555 555 ]
[                                  901 234 567 890 123 456 789 012 345 678 901 234 567 890 123 456 789 012 345 678 901 234 567 890 123 456 ]
#Indonesian_Local_rabbit_ATP8      AAA TTC CAC AAA TAC ACA TAC CCT ATG AAC CCA GTA CTA AAA GCA CTT GAG TCT ACT TCA TTC CCT TGC CCA TGA GAA
#MH985853.1_NZW2_ATP8              ... ... ... ... ... ... ... ... ... ... ... ... ... ... ... ... ... ... ... ... ... ... ... ... ... ...
#MN296708_YW_ATP8                  ... ... ... ... ... ... ... ... ... ... ... ... ... ... ... ... ... ... ... ... ... ... ... ... ... ...
#AJ001588.1_NZW1_ATP8              ... ... ... ... ... ... ... ... ... ... ... ... ... ... ... ... ... ... ... ... ... ... ... ... ... ...
#MN953621.1_CR_ATP8                ... ... ... ... ... ... ... ... ... ... ... ... ... ... ... ... ... ... ... ... ... ... ... ... ... ...

[                                  111 111 111 111 111 111 111 111 111 111 111 111 111 111 122 222 ]
[                                  555 666 666 666 677 777 777 778 888 888 888 999 999 999 900 000 ]
[                                  789 012 345 678 901 234 567 890 123 456 789 012 345 678 901 234 ]
#Indonesian_Local_rabbit_ATP8      ACA AAA TGA ACG AAA ATT TAT TCT CCT CTT TCG CTA CCC CAA CAC TAA
#MH985853.1_NZW2_ATP8              ... ... ... ... ... ... ... ... ... ... ... ... ... ... ... ...
#MN296708_YW_ATP8                  ... ... ... ... ... ... ... ... ... ... ... ... ... ... ... ...
#AJ001588.1_NZW1_ATP8              ... ... ... ... ... ... ... ... ... ... ... ... ... ... ... ...
#MN953621.1_CR_ATP8                ... ... ... ... ... ... ... ... ... ... ... ... ... ... ... ...
```

Genetic characteristics of complete mtDNA genome sequence of Indonesian Local rabbit (*Oryctolagus cuniculus*) (2023)
by: A. Setiaji, D. A. Lestari, N. S. Pandupuspitasari, I. Agusetyaningsih, F. A. Khan

#MEGA
ATP6 rabbit alignment

```
[                                             111 111 111 122 222 222 223 333 333 333 444 444 444 455 555 555 556 666 666 666 777 777 777 ]
[                                 123 456 789 012 345 678 901 234 567 890 123 456 789 012 345 678 901 234 567 890 123 456 789 012 345 678 ]
#Indonesian_Local_rabbit_ATP6     ATG AAC GAA AAT TTA TTC TCC TCT TTC GCT ACC CCA ACA CTA ATA GGG CTC CCT ATT GTA GCC TTA ATT ATC ATA TTC
#MH985853_NZW2_ATP6               ... ... ... ... ... ... ... ... ... ... ... ... ... ... ... ... ... ... ... ... ... ... ... ... ... ...
#MN296708_YW_ATP6                 ... ... ... ... ... ... ... ... ... ... ... ... ... ... ... ... ... ... ... ... ... ... ... ... ... ...
#AJ001588.1_NZW1_ATP6             ... ... ... ... ... ... ... ... ... ... ... ... ... ... ... ... ... ... ... ... ... ... ... ... ... ...
#MN953621_CR_ATP6                 ... ... ... ... ... ... ... ... ... ... ... ... ... ... ... ... ... ... ... ... ... ... ... ... ... ...

[                                                             111 111 111 111 111 111 111 111 111 111 111 111 111 111 111 111 111 111 111 ]
[                                 788 888 888 889 999 999 999 000 000 000 011 111 111 112 222 222 222 333 333 333 344 444 444 445 555 555 ]
[                                 901 234 567 890 123 456 789 012 345 678 901 234 567 890 123 456 789 012 345 678 901 234 567 890 123 456 ]
#Indonesian_Local_rabbit_ATP6     CCA ACT TTA CTA TTT CCC TCC CCT AGC CGA CTA ATT AAC AAC CGA CTA GTC TCA ACC CAA CAA TGA TTA GCC CAA CTT
#MH985853_NZW2_ATP6               ... ... ... ... ... ... ... ... ... ... ... ... ... ... ... ... ... ... ... ... ... ... ... ... ... ...
#MN296708_YW_ATP6                 ... ... ... ... ... ... ... ... ... ... ... ... ... ... ... ... ... ... ... ... ... ... ... ... ... ...
#AJ001588.1_NZW1_ATP6             ... ... ... ... ... ... ... ... ... ... ... ... ... ... ... ... ... ... ... ... ... ... ... ... ... ...
#MN953621_CR_ATP6                 ... ... ... ... ... ... ... ... ... ... ... ... ... ... ... ... ... ... ... ... ... ... ... ... ... ...

[                                 111 111 111 111 111 111 111 111 111 111 111 111 111 111 122 222 222 222 222 222 222 222 222 222 222 222 ]
[                                 555 666 666 666 677 777 777 778 888 888 888 999 999 999 900 000 000 001 111 111 111 222 222 222 233 333 ]
[                                 789 012 345 678 901 234 567 890 123 456 789 012 345 678 901 234 567 890 123 456 789 012 345 678 901 234 ]
#Indonesian_Local_rabbit_ATP6     ATC TTA AAG CAA ATA ATA TTA ATA CAT TCC CCC AAA GGA CGA ACC TGA TCT CTA ATA CTA ATT TCC CTA ATC ATA TTT
#MH985853_NZW2_ATP6               ... ... ... ... ... ... ... ... ... ... ... ... ... ... ... ... ... ... ... ... ... ... ... ... ... ...
#MN296708_YW_ATP6                 ... ... ... ... ... ... ... ... ... ... ... ... ... ... ... ... ... ... ... ... ... ... ... ... ... ...
#AJ001588.1_NZW1_ATP6             ... ... ... ... ... ... ... ... ... ... ... ... ... ... ... ... ... ... ... ... ... ... ... ... ... ...
#MN953621_CR_ATP6                 ... ... ... ... ... ... ... ... ... ... ... ... ... ... ... ... ... ... ... ... ... ... ... ... ... ...

[                                 222 222 222 222 222 222 222 222 222 222 222 222 222 222 222 222 222 222 222 222 222 223 333 333 333 333 ]
[                                 333 334 444 444 444 555 555 555 566 666 666 667 777 777 777 888 888 888 899 999 999 990 000 000 000 111 ]
[                                 567 890 123 456 789 012 345 678 901 234 567 890 123 456 789 012 345 678 901 234 567 890 123 456 789 012 ]
#Indonesian_Local_rabbit_ATP6     ATT GGC TCA ACC AAC CTC CTA GGC CTC CTG CCT CAC TCA TTC ACA CCA ACA ACT CAA TTA TCA ATA AAT TTA GGG ATA
#MH985853_NZW2_ATP6               ... ... ... ... ... ... ... ... ... ... ... ... ... ... ... ... ... ... ... ... ... ... ... ... ... ...
#MN296708_YW_ATP6                 ... ... ... ... ... ... ... ... ... ... ... ... ... ... ... ... ... ... ... ... ... ... ... ... ... ...
#AJ001588.1_NZW1_ATP6             ... ... ... ... ... ... ... ... ... ... ... ... ... ... ... ... ... ... ... ... ... ... ... ... ... ...
#MN953621_CR_ATP6                 ... ... ... ... ... ... ... ... ... ... ... ... ... ... ... ... ... ... ... ... ... ... ... ... ... ...
```

```
[                                333 333 333 333 333 333 333 333 333 333 333 333 333 333 333 333 333 333 333 333 333 333 333 333 333 333 ]
[                                111 111 122 222 222 223 333 333 333 444 444 444 455 555 555 556 666 666 666 777 777 777 788 888 888 889 ]
[                                345 678 901 234 567 890 123 456 789 012 345 678 901 234 567 890 123 456 789 012 345 678 901 234 567 890 ]
#Indonesian_Local_rabbit_ATP6    GCA ATC CCC CTA TGA GCA GGA GCC GTA ATC ACC GGG TTT CGA TAC AAA ACT AAG GCG TCA CTA GCC CAT TTC CTC CCA
#MH985853_NZW2_ATP6              ... ... ... ... ... ... ... ... ... ... ... ... ... ... ... ... ... ... ... ... ... ... ... ... ... ...
#MN296708_YW_ATP6                ... ... ... ... ... ... ... ... ... ... ... ... ... ... ... ... ... ... ... ... ... ... ... ... ... ...
#AJ001588.1_NZW1_ATP6            ... ... ... ... ... ... ... ... ... ... ... ... ... ... ... ... ... ... ... ... ... ... ... ... ... ...
#MN953621_CR_ATP6                ... ... ... ... ... ... ... ... ... ... ... ... ... ... ... ... ... ... ... ... ... ... ... ... ... ...

[                                333 333 333 444 444 444 444 444 444 444 444 444 444 444 444 444 444 444 444 444 444 444 444 444 444 444 ]
[                                999 999 999 000 000 000 011 111 111 112 222 222 222 333 333 333 344 444 444 445 555 555 555 666 666 666 ]
[                                123 456 789 012 345 678 901 234 567 890 123 456 789 012 345 678 901 234 567 890 123 456 789 012 345 678 ]
#Indonesian_Local_rabbit_ATP6    CAA GGA ACC CCT ATT CCT CTT ATT CCT ATG CTA ATT GTT ATT GAA ACT ACT AGT CTC TTC ATT CAA CCT ATA GCC CTA
#MH985853_NZW2_ATP6              ... ... ... ... ... ... ... ... ... ... ... ... ... ... ... ... .T. ... ... ... ... ... ... ... ... ...
#MN296708_YW_ATP6                ... ... ... ... ... ... ... ... ... ... ... ... ... ... ... ... .T. ... ... ... ... ... ... ... ... ...
#AJ001588.1_NZW1_ATP6            ... ... ... ... ... ... ... ... ... ... ... ... ... ... ... ... .T. ... ... ... ... ... ... ... ... ...
#MN953621_CR_ATP6                ... ... ... ... ... ... ... ... ... ... ... ... ... ... ... ... .T. ... ... ... ... ... ... ... ... ...

[                                444 444 444 444 444 444 444 444 444 444 455 555 555 555 555 555 555 555 555 555 555 555 555 555 555 555 ]
[                                677 777 777 778 888 888 888 999 999 999 900 000 000 001 111 111 111 222 222 222 233 333 333 334 444 444 ]
[                                901 234 567 890 123 456 789 012 345 678 901 234 567 890 123 456 789 012 345 678 901 234 567 890 123 456 ]
#Indonesian_Local_rabbit_ATP6    GCC GTA CGG CTT ACA GCC AAT ATC ACA GCA GGC CAC CTT CTC ATA CAT CTT ATT GGA GGC GCT GCA CTA GCT TTA GTC
#MH985853_NZW2_ATP6              ... ... ... ... ... ... ... ... ... ... ... ... ... ... ... ... ... ... ... ... ... ... ... ... ... ...
#MN296708_YW_ATP6                ... ... ... ... ... ... ... ... ... ... ... ... ... ... ... ... ... ... ... ... ... ... ... ... ... ...
#AJ001588.1_NZW1_ATP6            ... ... ... ... ... ... ... ... ... ... ... ... ... ... ... ... ... ... ... ... ... ... ... ... ... ...
#MN953621_CR_ATP6                ... ... ... ... ... ... ... ... ... ... ... ... ... ... ... ... ... ... ... ... ... ... ... ... ... ...

[                                555 555 555 555 555 555 555 555 555 555 555 555 555 555 555 555 555 556 666 666 666 666 666 666 666 666 ]
[                                444 555 555 555 566 666 666 667 777 777 777 888 888 888 899 999 999 990 000 000 000 111 111 111 122 222 ]
[                                789 012 345 678 901 234 567 890 123 456 789 012 345 678 901 234 567 890 123 456 789 012 345 678 901 234 ]
#Indonesian_Local_rabbit_ATP6    TCA ATT AGT CCA ACA ACA GCC CTA ATT ACC TTT ATT ATC CTT ATT CTA CTA ACA ATT CTA GAA TTC GCC GTA GCC TTG
#MH985853_NZW2_ATP6              ... ... ... ... ... ... ... ... ... ... ... ... ... ... ... ... ... ... ... ... ... ... ... ... ... ...
#MN296708_YW_ATP6                ... ... ... ... ... ... ... ... ... ... ... ... ... ... ... ... ... ... ... ... ... ... ... ... ... ...
#AJ001588.1_NZW1_ATP6            ... ... ... ... ... ... ... ... ... ... ... ... ... ... ... ... ... ... ... ... ... ... ... ... ... ...
#MN953621_CR_ATP6                ... ... ... ... ... ... ... ... ... ... ... ... ... ... ... ... ... ... ... ... ... ... ... ... .T. ...
```

Genetic characteristics of complete mtDNA genome sequence of Indonesian Local rabbit (*Oryctolagus cuniculus*) (2023)
by: A. Setiaji, D. A. Lestari, N. S. Pandupuspitasari, I. Agusetyaningsih, F. A. Khan

```
[                                 666 666 666 666 666 666 666 666 666 666 666 666 666 666 666 666 666 666 666 ]
[                                 222 223 333 333 333 444 444 444 455 555 555 556 666 666 666 777 777 777 788 ]
[                                 567 890 123 456 789 012 345 678 901 234 567 890 123 456 789 012 345 678 901 ]
#Indonesian_Local_rabbit_ATP6     ATC CAA GCT TAC GTC TTT ACT CTC CTT GTA AGC CTA TAC CTA CAT GAC AAT ACC TAA
#MH985853_NZW2_ATP6               ... ... ... ... ... ... ... ... ... ... ... ... ... ... ... ... ... ... ...
#MN296708_YW_ATP6                 ... ... ... ... ... ... ... ... ... ... ... ... ... ... ... ... ... ... ..-
#AJ001588.1_NZW1_ATP6             ... ... ... ... ... ... ... ... ... ... ... ... ... ... ... ... ... ... ..-
#MN953621_CR_ATP6                 ... ... ... ... ... ... ... ... ... ... ... ... ... ... ... ... ... ... ...
```

```
#MEGA
CYTB rabbit alignment

[                                            111 111 111 122 222 222 223 333 333 333 444 444 444 455 555 555 556 666 666 666 777 777 777 ]
[                                123 456 789 012 345 678 901 234 567 890 123 456 789 012 345 678 901 234 567 890 123 456 789 012 345 678 ]
#Indonesian_Local_rabbit_CYTB    ATG ACC AAC ATT CGC AAA ACC CAC CCC CTA TTA AAA ATT GTT AAC CAC TCC CTA ATT GAC CTT CCT GCT CCA TCA AAC
#MH985853_NZW2_CYTB              ... ... ... ... ... ... ... ... ... ... ... ... ... ... ... ... ... ... ... ... ... ... ... ... ... ...
#MN296708_YW_CYTB                ... ... ... ... ... ... ... ... ... ... ... ... ... ... ... ... ... ... ... ... ... ... ... ... ... ...
#AJ001588.1_NZW1_CYTB            ... ... ... ... ... ... ... ... ... ... ... ... ... ... ... ... ... ... ... ... ... ... ... ... ... ...
#MN953621_CR_CYTB                ... ... ... ... ... ... ... ... ... ... ... ... ... ... ... ... ... ... ... ... ... ... ... ... ... ...

[                                                                111 111 111 111 111 111 111 111 111 111 111 111 111 111 111 111 111 111 111 ]
[                                788 888 888 889 999 999 999 000 000 000 011 111 111 112 222 222 222 333 333 333 344 444 444 445 555 555 ]
[                                901 234 567 890 123 456 789 012 345 678 901 234 567 890 123 456 789 012 345 678 901 234 567 890 123 456 ]
#Indonesian_Local_rabbit_CYTB    ATC TCT GCC TGA TGA AAC TTT GGC TCT CTA CTA GGC CTG TGC CTT ATA ATT CAA ATT TTC ACT GGC CTA TTC TTA GCC
#MH985853_NZW2_CYTB              ... ... ... ... ... ... ... ... ... ... ... ... ... ... ... ... ... ... ... ... ... ... ... ... ... ...
#MN296708_YW_CYTB                ... ... ... ... ... ... ... ... ... ... ... ... ... ... ... ... ... ... ... ... ... ... ... ... ... ...
#AJ001588.1_NZW1_CYTB            ... ... ... ... ... ... ... ... ... ... ... ... ... ... ... ... ... ... ... ... ... ... ... ... ... ...
#MN953621_CR_CYTB                ... ... ... ... ... ... ... ... ... ... ... ... ... ... ... ... ... ... ... ... ... ... ... ... ... ...

[                                111 111 111 111 111 111 111 111 111 111 111 111 111 111 122 222 222 222 222 222 222 222 222 222 222 222 ]
[                                555 666 666 666 677 777 777 778 888 888 888 999 999 999 900 000 000 001 111 111 111 222 222 222 233 333 ]
[                                789 012 345 678 901 234 567 890 123 456 789 012 345 678 901 234 567 890 123 456 789 012 345 678 901 234 ]
#Indonesian_Local_rabbit_CYTB    ATA CAC TAC ACC TCT GAC ACA ACA ACA GCA TTC TCA TCA GTA ACC CAT ATT TGC CGA GAT GTT AAC TAT GGC TGA CTT
#MH985853_NZW2_CYTB              ... ... ... ... ... ... ... ... ... ... ... ... ... ... ... ... ... ... ... ... ... ... ... ... ... ...
#MN296708_YW_CYTB                ... ... ... ... ... ... ... ... ... ... ... ... ... ... ... ... ... ... ... ... ... ... ... ... ... ...
#AJ001588.1_NZW1_CYTB            ... ... ... ... ... ... ... ... ... ... ... ... ... ... ... ... ... ... ... ... ... ... ... ... ... ...
#MN953621_CR_CYTB                ... ... ... ... ... ... ... ... ... ... ... ... ... ... ... ... ... ... ... ... ... ... ... ... ... ...

[                                222 222 222 222 222 222 222 222 222 222 222 222 222 222 222 222 222 222 222 222 222 223 333 333 333 333 ]
[                                333 334 444 444 444 555 555 555 566 666 666 667 777 777 777 888 888 888 899 999 999 990 000 000 000 111 ]
[                                567 890 123 456 789 012 345 678 901 234 567 890 123 456 789 012 345 678 901 234 567 890 123 456 789 012 ]
#Indonesian_Local_rabbit_CYTB    ATC CGA TAC CTC CAC GCT AAC GGA GCA TCT ATA TTC TTT ATT TGC CTC TAC ATA CAC GTA GGC CGC GGA ATC TAC TAT
#MH985853_NZW2_CYTB              ... ... ... ... ... ... ... ... ... ... ... ... ... ... ... ... ... ... ... ... ... ... ... ... ... ...
#MN296708_YW_CYTB                ... ... ... ... ... ... ... ... ... ... ... ... ... ... ... ... ... ... ... ... ... ... ... ... ... ...
#AJ001588.1_NZW1_CYTB            ... ... ... ... ... ... ... ... ... ... ... ... ... ... ... ... ... ... ... ... ... ... ... ... ... ...
#MN953621_CR_CYTB                ... ... ... ... ... ... ... ... ... ... ... ... ... ... ... ... ... ... ... ... ... ... ... ... ... ...
```

Genetic characteristics of complete mtDNA genome sequence of Indonesian Local rabbit (*Oryctolagus cuniculus*) (2023)
by: A. Setiaji, D. A. Lestari, N. S. Pandupuspitasari, I. Agusetyaningsih, F. A. Khan

```
[                               333 333 333 333 333 333 333 333 333 333 333 333 333 333 333 333 333 333 333 333 333 333 333 333 333 333 ]
[                               111 111 122 222 222 223 333 333 333 444 444 444 455 555 555 556 666 666 666 777 777 777 788 888 888 889 ]
[                               345 678 901 234 567 890 123 456 789 012 345 678 901 234 567 890 123 456 789 012 345 678 901 234 567 890 ]
#Indonesian_Local_rabbit_CYTB   GGA TCA TAC ACA TAC CTA GAG ACC TGA AAC ATT GGC ATC ATC CTC CTA TTC GCA GTA ATA GCC ACA GCA TTT ATG GGT
#MH985853_NZW2_CYTB             ... ... ... ... ... ... ... ... ... ... ... ... ... ... ... ... ... ... ... ... ... ... ... ... ... ...
#MN296708_YW_CYTB               ... ... ... ... ... ... ... ... ... ... ... ... ... ... ... ... ... ... ... ... ... ... ... ... ... ...
#AJ001588.1_NZW1_CYTB           ... ... ... ... ... ... ... ... ... ... ... ... ... ... ... ... ... ... ... ... ... ... ... ... ... ...
#MN953621_CR_CYTB               ... ... ... ... ... ... ... ... ... ... ... ... ... ... ... ... ... ... ... ... ... ... ... ... ... ...

[                               333 333 333 444 444 444 444 444 444 444 444 444 444 444 444 444 444 444 444 444 444 444 444 444 444 444 ]
[                               999 999 999 000 000 000 011 111 111 112 222 222 222 333 333 333 344 444 444 445 555 555 555 666 666 666 ]
[                               123 456 789 012 345 678 901 234 567 890 123 456 789 012 345 678 901 234 567 890 123 456 789 012 345 678 ]
#Indonesian_Local_rabbit_CYTB   TAT GTC CTC CCA TGA GGA CAA ATA TCA TTT TGG GGA GCA ACC GTA ATC ACT AAC CTC CTA TCA GCA ATC CCA TAT ATC
#MH985853_NZW2_CYTB             ... ... ... ... ... ... ... ... ... ... ... ... ... ... ... ... ... ... ... ... ... ... ... ... ... ...
#MN296708_YW_CYTB               ... ... ... ... ... ... ... ... ... ... ... ... ... ... ... ... ... ... ... ... ... ... ... ... ... ...
#AJ001588.1_NZW1_CYTB           ... ... ... ... ... ... ... ... ... ... ... ... ... ... ... ... ... ... ... ... ... ... ... ... ... ...
#MN953621_CR_CYTB               ... ... ... ... ... ... ... ... ... ... ... ... ... ... ... ... ... ... ... ... ... ... ... ... .W. ...

[                               444 444 444 444 444 444 444 444 444 444 455 555 555 555 555 555 555 555 555 555 555 555 555 555 555 555 ]
[                               677 777 777 778 888 888 888 999 999 999 900 000 000 001 111 111 111 222 222 222 233 333 333 334 444 444 ]
[                               901 234 567 890 123 456 789 012 345 678 901 234 567 890 123 456 789 012 345 678 901 234 567 890 123 456 ]
#Indonesian_Local_rabbit_CYTB   GGA ACA ACC TTA GTT GAA TGA ATC TGA GGA GGA TTT TCA GTT GAT AAA GCC ACT CTT ACC CGA TTC TTC GCT TTT CAC
#MH985853_NZW2_CYTB             ... ... ... ... ... ... ... ... ... ... ... ... ... ... ... ... ... ... ... ... ... ... ... ... ... ...
#MN296708_YW_CYTB               ... ... ... ... ... ... ... ... ... ... ... ... ... ... ... ... ... ... ... ... ... ... ... ... ... ...
#AJ001588.1_NZW1_CYTB           ... ... ... ... ... ... ... ... ... ... ... ... ... ... ... ... ... ... ... ... ... ... ... ... ... ...
#MN953621_CR_CYTB               ... ... ... ... ... ... ... ... ... ... ... ... ... ... ... ... ... ... ... ... ... ... ... ... ... ...

[                               555 555 555 555 555 555 555 555 555 555 555 555 555 555 555 555 555 556 666 666 666 666 666 666 666 666 ]
[                               444 555 555 555 566 666 666 667 777 777 777 888 888 888 899 999 999 990 000 000 000 111 111 111 122 222 ]
[                               789 012 345 678 901 234 567 890 123 456 789 012 345 678 901 234 567 890 123 456 789 012 345 678 901 234 ]
#Indonesian_Local_rabbit_CYTB   TTC ATC TTG CCA TTT ATC ATT GCA GCT TTA GTC TTA ATT CAC CTC CTC TTT CTA CAT GAA ACT GGC TCC AAC AAC CCC
#MH985853_NZW2_CYTB             ... ... ... ... ... ... ... ... A.. ... ... ... ... ... ... ... ... ... ... ... ... ... ... ... ... ...
#MN296708_YW_CYTB               ... ... ... ... ... ... ... ... A.. ... ... ... ... ... ... ... ... ... ... ... ... ... ... ... ... ...
#AJ001588.1_NZW1_CYTB           ... ... ... ... ... ... ... ... A.. ... ... ... ... ... ... ... ... ... ... ... ... ... ... ... ... ...
#MN953621_CR_CYTB               ... ... ... ... ... ... ... ... A.. ... ... ... ... ... ... ... ... ... ... ... ... ... ... ... ... ...
```

Genetic characteristics of complete mtDNA genome sequence of Indonesian Local rabbit (*Oryctolagus cuniculus*) (2023)
by: A. Setiaji, D. A. Lestari, N. S. Pandupuspitasari, I. Agusetyaningsih, F. A. Khan

```
[                                 666 666 666 666 666 666 666 666 666 666 666 666 666 666 666 666 666 666 666 666 666 666 666 666 666 777 ]
[                                 222 223 333 333 333 444 444 444 455 555 555 556 666 666 666 777 777 777 788 888 888 889 999 999 999 000 ]
[                                 567 890 123 456 789 012 345 678 901 234 567 890 123 456 789 012 345 678 901 234 567 890 123 456 789 012 ]
#Indonesian_Local_rabbit_CYTB     ACA GGA ATT CCT TCA AAC TCA GAT AAA ATC CCT TTC CAC CCC TAC TAC ACA ATC AAA GAC ACC CTA GGT TTC CTT GTA
#MH985853_NZW2_CYTB               ... ... ... ... ... ... ... ... ... ... ... ... ... ... ... ... ... ... ... ... ... ... ... ... ... ...
#MN296708_YW_CYTB                 ... ... ... ... ... ... ... ... ... ... ... ... ... ... ... ... ... ... ... ... ... ... ... ... ... ...
#AJ001588.1_NZW1_CYTB             ... ... ... ... ... ... ... ... ... ... ... ... ... ... ... ... ... ... ... ... ... ... ... ... ... ...
#MN953621_CR_CYTB                 ... ... ... ... ... .G. ... ... ... ... ... ... ... ... ... ... ... ... ... ... ... ... ... ... ... ...

[                                 777 777 777 777 777 777 777 777 777 777 777 777 777 777 777 777 777 777 777 777 777 777 777 777 777 777 ]
[                                 000 000 011 111 111 112 222 222 222 333 333 333 344 444 444 445 555 555 555 666 666 666 677 777 777 778 ]
[                                 345 678 901 234 567 890 123 456 789 012 345 678 901 234 567 890 123 456 789 012 345 678 901 234 567 890 ]
#Indonesian_Local_rabbit_CYTB     GCC ATT CTT CTC CTC CTT ATT TTA GTC CTA TTT TCA CCA GAC CTA TTA GGA GAC CCA GAC AAC TAC ACC CCT GCC AAC
#MH985853_NZW2_CYTB               ... ... ... ... ... ... ... ... ... ... ... ... ... ... ... ... ... ... ... ... ... ... ... ... ... ...
#MN296708_YW_CYTB                 ... ... ... ... ... ... ... ... ... ... ... ... ... ... ... ... ... ... ... ... ... ... ... ... ... ...
#AJ001588.1_NZW1_CYTB             ... ... ... ... ... ... ... ... ... ... ... ... ... ... ... ... ... ... ... ... ... ... ... ... ... ...
#MN953621_CR_CYTB                 ... ... ... ... ... ... ... ... ... ... ... ... ... ... ... ... ... ... ... ... ... ... ... ... ... ...

[                                 777 777 777 777 777 777 788 888 888 888 888 888 888 888 888 888 888 888 888 888 888 888 888 888 888 888 ]
[                                 888 888 888 999 999 999 900 000 000 001 111 111 111 222 222 222 233 333 333 334 444 444 444 555 555 555 ]
[                                 123 456 789 012 345 678 901 234 567 890 123 456 789 012 345 678 901 234 567 890 123 456 789 012 345 678 ]
#Indonesian_Local_rabbit_CYTB     CCC CTT AAT ACC CCT CCC CAT ATC AAA CCA GAA TGA TAC TTT CTA TTT GCC TAC GCT ATC CTA CGC TCT ATT CCA AAT
#MH985853_NZW2_CYTB               ... ... ... ... ... ... ... ... ... ... ... ... ... ... ... ... ... ... ... ... ... ... ... ... ... ...
#MN296708_YW_CYTB                 ... ... ... ... ... ... ... ... ... ... ... ... ... ... ... ... ... ... ... ... ... ... ... ... ... ...
#AJ001588.1_NZW1_CYTB             ... ... ... ... ... ... ... ... ... ... ... ... ... ... ... ... ... ... ... ... ... ... ... ... ... ...
#MN953621_CR_CYTB                 ... ... ... ... ... ... ... ... ... ... ... ... ... ... ... ... ... ... ... ... ... ... ... ... ... ...

[                                 888 888 888 888 888 888 888 888 888 888 888 888 888 889 999 999 999 999 999 999 999 999 999 999 999 999 ]
[                                 566 666 666 667 777 777 777 888 888 888 899 999 999 990 000 000 000 111 111 111 122 222 222 223 333 333 ]
[                                 901 234 567 890 123 456 789 012 345 678 901 234 567 890 123 456 789 012 345 678 901 234 567 890 123 456 ]
#Indonesian_Local_rabbit_CYTB     AAA CTC GGA GGA GTC CTA GCT CTA GTC CTA TCC ATC CTT GTT CTA GCC TTC ATC CCA TTC CTT CAT ATG TCT AAA CAA
#MH985853_NZW2_CYTB               ... ... ... ... ... ... ... ... ... ... ... ... ... ... ... ... ... ... ... ... ... ... ... ... ... ...
#MN296708_YW_CYTB                 ... ... ... ... ... ... ... ... ... ... ... ... ... ... ... ... ... ... ... ... ... ... ... ... ... ...
#AJ001588.1_NZW1_CYTB             ... ... ... ... ... ... ... ... ... ... ... ... ... ... ... ... ... ... ... ... ... ... ... ... ... ...
#MN953621_CR_CYTB                 ... ... ... ... ... ... ... ... ... ... ... ... ... ... ... ... ... ... ... ... ... ... ... ... ... ...
```

Genetic characteristics of complete mtDNA genome sequence of Indonesian Local rabbit (*Oryctolagus cuniculus*) (2023)
by: A. Setiaji, D. A. Lestari, N. S. Pandupuspitasari, I. Agusetyaningsih, F. A. Khan

```
[                                                                                                                   111 111 111 111 111 ]
[                                 999 999 999 999 999 999 999 999 999 999 999 999 999 999 999 999 999 999 999 999 999 000 000 000 000 000 ]
[                                 333 444 444 444 455 555 555 556 666 666 666 777 777 777 788 888 888 889 999 999 999 000 000 000 011 111 ]
[                                 789 012 345 678 901 234 567 890 123 456 789 012 345 678 901 234 567 890 123 456 789 012 345 678 901 234 ]
#Indonesian_Local_rabbit_CYTB     CGT AGC ATG ATA TTC CGA CCC ATT AGT CAA GTC CTA TTC TGA GTT CTC GTC GCA GAT CTT CTC ACA CTC ACA TGA ATC
#MH985853_NZW2_CYTB               ... ... ... ... ... ... ... ... ... ... ... ... ... ... ... ... ... ... ... ... ... ... ... ... ... ...
#MN296708_YW_CYTB                 ... ... ... ... ... ... ... ... ... ... ... ... ... ... ... ... ... ... ... ... ... ... ... ... ... ...
#AJ001588.1_NZW1_CYTB             ... ... ... ... ... ... ... ... ... ... ... ... ... ... ... ... ... ... ... ... ... ... ... ... ... ...
#MN953621_CR_CYTB                 ... ... ... ... ... ... ... ... ... ... ... ... ... ... ... ... ... ... ... ... ... ... ... ... ... ...

[                                 111 111 111 111 111 111 111 111 111 111 111 111 111 111 111 111 111 111 111 111 111 111 111 111 111 111 ]
[                                 000 000 000 000 000 000 000 000 000 000 000 000 000 000 000 000 000 000 000 000 000 000 000 000 000 000 ]
[                                 111 112 222 222 222 333 333 333 344 444 444 445 555 555 555 666 666 666 677 777 777 778 888 888 888 999 ]
[                                 567 890 123 456 789 012 345 678 901 234 567 890 123 456 789 012 345 678 901 234 567 890 123 456 789 012 ]
#Indonesian_Local_rabbit_CYTB     GGA GGC CAA CCA GTA GAA CAC CCG TTC ATC ACC ATT GGA CAA GTA GCA TCT GTC CTC TAC TTC ACC ACC ATC CTT ATT
#MH985853_NZW2_CYTB               ... ... ... ... ... ... ... ... ... ... ... ... ... ... ... ... ... ... ... ... ... ... ... ... ... ...
#MN296708_YW_CYTB                 ... ... ... ... ... ... ... ... ... ... ... ... ... ... ... ... ... ... ... ... ... ... ... ... ... ...
#AJ001588.1_NZW1_CYTB             ... ... ... ... ... ... ... ... ... ... ... ... ... ... ... ... ... ... ... ... ... ... ... ... ... ...
#MN953621_CR_CYTB                 ... ... ... ... ... ... ... ... ... ... ... ... ... ... ... ... ... ... ... ... ... ... ... ... ... ...

[                                 111 111 111 111 111 111 111 111 111 111 111 111 111 111 111 111 ]
[                                 000 000 011 111 111 111 111 111 111 111 111 111 111 111 111 111 ]
[                                 999 999 900 000 000 001 111 111 111 222 222 222 233 333 333 334 ]
[                                 345 678 901 234 567 890 123 456 789 012 345 678 901 234 567 890 ]
#Indonesian_Local_rabbit_CYTB     CTT ATA CCC CTC GCA AGC CTA ATC GAA AAC AAA ATC CTC AAA TGA AGG
#MH985853_NZW2_CYTB               ... ... ... ... ... ... ... ... ... ... ... ... ... ... ... ...
#MN296708_YW_CYTB                 ... ... ... ... ... ... ... ... ... ... ... ... ... ... ... ...
#AJ001588.1_NZW1_CYTB             ... ... ... ... ... ... ... ... ... ... ... ... ... ... ... ..-
#MN953621_CR_CYTB                 ... ... ... .C. ... ... ... ... ... ... ... ... ... ... ... ...
```
